# Evaluating the Causal Relation of ApoA-IV with Disease-Related Traits - A Bidirectional Two-sample Mendelian Randomization Study

**DOI:** 10.1038/s41598-017-07213-9

**Published:** 2017-08-18

**Authors:** Salome Mack, Stefan Coassin, Julien Vaucher, Florian Kronenberg, Claudia Lamina, ApoA-IV-GWAS Consortium, Rico Rueedi, Noha A. Yousri, Ilkka Seppälä, Christian Gieger, Sebastian Schönherr, Lukas Forer, Gertraud Erhart, Barbara Kollerits, Pedro Marques-Vidal, Martina Müller-Nurasyid, Gerard Waeber, Sven Bergmann, Doreen Dähnhardt, Andrea Stöckl, Stefan Kiechl, Olli T. Raitakari, Mika Kähönen, Johann Willeit, Ludmilla Kedenko, Bernhard Paulweber, Annette Peters, Thomas Meitinger, Konstantin Strauch, Terho Lehtimäki, Steven C. Hunt, Peter Vollenweider

**Affiliations:** 1 Division of Genetic Epidemiology, Department of Medical Genetics, Molecular and Clinical Pharmacology, Medical University of Innsbruck, Innsbruck, 6020 Austria; 2 Department of Internal Medicine, Lausanne University Hospital, Lausanne, 1015 Switzerland; 3 Department of Computational Biology, University of Lausanne, 1015 Lausanne, Switzerland; 4 Swiss Institute of Bioinformatics, 1015 Lausanne, Switzerland; 5 Department of Physiology and Biophysics, Weill Cornell Medical College–Qatar, Doha, Qatar; 6 Department of Computer and Systems Engineering, Alexandria University, 21526 Alexandria, Egypt; 7 Department of Clinical Chemistry, Fimlab Laboratories and University of Tampere School of Medicine, 33520 Tampere, Finland; 8 Institute of Genetic Epidemiology, Helmholtz Zentrum München—German Research Center for Environmental Health, 85764 Neuherberg, Germany; 9 Institute of Epidemiology II, Helmholtz Zentrum München—German Research Center for Environmental Health, 85764 Neuherberg, Germany; 10 Research Unit of Molecular Epidemiology, Helmholtz Zentrum München—German Research Center for Environmental Health, 85764 Neuherberg, Germany; 11 DZHK (German Centre for Cardiovascular Research), partner site Munich Heart Alliance, 80802 Munich, Germany; 12 Institute of Medical Informatics, Biometry and Epidemiology, Chair of Genetic Epidemiology, Ludwig-Maximilians-Universität, 81377 Munich, Germany; 13 Department of Medicine I, University Hospital Grosshadern, Ludwig-Maximilians-Universität, 81377 Munich, Germany; 14 Department of Neurology, Medical University of Innsbruck, 6020 Innsbruck, Austria; 15 Department of Clinical Physiology, Turku University Hospital, 20520 Turku, Finland; 16 Research Centre of Applied and Preventive Cardiovascular Medicine, University of Turku, 20520 Turku, Finland; 17 Department of Clinical Physiology, Tampere University Hospital and University of Tampere, 33521 Tampere, Finland; 18 First Department of Internal Medicine, Paracelsus Private Medical University, 5020 Salzburg, Austria; 19 German Center for Diabetes Research (DZD e.V.), 85764 Neuherberg, Germany; 20 Institute of Human Genetics, Technische Universität München, 81675 München, Germany; 21 Institute of Human Genetics, Helmholtz Zentrum München, 85764 Neuherberg, Germany; 22 Munich Cluster for Systems Neurology (SyNergy), 81377 Munich, Germany; 23 Cardiovascular Genetics Division, University of Utah School of Medicine, Salt Lake City, UT 84108 USA; 24 Department of Genetic Medicine, Weill Cornell Medicine, Doha, Qatar

## Abstract

Apolipoprotein A-IV (apoA-IV) has been observed to be associated with lipids, kidney function, adiposity- and diabetes-related parameters. To assess the causal relationship of apoA-IV with these phenotypes, we conducted bidirectional Mendelian randomization (MR) analyses using publicly available summary-level datasets from GWAS consortia on apoA-IV concentrations (n = 13,813), kidney function (estimated glomerular filtration rate (eGFR), n = 133,413), lipid traits (HDL cholesterol, LDL cholesterol, triglycerides, n = 188,577), adiposity-related traits (body-mass-index (n = 322,206), waist-hip-ratio (n = 210,088)) and fasting glucose (n = 133,010). Main analyses consisted in inverse-variance weighted and multivariable MR, whereas MR-Egger regression and weighted median estimation were used as sensitivity analyses. We found that eGFR is likely to be causal on apoA-IV concentrations (53 SNPs; causal effect estimate per 1-SD increase in eGFR = −0.39; 95% CI = [−0.54, −0.24]; p-value = 2.4e-07). Triglyceride concentrations were also causally associated with apoA-IV concentrations (40 SNPs; causal effect estimate per 1-SD increase in triglycerides = −0.06; 95% CI = [−0.08, −0.04]; p-value = 4.8e-07), independently of HDL-C and LDL-C concentrations (causal effect estimate from multivariable MR = −0.06; 95% CI = [−0.10, −0.02]; p-value = 0.0014). Evaluating the inverse direction of causality revealed a possible causal association of apoA-IV on HDL-cholesterol (2 SNPs; causal effect estimate per one percent increase in apoA-IV = −0.40; 95% CI = [−0.60, −0.21]; p-value = 5.5e-05).

## Introduction

Apolipoprotein A-IV (apoA-IV) has been discussed as a biomarker and /or risk factor for cardiovascular diseases, kidney disease and diabetes^1–4^. Whether a measurable trait is a causal risk factor or a marker acting as an indicator for the presence of a disease or its severity, is one of the key questions in biomedical research^5, 6^. To act as a biomarker for a certain disease, causality is not strictly required, since a biomarker can be regulated as a response to a disease and/or an associated trait already in a very early stage of the disease. It can therefore act as a disease predictor without being causally involved in the pathogenesis of the disease. Moreover, the association between a biomarker and a disease may be confounded by measured or unmeasured factors that distort their relationship. However, establishing causality between a measurable trait and a disease is crucial to understand the pathophysiological mechanisms.

Disease endpoints are often detected using continuous surrogate markers (e.g. fasting glucose as marker for impaired glucose metabolism leading to diabetes) or are influenced by many other intermediate phenotypes, which might better represent the causal pathway leading to a disease than the disease state itself. With this motivation in mind, we evaluated the causal relation of apoA-IV with disease-related, continuous surrogate markers and intermediate phenotypes for which evidence from observational association studies or functional experiments proposes a functional link with apoA-IV.

ApoA-IV is a glycoprotein, which is a component of triglyceride-rich and high-density lipoproteins but it also circulates free in human plasma^7^. Its physiological role is not fully elucidated, yet. It participates in reverse cholesterol transport^8, 9^ and plays an important role in relieving peripheral cells of an overload of cholesterol^10, 11^. It has anti-atherogenic properties^12, 13^ and low concentrations were found to be associated with cardiovascular outcomes such as coronary heart disease or acute coronary syndrome^2, 14–16^. About 30% of the phenotypic variance has been shown to be genetically regulated^17^. In a recent genome-wide association study (GWAS) meta-analysis we have identified two variants within the *APOA5-A4-C3-A1* cluster and one variant in the *KLKB1* gene to be associated with apoA-IV concentrations^17^. Furthermore, the lead *APOA4*-SNP was shown to be associated with HDL cholesterol (HDL-C) concentrations. Other variants within the *APOA5-A4-C3-A1* cluster have also been shown to be associated both with HDL-C, but also with triglyceride (TG) concentrations^18–20^. In addition, a TG genetic score was found to be associated with apoA-IV concentrations^17^. Studies in animal models, genetic association studies and studies evaluating disease progression argue for apoA-IV as the driving factor influencing satiety, adiposity, glucose concentrations and chronic kidney disease^21–28^. On the other hand apoA-IV concentrations have also been shown to change following food intake, gastric bypass surgery or weight loss^29, 30^. Those findings warrant further investigation in causal associations and especially in the direction of the effects between apoA-IV and lipid levels, adiposity, diabetes-related parameters and kidney function. Recent developments in genetic epidemiology using genetic markers as proxies for a trait help estimate causal associations with a disease outcome through a Mendelian randomization approach^31^.

Such causal effects are usually very small. Therefore, single studies are often underpowered. However, results of genome-wide association studies are increasingly made publicly available. Harnessing summary-level data, Mendelian randomization analyses then reach sufficient statistical power to yield more precise causal effect estimates^32^.

For all of the already mentioned apoA-IV-associated and disease-related traits, large genome-wide association studies are available, in which dozens of genetic variants have been identified^18, 33–36^. Furthermore, a GWAS meta-analysis on apoA-IV concentrations has been published recently^17^.

Therefore, we conducted a bidirectional two-sample Mendelian randomization analysis to assess the causal relationship of apoA-IV concentrations with HDL-C, LDL cholesterol (LDL-C), TG, fasting glucose (FG) as well as body-mass-index (BMI), waist-hip-ratio adjusted for BMI (WHR), and estimated glomerular filtration rate (eGFR). Herein, lipid levels, adiposity-related traits, glucose and eGFR were considered as risk factors on apoA-IV concentrations as outcome variable. We complemented the analysis by investigating the inverse association of whether apoA-IV is causally associated with the same traits. We applied different sensitivity analyses to account for presence of potential pleiotropy.

## Results

### Validity of instrumental variables and power analysis

**Figure 1:**
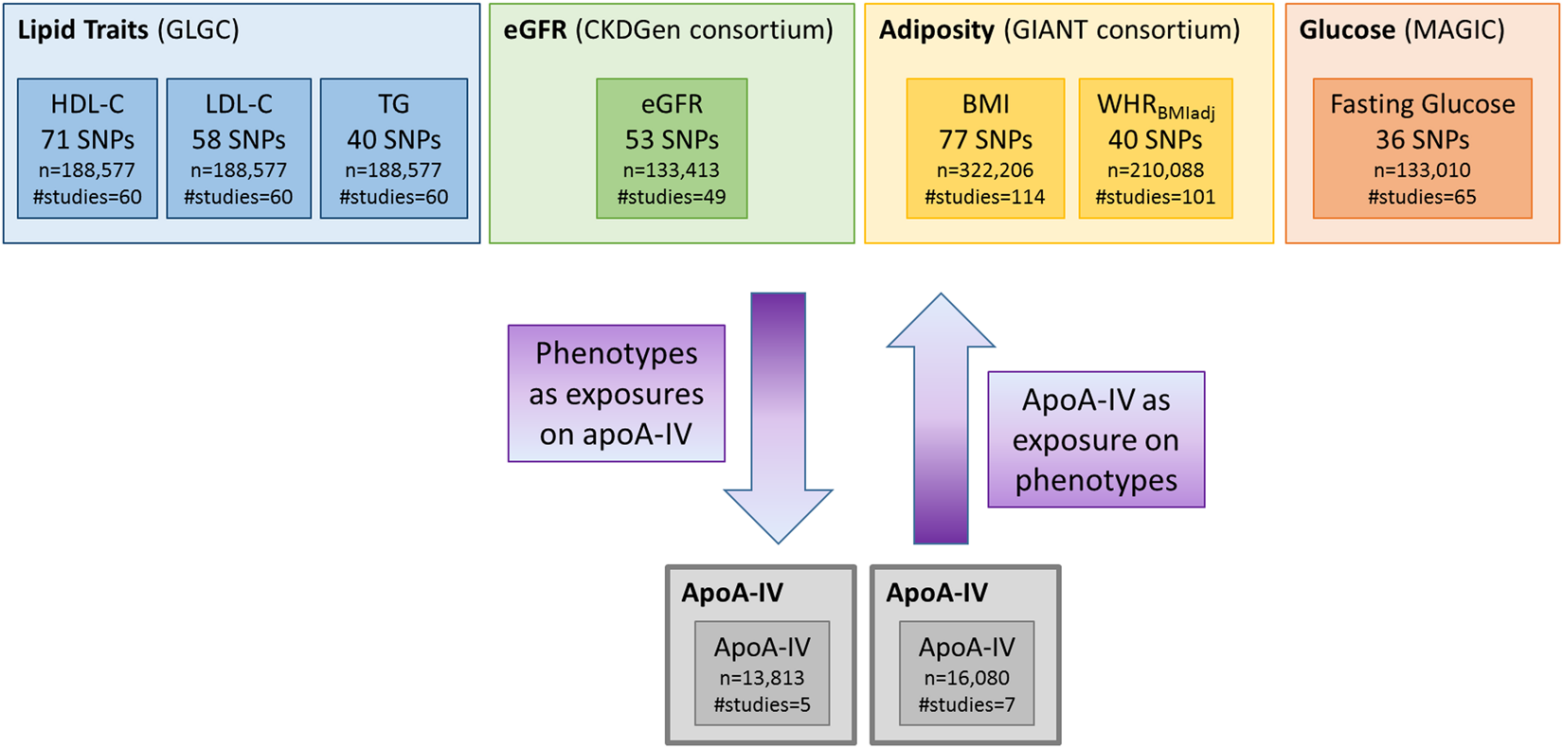
Graphical overview of the study design. Seven biomarkers out of four consortia (GLGC^18^; CKDGen consortium^33^; GIANT consortium^34, 60^; MAGIC^35, 36^) were included as well as results from a GWAS meta-analysis on apoA-IV^17^.

A graphical overview of the design and studies used in our investigation is given in Fig. 1. To assess the strength of the instrumental variables (i.e. the single-nucleotide polymorphisms [SNPs]) that were used for the Mendelian Randomization, the explained variance (R²) defined by the SNPs is given for each of the investigated potential risk factors in Tables 1 and 2. R² varies between 0.0060 (WHR) and 0.0727 (LDL-C).

**Table 1:** Strength of instrumental variables used for the MR analyses: Explained variance (R²) of SNPs on the different exposure traits and detectable causal explained variance (R^2^) of the exposure traits on apoA-IV as outcome.

Phenotype (Exposure trait)	No. of SNPs	N exposure dataset	N outcome dataset	R² of SNPs on exposure in %	Detectable R^2^ of exposure on outcome in %*
HDL-C	71	188,577	13,813	6.76	1.37
LDL-C	58	188,577	13,813	7.27	1.27
TG	40	188,577	13,813	3.25	2.85
eGFR	53	133,413	13,813	3.22	2.88
BMI	77	322,206	13,813	2.41	3.84
WHR	40	210,088	13,813	0.60	15.44
Fasting Glucose	36	133,010	13,813	3.57	2.60

*Power = 80%; significance level α = 0.00625; sample size n = N outcome dataset.

**Table 2:** Strength of instrumental variables used for the MR analyses: Explained variance (R²) of SNPs on apoA-IV as exposure trait and detectable causal explained variance (R^2^) of apoA-IV as exposure trait on the different outcomes.

Phenotype (Outcome trait)	No. of SNPs	N exposure dataset	N outcome dataset	R² of SNPs on exposure in %	Detectable R^2^ of exposure on outcome in %*
HDL-C	2	16,080	188,577	2.00	0.34
LDL-C	2	16,080	188,577	2.00	0.34
TG	2	16,080	188,577	2.00	0.34
eGFR	2	16,080	133,413	2.00	0.48
BMI	2	16,080	322,206	2.00	0.20
WHR	2	16,080	210,088	2.00	0.30
Fasting Glucose	3	16,080	133,010	2.90	0.48

*Power = 80%; significance level α = 0.00625; sample size n = N outcome dataset.

Table 1 shows, which fraction of detectable causal R² of the exposure trait on the outcome (i.e. which magnitude of causal effect of the trait on the outcome) can be detected for the calculated R² of the genetic variants on the exposure (for HDL-C to fasting glucose as the exposure trait, apoA-IV is the exposure and for apoA-IV as the exposure trait, the other investigated traits function as the outcome). It varies between 0.0127 for LDL-C and 0.1544 for WHR, assuming a significance level of α = 0.00625 and a power of 80%. That is, to detect a causal effect of WHR on apoA-IV, WHR would have to explain more than 15% of the phenotypic variance of apoA-IV. For the reverse causation (effect of apoA-IV on the other factors), causal effects ranging between 0.002 and 0.0048 would be detectable (Table 2).

### Mendelian randomization analyses

#### Causal association of lipid traits on apoA-IV

An overview about the characteristics and the meta-analysis results of the 71 HDL-SNPs, the 58 LDL-SNPs and the 40 triglyceride-SNPs can be found in Supplementary Tables 1–3.

**Figure 2:**
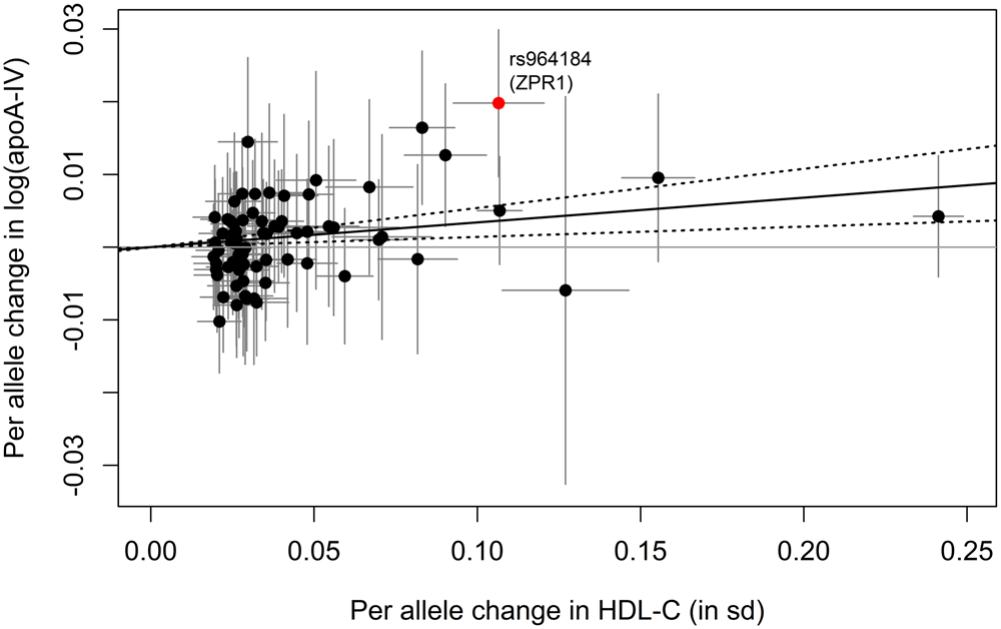
Scatterplot showing the effect estimates of SNP-HDL-C (high-density lipoprotein cholesterol) associations (95% CI) on the x- and SNP-apoA-IV (apolipoprotein A-IV) associations (95% CI) on the y-axis for all 71 SNPs. The continuous black line represents the MR-IVW estimate of HDL-C on apoA-IV (dashed lines represent corresponding 95% CI). The SNP rs964184 (*ZPR1*, formerly known as *ZNF259*), which was identified to be potentially pleiotropic is marked in red.

Using the MR-IVW method revealed a significant association of **HDL-C levels** (in SD) with the log-transformed apoA-IV concentrations resulting in a causal estimate of β_MR-IVW_ = 0.0341 (95% CI = [0.0142, 0.0540], p-value = 0.0008, Table 3 and Fig. 2). This means an increase in one SD in HDL-C levels (≈17 mg/dL) leads to an increase of the log-transformed apoA-IV levels of approximately 0.034, which corresponds to a relative increase of 3.4%. After adjusting for LDL-C as well as TG in the multivariable Mendelian randomization using all 71 SNPs, this causal effect diminished and became non-significant (β_adjusted_ = 0.0195, 95% CI = [−0.0084, 0.0475], p-value = 0.1706, see Table 3). One SNP was identified to be potentially pleiotropic by the gtx-package in R (rs964184 within the gene region *APOA5-A4-C3-A1*). This is the same SNP that we planned to exclude as a sensitivity analysis due to its close proximity to the *APOA4* gene (see Methods). After exclusion of this SNP, the effect became non-significant (β_MR-IVW_gtx_ = 0.0271, 95% CI = [0.0067, 0.0474], p-value = 0.0091, see Table 3). By adjusting for LDL-C and TG, the beta estimate remained approximately the same and was non-significant, too (Table 3). Excluding the 23 SNPs that were in LD with SNPs associated with any of the other traits (Supplementary Table 8) and applying the MR-IVW method to the remaining 48 SNPs did not change the causal estimate obtained using all 71 SNPs, but it became non-significant (p = 0.0453, Supplementary Table 15 and Supplementary Figure 4). The application of the multivariable Mendelian randomization approach to the 61 SNPs not in LD with SNPs from the non-lipid traits gave comparable results to using the adjustment model on all 71 SNPs (p = 0.1212, Supplementary Table 15 and Supplementary Figure 5). Using the MR-Egger regression method resulted in a markedly higher and statistically significant causal effect of β_MR-Egger_ = 0.0611 (95% CI = [0.0254, 0.0969], p-value = 0.0008, see Supplementary Table 16, Supplementary Figure 15), but also with a broader confidence interval. However, when using a weighted median estimation approach, the causal effect diminished and became non-significant (Supplementary Table 16).

**Table 3:** MR-IVW estimates for all phenotypes assumed to causally affect the apoA-IV concentrations. Here, the phenotypes act as exposures and apoA-IV as the outcome. A p-value smaller than 0.05/8 = 0.00625 is considered to be significant (marked in bold).

Phenotype (exposure)	Causal effect of phenotype on apoA-IV							
	All SNPs				Removing SNP rs964184 for lipids			
	No. of SNPs	Beta*	95% CI*	P-value	No. of SNPs	Beta*	95% CI*	P-value
*Lipid traits*								
HDL-C, unadjusted	71	0.0341	[0.0142, 0.0540]	**0.0008**	70	0.0271	[0.0067, 0.0474]	0.0091
HDL-C, adjusted for LDL-C and TG (multivariable MR)	71	0.0195	[−0.0084, 0.0475]	0.1706	70	0.0262	[−0.0018, 0.0542]	0.0672
LDL-C, unadjusted	58	−0.0376	[−0.0572, −0.0179]	**0.0002**	57	−0.0320	[−0.0519, −0.0121]	**0.0016**
LDL-C, adjusted for HDL-C and TG (multivariable MR)	58	−0.0225	[−0.0441, −0.0010]	0.0406	57	−0.0259	[−0.0480, −0.0039]	0.0213
TG, unadjusted	40	−0.0600	[−0.0834, −0.0366]	**4.8e-07**	39	−0.0498	[−0.0776, −0.0220]	**0.0004**
TG, adjusted for HDL-C and LDL-C (multivariable MR)	40	−0.0597	[−0.0962, −0.0232]	**0.0014**	39	−0.0487	[−0.0883, −0.0090]	0.0161
*Kidney function*								
eGFR	53	−0.3890	[−0.5367, −0.2413]	**2.4e-07**				
*Adiposity-related parameters*								
BMI	77	0.0067	[−0.0266, 0.0400]	0.6946				
WHR	40	0.0761	[0.0093, 0.1429]	0.0255				
*Fasting Glucose*								
Fasting Glucose	36	0.0146	[−0.0364, 0.0656]	0.5742				

*In log(apoA-IV) per change in SD for the lipid and obesity traits, in log(apoA-IV) per change in log(eGFR) for kidney function, in log(apoA-IV) per change in mmol/L for fasting glucose.

**Figure 3:**
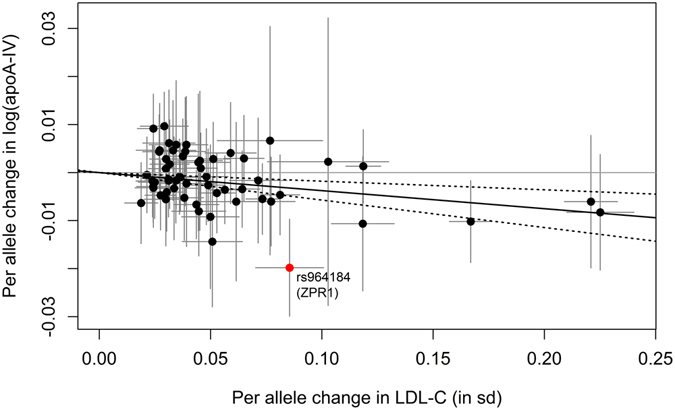
Scatterplot showing the effect estimates of SNP-LDL-C (low-density lipoprotein cholesterol) associations (95% CI) on the x- and SNP-apoA-IV (apolipoprotein A-IV) associations (95% CI) on the y-axis for all 58 SNPs. The continuous black line represents the MR-IVW estimate of LDL-C on apoA-IV (dashed lines represent the corresponding 95% CI). The SNP rs964184 (*ZPR1*, formerly known as *ZNF259*), which was excluded in a sensitivity analysis is marked in red.

For **LDL-C levels**, we obtained a significant causal estimate of β_MR-IVW_ = −0.0376 (95% CI = [−0.0572, −0.0179], p-value = 0.0002, Table 3 and Fig. 3) by using the MR-IVW method. This means that LDL-C levels and the log-transformed apoA-IV concentrations are inversely correlated and an increase in LDL-C will result in a decrease of apoA-IV levels. Adjusting for HDL-C as well as TG in the multivariable Mendelian randomization using all 58 SNPs reduced the size of the causal effect estimate and it became non-significant (β_adjusted_ = −0.0225, 95% CI = [−0.0441, −0.0010], p-value = 0.0406, see Table 3). Using the gtx-package in R, no heterogeneous SNP could be detected. After exclusion of the above mentioned SNP rs964184 and application of the MR-IVW method based on the remaining 57 SNPs, we obtained a significant causal estimate comparable to the MR-IVW estimate we got using all 58 SNPs (Table 3). By adjusting for HDL-C and TG, the beta estimate remained nearly the same but it became non-significant β_adjusted_ = −0.0259 (95% CI = [−0.0480, −0.0039], p-value = 0.0213, see Table 3). Excluding the 12 SNPs that were in LD with SNPs associated with any of the other traits (Supplementary Table 9) and applying the MR-IVW method to the remaining 46 SNPs did not markedly change the causal estimate obtained using all 58 SNPs, but it became non-significant (p = 0.0144, Supplementary Table 15 and Supplementary Figure 6). The application of the multivariable Mendelian randomization approach to the 57 SNPs not in LD with SNPs from the non-lipid traits gave comparable results to using the adjustment on all 58 SNPs (p = 0.0395, Supplementary Table 15 and Supplementary Figure 7). Using MR-Egger regression and the weighted median estimation method, LDL-C remained associated with apoA-IV (Supplementary Table 16 and Supplementary Figure 16).

**Figure 4:**
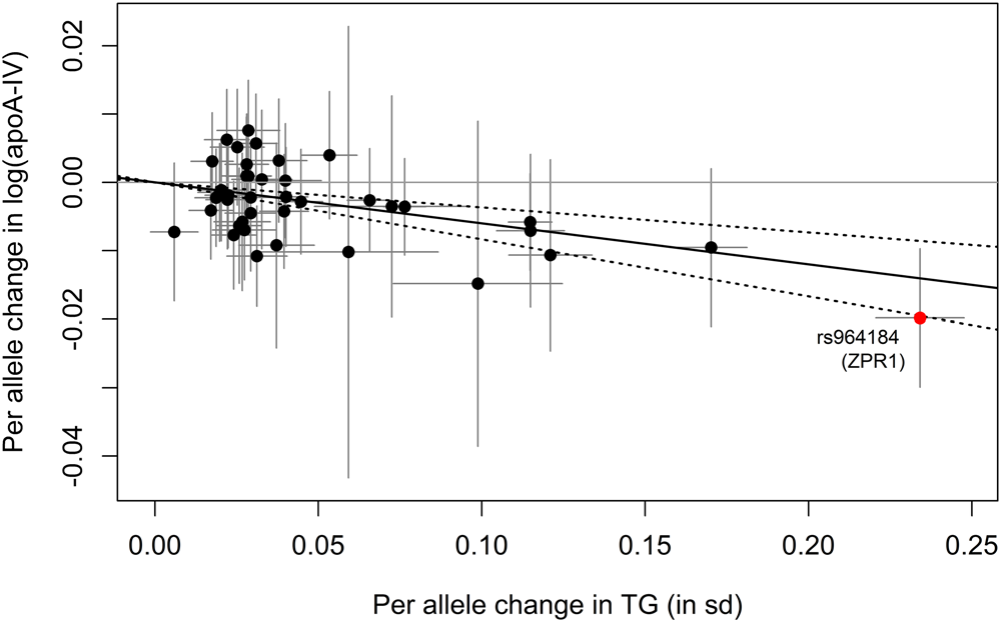
Scatterplot showing the effect estimates of SNP-TG (triglycerides) associations (95% CI) on the x- and SNP-apoA-IV (apolipoprotein A-IV) associations (95% CI) on the y-axis for all 40 SNPs. The continuous black line represents the MR-IVW estimate of TG on apoA-IV (dashed lines represent the corresponding 95% CI). The SNP rs964184 (*ZPR1*, formerly known as *ZNF259*), which was excluded in a sensitivity analysis is marked in red.

Taking the 40 SNPs associated with **triglyceride levels** and using the MR-IVW method, a significant causal estimate of β_MR-IVW_ = −0.0600 (95% CI = [−0.0834, −0.0366], p-value = 4.80 × 10^−7^, Table 3 and Fig. 4) was obtained. As for LDL-C levels, an increase in TG levels will result in a decrease of apoA-IV concentrations. Adjusting for HDL-C and LDL-C in the multivariable MR resulted in nearly the same statistically significant causal estimate (β_adjusted_ = −0.0597, 95% CI = [−0.0962, −0.0232], p-value = 0.0014, see Table 3). By applying the gtx-package in R, no heterogeneous SNP could be detected. Excluding the 23 SNPs that were in LD with SNPs associated with any of the other traits (Supplementary Table 10) and applying the MR-IVW method to the remaining 17 SNPs did not change the causal estimate obtained using all 40 SNPs, but it became non-significant (p = 0.1003, Supplementary Table 15 and Supplementary Figure 8). The application of the multivariable Mendelian randomization approach to the 33 SNPs not in LD with SNPs from the non-lipid traits resulted in a significant causal estimate comparable to the estimate obtained using the adjustment on all 58 SNPs (p = 0.0047, Supplementary Table 15 and Supplementary Figure 9). All other sensitivity analyses, i.e. the exclusion of SNP rs964184 as well as the application of the MR-Egger regression or the weighted median estimation method resulted all in nearly the same statistically significant causal estimate (Table 3 and Supplementary Table 16). Looking at the funnel plot and the individual SNP-based contributions to the MR analysis, the causal estimate of one SNP (rs838880, within *SCARB1*) attracted our attention as it harbored a potential bias due to its low level of precision (Supplementary Figure 17). However, excluding this SNP from the MR-IVW analysis as well as from the MR-Egger regression analysis did not change the overall causal estimate as the precision of this one IV estimate was very low (data not shown).

Concerning directional pleiotropy, MR-Egger intercepts did not depart significantly from the origin for all investigated lipid traits, meaning that no directional bias could be detected in all cases (Supplementary Table 17). Assessment of the NOME assumption gave I²_GX_ = 0.98 for all investigated lipid traits, suggesting an approximate 2% attenuation of the causal estimate towards zero due to measurement error in the exposure trait. The bias adjustment via SIMEX did not change the causal MR-Egger estimate noticeably (Supplementary Table 18).

#### Causal association of kidney function (eGFR) on apoA-IV

**Figure 5:**
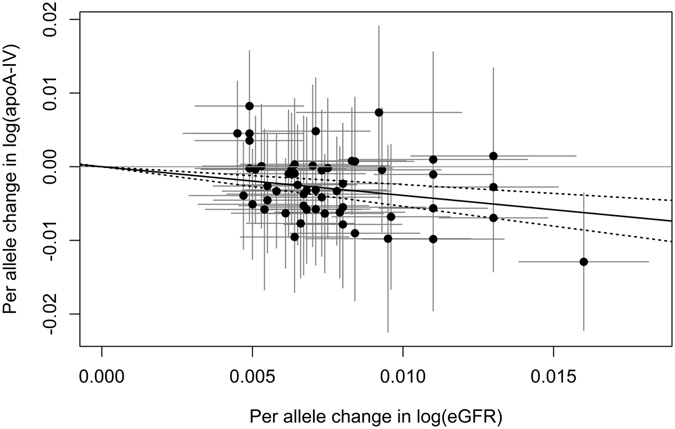
Scatterplot showing the effect estimates of SNP-eGFR (estimated glomerular filtration rate) associations (95% CI) on the x- and SNP-apoA-IV (apolipoprotein A-IV) associations (95% CI) on the y-axis for all 53 SNPs. The continuous black line represents the MR-IVW estimate of eGFR on apoA-IV (dashed lines represent the corresponding 95% CI).

An overview about the characteristics and the meta-analysis results of the 53 SNPs found to be associated with eGFR can be found in Supplementary Table 4. Using the MR-IVW method for analyzing the influence of kidney function on the log-transformed apoA-IV concentrations resulted in a statistically significant causal estimate of β_MR-IVW_ = −0.3890 (95% CI = [−0.5367, −0.2413], p-value = 2.44 × 10^−7^, see Table 3 and Fig. 5). Consequently, an increase in eGFR will reduce the apoA-IV concentrations. Using the gtx-package in R, no heterogeneous SNP could be detected. Excluding the 4 SNPs that were in LD with SNPs associated with any of the other traits (Supplementary Table 11) and applying the MR-IVW method to the remaining 49 SNPs did not change the causal estimate obtained using all 53 SNPs at all (Supplementary Table 15 and Supplementary Figure 10). The causal estimate obtained using MR-Egger regression gave an even higher significant effect and the significant weighted median estimate was between the other two estimates in size (Supplementary Table 16 and Supplementary Figure 18).

Using the MR-Egger regression, no directional pleiotropy could be detected for the analysis of eGFR on the log-transformed apoA-IV concentrations (Supplementary Table 17). Assessment of the NOME assumption gave I²_GX_ = 0.82, suggesting an approximate 15% to 20% attenuation of the causal estimate towards zero due to measurement error in the exposure trait. Therefore, the bias adjustment via SIMEX did result in a slightly stronger negative effect of the corrected causal MR-Egger estimate (Supplementary Table 18).

#### Causal association of the adiposity-related traits and fasting glucose on apoA-IV

An overview about the characteristics and the meta-analysis results of the 77 BMI-SNPs, the 40 WHR-SNPs and the 36 glucose-SNPs can be found in Supplementary Tables 5–7. All three factors did not show a significant association on the log-transformed apoA-IV concentrations neither by using the MR-IVW approach based on all SNPs or after excluding potentially pleiotropic SNPs using the proxy search (Table 3, Supplementary Tables 12–14 and Supplementary Figures 11–13) nor by using the MR-Egger regression or the weighted median estimation approach (Supplementary Table 16, Supplementary Figures 1–3 and Supplementary Figures 19–21). Using the gtx-package in R, no heterogeneous SNPs could be detected. The MR-Egger regression intercept estimate was non-significant for all of these variables, such that no directional pleiotropy could be detected (Supplementary Table 17).

#### Causal association of apoA-IV on the other traits

The characteristics and meta-analysis results of the 3 SNPs found to be associated with apoA-IV can be found in Supplementary Table 19. The investigation of apoA-IV as exposure on the other traits revealed only one significant finding. Two of the three SNPs that were found to be associated with apoA-IV were present in the HDL-C dataset. Using this data in a MR analysis using the MR-IVW method resulted in a significant causal estimate of β_MR-IVW_ = −0.4017 (95% CI = [−0.5970, −0.2064], p-value = 5.52e-05, see Table 4). Therefore, an increase in apoA-IV will result in a decrease in HDL-C. For the other traits, none of the causal estimates were significant (p-values ranging between 0.0754 and 0.8930, Table 4). Assessment of the NOME assumption gave I²_GX_ = 0.99, suggesting an approximate 1% attenuation of the causal estimate towards zero due to measurement error in the exposure trait (Supplementary Table 18). Therefore, bias due to measurement error in the exposure trait is negligible.

**Table 4:** MR-IVW estimates for all phenotypes assumed to be causally affected by apoA-IV concentrations.

Phenotype (outcome)	Causal effect of apoA-IV on phenotype			
	No. of SNPs	Beta^§^	95% CI^§^	P-value
*Lipid traits*				
HDL-C, unadjusted	2	−0.4017	[−0.5970, −0.2064]	**5.52e-05**
LDL-C, unadjusted	2	0.1931	[−0.0197, 0.4059]	0.0754
TG, unadjusted	2	−0.0761	[−0.2677, 0.1154]	0.4360
*Kidney function*				
eGFR	2	0.0207	[−0.0136, 0.0550]	0.2369
*Adiposity-related parameters*				
BMI	2	−0.0325	[−0.1840, 0.1190]	0.6744
WHR	2	0.0119	[−0.1609, 0.1847]	0.8930
*Fasting Glucose*				
Fasting Glucose	3	0.0462	[−0.0708, 0.1631]	0.4392

^§^In SD per change in log(apoA-IV) for the lipid and obesity traits, in log(eGFR) per change in log(apoA-IV) for kidney function, in mmol/L per change in log(apoA-IV) for fasting glucose. Here, apoA-IV acts as exposure and the phenotypes as the outcome traits. A p-value smaller than 0.05/8 = 0.00625 is considered to be significant (marked in bold).

## Discussion

The evaluation of the causal relations of apoA-IV with disease-related traits using Mendelian randomization analyses revealed three major findings: The most interesting finding was that eGFR is likely to causally influence apoA-IV concentrations. Furthermore, TG was found to affect apoA-IV concentrations, whereas apoA-IV concentrations seem to influence HDL-C.

The main Mendelian randomization analysis as well as all sensitivity analyses consistently showed that an increase in eGFR is associated with a decrease in apoA-IV concentrations. The investigation in the reverse direction, i.e. whether apoA-IV levels are causal on eGFR, did not result in a statistically significant finding. This was at the first glance surprising since prior studies found apoA-IV as a predictor of CKD progression independent of the GFR measured at baseline^25^. It has been suggested that other properties of apoA-IV not related to the filtration capacity of the kidney might explain the association with CKD progression^25^. If apoA-IV concentrations change already very early in the progression of kidney impairment, apoA-IV may still serve as a valuable marker for kidney disease and disease progression, which has been found in earlier cross-sectional studies^22–24^. On the other hand, knowing that chronic kidney disease is intimately associated with risk of cardiovascular disease^37^, this finding is interesting to stimulate research on biological pathways that link decrease in kidney function with apoA-IV levels.

The second main finding was that TG causally affects apoA-IV, even after adjusting for HDL-C and LDL-C. However, in the sensitivity analysis excluding the potentially pleiotropic SNP rs964184, the causal effect calculated using the MR-IVW method resulted in a statistically non-significant estimate. In this setting, this was not surprising, as this SNP was the one with the highest effect size on both TG and log(apoA-IV). However, it is only mandatory to exclude this SNP from the analysis, if it violates the instrumental variable assumption. This would be the case, if it influenced apoA-IV concentrations directly or through an endogenous variable that is different from the exposure. It was not identified to be pleiotropic by the applied statistical methods (gtx-package), though. Although being located within the *APOA5-A4-C3-A1* gene cluster, this SNP is rather independent from the *APOA4*-SNPs associated with apoA-IV concentrations, as already discussed in Lamina *et al*.^17^. Rs964184 is located in the 3′ UTR of *ZRP1* (formerly known as *ZNF259*), approximately 50 kb downstream of *APOA4*, and shows r^2^ < 0.3 with SNPs in *APOA4* (according to 1000 Genomes, phase 3; Supplementary Figure 14). It presents, however, a moderate linkage disequilibrium with SNPs in *APOA5* (max. r^2^ = 0.52 for rs2266788; Supplementary Figure 14), which is a major regulator of plasma triglyceride concentration^38^. Expression QTLs (eQTLs, i.e. SNPs associated with expression of a gene) reported in GTEx for *BUD13, ZPR1* and all apolipoprotein genes in the *APOA5-A4-C3-A1* gene cluster were not in linkage disequilibrium (LD) with rs964184 (maximum pair-wise LD was with eQTL rs4225 for *APOA1*: r^2^ = 0.2; D′ = 0.08; single SNP data not shown). Together this suggests that rs964184 is neither in LD with SNPs in the *APOA4* gene nor with any known eQTL in the gene cluster, but presents modest LD with SNPs in *APOA5*. It is therefore unlikely that rs964184 has a direct effect on apoA-IV concentrations and it would be too conservative to exclude it from the analysis. It is also not surprising that the exclusion of the 23 SNPs, which are included in the SNP sets of the other traits or in LD with SNPs of the other traits, resulted in a non-significant causal estimate. Among these 23 SNPs were the most informative SNPs, i.e. SNPs with the highest effect on TG and therefore also on apoA-IV. Not even half of the triglyceride SNPs remain, which is primarily due to being in LD with HDL-C SNPs. The multivariable method is the most useful method to account for this interrelation with other lipid traits by simultaneously keeping the most informative SNPs. Therefore, we additionally excluded only SNPs that were in LD with SNPs from the non-lipid traits, but adjusted for HDL-C and LDL-C. Using this method, the causal estimate from triglycerides on apoA-IV remained statistically significant. Nevertheless, this relationship has to be interpreted with caution. Further studies are needed to confirm this potentially causal association of TG and apoA-IV.

The investigation of HDL-C and LDL-C as causal factors influencing apoA-IV concentrations yielded inconsistent results. In previous genetic studies, several loci have been associated with apoA-IV as well as HDL-C and LDL-C^17–19^. Besides, a study investigating the LDL-C lowering response to different diets found that the apoA-IV protein isoforms modulate the LDL-C lowering response to a diet^39^. However, we demonstrated recently in a study with more than 13,000 individuals, that the genetic variants that are the molecular basis for the apoA-IV isoforms do not have an effect on apoA-IV concentrations. In the data at hand, the MR-IVW method and the MR-Egger regression revealed a statistically significant positive causal estimate, but the effect vanished after adjustment for the other lipid traits. It is therefore conceivable that the effect of both HDL-C as well as LDL-C on apoA-IV concentrations is triggered primarily by the association with TG. Furthermore, 5 SNPs are present in all three lipid datasets (rs12748152, rs964184, rs174546, rs3764261, rs2954029) which might also influence this result. The exploration of the reverse causation, i.e. whether apoA-IV influences HDL-C, resulted in a significant finding, showing an inverse causal direction. However, this result is triggered mainly by one SNP in the *APOA4* gene (rs1729407). This SNP was associated with both apoA-IV concentrations as well as HDL-C. Since it was the top signal in the GWAS on apoA-IV concentrations^17^ but not genome-wide significantly associated with HDL-C^18^, although conducted in a much higher sample size, it is conceivable that it exerts an effect primarily on apoA-IV. However, a direct effect on HDL-C cannot be excluded which would violate the exclusion-restriction assumption of Mendelian randomization. Given that apoA-IV is an important component of the HDL-C particle^21, 40–42^, a causal effect of apoA-IV on HDL-C level is possible and partly supported by our data, but has to be considered with caution.

Investigating the adiposity-related traits resulted in no significant findings. This is in contrast to the literature on observational epidemiological and functional studies: apoA-IV has been proposed as a satiety factor and related to diet-induced adiposity, both in animal models^21^ and in humans^26^. In addition, the administration of apoA-IV resulted in reduction of food intake and genetic association studies have found associations between polymorphisms within the *APOA4* gene and adiposity-related traits^27, 28^. Although these findings argument in favor of apoA-IV as a risk factor, it has also been shown that apoA-IV is decreased following weight loss and/or gastric bypass^29, 30^. Despite the epidemiological and also functional studies linking adiposity-related traits to apoA-IV, we could not find a causal effect in one or the other direction. One reason for that might be that – despite the already huge datasets and the high number of associated SNPs – the power is still insufficient to detect a causal effect from especially WHR as exposure on apoA-IV as outcome variable. To be detectable in our analysis with a power of 80%, the causal effect of WHR on apoA-IV would have to be that high, that it explains at least 15% of the phenotypic variance of apoA-IV (Table 1). The detectable explained variance is in a more realistic range for BMI (about 3.8%), but still higher than for the other investigated phenotypes. For the other direction of causality (apoA-IV as exposure for adiposity), however, lack of power should not be an issue. Even such small effects as 0.2–0.3% of explained variance should have been possible to detect. Therefore, it is rather unlikely, that apoA-IV causally influences BMI or WHR. It has to be noted that the choice of WHR and BMI as factors of satiety might not be optimal. However, they are the most commonly used markers for adiposity, for which there is also sufficient information on genetic loci influencing these traits and were therefore chosen for the evaluation of adiposity-related traits.

Counter-intuitively, we found no causal association of fasting glucose with apoA-IV and vice versa. This should not be a problem of power like for the adiposity-related traits, as the effect that could be detected with the given data is even smaller than for TG and eGFR. Already much is known that links glucose with apoA-IV. Diabetics have been found to have significantly higher apoA-IV levels^26, 43^. Apart from that, apoA-IV was inversely associated with prediabetes (defined by fasting glucose levels) and also with 2 h glucose levels^4^. Experimental studies and *APOA4* knockout mice have also shown that apoA-IV has a glucose-lowering effect^29, 44^. Therefore, we would have expected to find a causal association of the apoA-IV SNPs (where all 3 SNPs were present in the dataset from Manning *et al*.) on fasting glucose. In this case, apoA-IV would have to explain only more than 0.48% of the phenotypic variance of fasting glucose to have a power of 80%. Still, we could not find a causal association with any of the methods used in any of the investigated directions. Not finding a significant association does not exclude the possibility that there still exists one: apoA-IV and fasting glucose might still be causally related with each other but the true effect might be smaller than what can be detected with our data. The other possibility to explain this non-significant finding might be that the observed association of apoA-IV with glucose is confounded by other factors that influence both traits.

Using summary-level data of all SNPs that have been shown to be genome-wide significantly associated with their respective traits as instrumental variables, is both a strength but also a limitation of our study. By using summarized data, a higher power can be achieved than in single studies. The availability of dozens of associated SNPs that were identified in more than 100,000 participants does not guarantee to get a high power for the Mendelian randomization analysis, though. For the adiposity-related traits as risk/protective factors for apoA-IV, the instrumental variables do not explain much of the variation in these traits. Therefore, to be able to find a causal association of a reasonable size, further SNPs would have to be identified that can be used to increase the variance explained on BMI and especially WHR. For the lipid traits, however, our applied methods based on summarized data achieved a high power enabling the detection of very low causal effects. Nevertheless, not only high power is necessary for Mendelian randomization analysis but also the choice of valid instruments. To be a valid instrumental variable, a SNP should be associated only with the respective risk/protective factor. For the correlated traits HDL-C, LDL-C and TG, there is substantial overlap of the SNPs, which makes it extremely difficult to discriminate the instrumental variables for each of the lipid traits correctly from each other. The exclusion of all instrumental variables that were found to be associated with at least two of the three lipid traits would lead to a substantial loss of strength for the Mendelian randomization analyses. However, we checked for possible pleiotropy of the SNPs using appropriate statistical methods and adjusted for the effects of the other lipid traits to find independent causal effects. Still, the interpretation of the identified causal effects remains difficult.

Another limitation which arises from using summary-level data is that observed effect sizes cannot be derived from directly and thus, if not obtainable from the literature, cannot be compared with the estimated causal effects. Although we only investigated causal relationships for which there is already sufficient evidence of either observational association or functional experiments, direct comparison of observational and causal estimate is only possible if it is on the same scale. All GWAS meta-analysis data we used for our analyses are derived from either log-transformation or inverse normal transformation. However, such transformations are hardly ever done in observational epidemiological studies. Results from functional wet-lab experiments can certainly not be transferred into observational estimates.

To summarize, our data revealed an inverse causal association of kidney function (eGFR) on apoA-IV. Furthermore, investigating the lipid traits suggested a causal involvement of primarily triglyceride levels on apoA-IV concentrations. The causal effects of HDL-C and LDL-C on apoA-IV are hard to discriminate from each other and from the effects triggered by TG. Our results also argue for a causal, inverse association of apoA-IV concentrations on HDL-C, which however have to be interpreted with caution due to potential presence of pleiotropic effects of the genetic markers.

## Methods

### Data sources and SNP selection

For all evaluated disease-related traits and apoA-IV, SNPs were selected and summarized results were taken from the most recent and most comprehensive GWAS, which were conducted primarily in cohorts of European ancestry. A graphical overview of the design and studies used in our investigation is given in Fig. 1. A fraction of the five studies which have been included in the apoA-IV-GWAS meta-analysis have also been part of the GWAS meta-analyses of the other parameters (lipids, eGFR, adiposity-related parameters and fasting glucose). The overlap of samples ranges between 19% and 80%. Details on the data sources and SNP selection can be found in the Supplementary material.

### Measurement of apoA-IV

For all participating studies, quantification of plasma apoA-IV was done in the same laboratory (Division of Genetic Epidemiology, Medical University of Innsbruck, Austria). It was based on a double-antibody enzyme-linked immunosorbent assay using an affinity-purified polyclonal rabbit anti-human apoA-IV antibody for coating and the same antibody coupled to horseradish peroxidase for detection. Plasma with a known concentration of apoA-IV was used as the calibration standard^45^. Four control sera with different concentrations were run on each plate in double measurements for control purposes throughout the entire project. The intra- and interassay coefficients of variation were 2.7% and 6.0%, respectively^45^.

## Statistical Methods

### Validity of instrumental variables and power analysis

In Mendelian randomization analyses, instrumental variables should meet certain requirements to minimize weak instrument bias.

The strength of the instrumental variables (SNPs) used for the Mendelian randomization analysis was assessed using the explained variance (R²). R² was calculated according to Pattaro *et al*.^33^. Using this formula, the percentage of phenotypic variance explained by the instrumental variables (SNPs) can be estimated as ${R}^{{\rm{2}}}={\sum }_{i=1}^{k}{R}_{i}^{2}$, where ${R}_{i}^{2}={\beta }_{i}^{2}{var}({SN}{P}_{i})/{var}(X)$ is the coefficient of determination for all $k$ SNPs associated with the potential risk factor/exposure $X$, ${\beta }_{i}$ is the estimated effect of the i^th^ SNP on the risk factor $X$, ${var}({SN}{P}_{i})\,=\,2\times {MA}{F}_{{SN}{P}_{i}}\times (1-{MA}{F}_{{SN}{P}_{i}})$ and ${var}(X)$ is the variance of the potential risk factor $X$ (${var}(X)\,=\,1$ for the lipid and adiposity traits, since the beta estimates refer to change in 1 standard deviation (SD)).

The F-statistic is typically used to judge on the validity of instrumental variables. However, it cannot be calculated in this setting, where only summary-level data are available. However, only genome-wide significant SNPs that are independent from each other (pairwise LD between all SNPs: r^2^ < 0.1) were included in this analysis (p-value < 5 × 10^−8^). This corresponds to an F statistic > 30 for each single variant^46^. In the Mendelian randomization literature a threshold of F < 10 has typically been used to define a “weak IV” (the Staiger-Stock rule^47, 48^). Since we are using a combination of several genome-wide significant SNPs, weak instrument bias is negligble.

Power calculations were carried out using the online tool https://sb452.shinyapps.io/power/. The power cannot be calculated directly as different units were used for the different traits and only summary-level data are available. Therefore, a rough approximation for all investigated traits was performed based on standardized values. The applied online tool was rather meant for Mendelian randomization methods based on individual-level data. However, as shown in ref. 49, analyses based on individual level data and summarized data methods are comparable with respect to power.

The sample size assumed for the power analysis is set to the sample size of the outcome dataset, since the ratio estimate involves the variance of the outcome dataset, but not the variance of the exposure dataset. Therefore, the sample size was set to 13,800 for apoA-IV as the outcome variable. For the power-analysis of the reverse causation (apoA-IV as exposure on the various phenotypes), a sample size of 188,577 is assumed for the lipid analysis etc. (Table 2). Given ${{R}}^{2}$ of the genetic variants on the exposure, the sample size, the desired power (80%) and the significance level, it can be calculated which causal effect estimate can be detected with the given data, if it is truly there. This detectable causal estimate ${\beta }_{{causal}}$ is based on change in one SD of the outcome per one SD change in exposure. In this setting ${\beta }_{{causal}}^{2}$ is equal to the variance explained (${R}_{{causal}}^{2}$) of the exposure variable on the outcome. Therefore, we can answer the question, which strength of causal association we can most likely detect with the given data.

### Mendelian randomization methods

The Mendelian randomization analyses were performed bidirectional. In the first run, apoA-IV was considered as an outcome variable whereas lipids, eGFR, BMI, WHR (adjusted for BMI) and fasting glucose levels were considered as exposures. In the second run, apoA-IV was the exposure variable and the other phenotypes (lipids, eGFR, BMI, WHR (adjusted for BMI) and fasting glucose levels) were used as outcomes.

Before any analysis, the SNP-exposure and SNP-outcome association estimates have all been oriented towards an increase in the exposure trait.

For the main Mendelian randomization analysis, the SNP-exposure and SNP-outcome estimates were combined using the inverse-variance weighted (MR-IVW) method as proposed by Burgess *et al*.^46^. Causal estimates based on this method are notated as β_MR-IVW_. The MR-IVW estimate can also be interpreted as a weighted regression from the effect estimates of the exposure SNPs on the estimates of the outcome of the same SNPs (removing the intercept).

As the MR-IVW method assumes that all genetic variants satisfy the IV assumptions (including no pleiotropy of all included SNPs, and No Measurement Error (NOME) in the gene-exposure association estimates), sensitivity analyses were performed, where different methods were used to detect possible pleiotropy and also to account for it:

1. The MR-Egger regression: assesses directional pleiotropy by borrowing from the same principles of testing for small study bias in meta-analysis (causal estimate notated as β_MR-Egger_).
2. The weighted median estimation method: Allows that up to 50% of the weight of genetic markers under analysis comes from invalid instruments and retains more power than MR-Egger.
3. The exclusion of possible pleiotropic SNPs based on the gtx package in R (causal estimate notated as β_MR-IVW_gtx_).
4. The exclusion of possible pleiotropic SNPs using a proxy search.
5. For the lipids: the multivariable Mendelian randomization adjusting for the effect estimates of the other lipid phenotypes (causal estimate notated as β_adjusted_).

MR-Egger regression^50^ was used to investigate whether there is directional bias caused by pleiotropy. Directional bias means that the pleiotropic effects of genetic variants are not balanced about the null and are drawn into one direction. This regression is an adaption of the standard Egger regression which is used to analyze small study bias in the meta-analysis literature. The intercept obtained from the MR-Egger regression gives an estimate of directional bias and the slope coefficient provides an estimate of the causal effect, which is consistent even when all the genetic variants are invalid instrumental variables with respect to pleiotropy^50^. To assess whether the MR-Egger regression estimates might be biased through a violation of the NOME assumption, an adaption of the I² statistic $({{I}^{2}}_{{\rm{G}}{\rm{X}}})$ ^51^ was calculated and the corrected MR-Egger estimate was computed using the method of Simulation Extrapolation (SIMEX)^51^.

Additionally to the MR-Egger regression, the weighted median estimator was calculated as proposed by Bowden *et al*.^52^. In this method, the ratio estimates of the SNP-exposure and SNP-outcome association estimates are ordered and weighted by the inverse of their variance. The weighted median estimator is then the median of these estimates, according to the weights. This estimator is consistent if at least 50% of the weight comes from valid instrumental variables. Although the MR-Egger regression method allows all the instrumental variables to be invalid, the weighted median estimation approach offers the advantages of an improved precision compared to the MR-Egger regression. Therefore, both methods were used to assess whether pleiotropy had influenced our results.

In two further sensitivity analyses, all SNPs that were assumed to have pleiotropic effects were excluded. In Mendelian randomization analyses, bias due to pleiotropy only occurs when the SNPs are associated with other phenotypes, which also influence the outcome variable or are independently associated with the outcome variable itself. If this is not the case and there is also no direct effect of the SNPs on the outcome variable, the effect of the SNPs on the outcome is mediated completely by the intermediate variable. Then, the causal effects of all SNPs individually should rather be homogeneous and approximate the true unknown causal effect of the exposure variable on the outcome^32, 53, 54^. This assumption was tested by a goodness of fit test using the function “grs.filter.Qrs” in package “gtx” in R (Johnson, T.: Efficient Calculation for Multi-SNP Genetic Risk Scores. Poster presentation at the American Society of Human Genetics Annual Meeting, San Francisco, 2012). This function performs a stepwise downward model selection in which SNPs are iteratively removed from the risk score until the heterogeneity test is no longer significant at the specified threshold (p_threshold_ = 0.05). SNPs showing a deviation from this assumption are therefore potentially not mediated completely by the exposure and were excluded in a further sensitivity analysis. This was only the case, however, for one HDL-C SNP. In a second approach used to account for possible pleiotropy, we looked up whether there are either any overlapping SNPs in the different SNP selection datasets or SNPs in LD with each other. To find the proxies for each SNP, we used SNiPA^55^ with the 1000 Genomes Phase 3 v5 variant set and an LD-threshold of r² > 0.8. If overlaps were found, these SNPs were excluded. For the lipids, we followed two different approaches. First, we excluded all SNPs that were found in any other SNP selection dataset, no matter which trait. Second, we excluded only those SNPs that were found in other non-lipid SNP selection datasets (i.e. in the SNP selection for eGFR, BMI, WHR, FG and apoA-IV). In this case, an adjustment for the other lipid traits using multivariable Mendelian randomization was included as described below, to adjust for the lipid traits as well. This second approach was performed due to the huge SNP overlap between the different lipid traits.

For the lipid traits, we also performed a multivariable Mendelian randomization adjusting each lipid-apoA-IV causal estimate by the estimates of the SNP-lipids association not under consideration (i.e., when assessing the causal association between HDL-C and apoA-IV adjusting for LDL-C and TG associations with the same SNPs). For this, an extension to the inverse-variance weighted method was used, as proposed by Burgess *et al*.^56, 57^. In this approach the gene-outcome associations are regressed on all the gene-risk factor associations simultaneously in a multivariable regression model, using the SNPs associated with the risk factor of interest.

In the case where apoA-IV acted as the exposure variable, only two (for the lipids, eGFR, BMI and WHR) or three (for fasting glucose) SNPs were available for the MR analysis. Here, all four sensitivity analyses were not applicable or meaningful and therefore only the MR-IVW method was applied.

As four different “trait blocks” including correlated traits were analyzed, a p-value of 0.05/8 = 0.00625 was considered as significant.

LD structures and pairwise SNP correlations were retrieved from SNiPA (http://snipa.helmholtz-muenchen.de/)^55^ using 1000 Genomes phase 3 data and superimposed to UCSC Genome Browser hg19 using modified scripts from^58^. Expression QTL data for rs964184 and for all genes in the *APOA5-A4-C3-A1* (*APOA5, APOA4, APOC3, APOA1, ZRP1/ZNF259, BUD13*) cluster was retrieved from GTEx (www.gtexportal.org)^59^.

## Electronic supplementary material

Supplementary text, tables and figures
