# Supplementary material for: Evaluating the Causal Relation of ApoA-IV with Disease-Related Traits - A Bidirectional Two-sample Mendelian Randomization Study
Source: Sci Rep. 2017 Aug 18;7:8734. doi: 10.1038/s41598-017-07213-9 (PMC5562707; doi:10.1038/s41598-017-07213-9)
Supplement: Supplementary file 1 — Supplementary text, tables and figures [file 41598_2017_7213_MOESM1_ESM.pdf]

SUPPLEMENTAL MATERIAL to

Evaluating the Causal Relation of ApoA-IV with Disease-Related Traits - A Bidirectional Two-sample Mendelian Randomization Study

Salome Mack[1], Stefan Coassin[1], Julien Vaucher[2], Florian Kronenberg[1], ApoA-IV-GWAS Consortium, Claudia Lamina[1]*

[1] Division of Genetic Epidemiology, Department of Medical Genetics, Molecular and Clinical Pharmacology, Medical University of Innsbruck, Innsbruck, Austria

[2] Department of Internal Medicine, Lausanne University Hospital, Lausanne, Switzerland

***Address of correspondence:**
Claudia Lamina, PhD
Division of Genetic Epidemiology
Department of Medical Genetics, Molecular and Clinical Pharmacology
Medical University of Innsbruck
Schöpfstr. 41, A-6020 Innsbruck, AUSTRIA
Phone: (+43) 512 9003-70565
Fax: (+43) 512 9003-73560 or -73561
E-mail: Claudia.Lamina@i-med.ac.at

Content

Supplementary Text 3
Supplementary Tables 8
Supplementary Figures 29

Supplementary Text

Methods: Data sources and SNP selection

Lipid traits

We used publicly available data from the Global Lipids Genetics Consortium (GLGC; http://csg.sph.umich.edu/abecasis/public/lipids2013/) to select genetic variants, which were genome-wide significantly associated ($p<5x10^{-8}$) with lipid traits (HDL cholesterol [HDL-C], LDL cholesterol [LDL-C] and triglycerides [TG])[1] . The GLGC meta-analyzed data from 23 studies of European ancestry (n=94,595 individuals), using inverse variance-weighted fixed effect models. 196,710 genetic variants were genotyped with genome-wide SNP arrays and additionally 37 studies (n=93,982 individuals) where 5,023 SNPs for HDL-C, 5,055 SNPs for LDL-C, and 5,056 SNPs for TG were genotyped with the Metabochip array [2]. SNPs were imputed using HapMap release 22. When there were multiple SNPs within one locus having a p-value$<5x10^{-8}$, we selected the leading SNP based on the lowest p-value. Hence, we found 71 independent SNPs for HDL-C (sample size ranging from 92,820 to 187,141), 58 independent SNPs for LDL-C (sample size ranging from 80,502 to 173,045) and 40 independent SNPs for TG (sample size ranging from 83,616 to 177,825). All datasets were adjusted for age, age^2 and sex. Each dataset included the SNP rs964184, which is located in the *APOA5-A4-C3-A1* gene cluster and is in close proximity to the *APOA4* gene. Since that SNP may directly influence apoA-IV concentrations itself, which would violate a Mendelian randomization assumption, we excluded this SNP in sensitivity analyses.

For all SNPs beta effect estimates, standard errors and p-values were retrieved from the original publication. Beta effect estimates are expressed in standard deviations of inverse-normally transformed values for each increase in number of the effect allele. The effect allele was defined as the allele oriented towards an increase in the investigated lipid trait.

Kidney function

We used publicly available data from the CKDGen Consortium (https://www.nhlbi.nih.gov/research/intramural/researchers/pi/fox-caroline/datasets) to select genetic variants, which were genome-wide significantly associated ($p<5x10^{-8}$) with estimated glomerular filtration rate (eGFR) based on serum creatinine [3]. This consortium combined genome-wide data from up to 133,413 individuals of European ancestry from 49 predominantly population-based studies in ≈2.5 million SNPs using inverse variance-weighted fixed effect models. In 45 studies, SNPs were imputed using HapMap II CEU and 4 studies were imputed using the 1000 Genomes

Project data. For each locus that was found to be associated with the log-transformed eGFR values with SNPs having a p-value $<5x10^{-8}$, the lead SNP was selected, resulting in a list of 53 independent SNPs (sample size ranging from 115,895 to 133,810). For the single-SNP-analysis, the dataset was adjusted for age and sex. For all SNPs, beta effect estimates, standard errors and p-values were retrieved from the original publication. Beta effect estimates refer to change in log-transformed eGFR for each increase in number of the effect allele. The effect allele was defined as the allele oriented towards an increase in eGFR.

Adiposity

We used data from the Genetic Investigation of ANthropometric Traits (GIANT) Consortium (http://portals.broadinstitute.org/collaboration/giant/index.php/GIANT_consortium_data_files) to select genetic variants, which were genome-wide significantly associated ($p<5x10^{-8}$) with adiposity-related traits (BMI and WHR adjusted for BMI [4,5]. The dataset used for the investigation of BMI comprised 114 studies including 322,206 individuals of European ancestry and over 2.5 million SNPs genotyped using the Metabochip array [2]. SNPs were imputed using HapMap phase II CEU. When there were multiple SNPs within one locus having a p-value$<5x10^{-8}$, we selected the leading SNP based on the lowest p-value, resulting in 77 independent SNPs (sample size ranging from 205,675 to 322,135). For the single-SNP-analysis, the dataset was adjusted for age, age^2 and sex. The 20 SNPs that were found in non-Europeans only were not included in this MR analysis since the dataset from the apoA-IV GWAS included only European populations.

The dataset used for the investigation of WHR, which was adjusted for BMI (WHRadjBMI), combined 57 cohorts including 142,762 individuals of European ancestry that were genotyped with genome-wide SNP arrays and additionally 44 cohorts including 67,326 individuals of European ancestry that were genotyped with the Metabochip array [2]. Genotypes were imputed using CEU haplotypes from HapMap resulting in ≈2.5 million SNPs. When there were multiple SNPs within one locus having a p-value$<5x10^{-8}$, we selected the leading SNP based on the lowest p-value, resulting in 40 independent SNPs (sample size ranging from 191,245 to 231,293). For this analysis sex-specific residuals were calculated and adjusted for age, age^2 and BMI. The 8 sex-specific SNPs as well as one SNP found in non-Europeans only were not included in this MR analysis.

For both adiposity-related traits, the beta effect estimates refer to one standard deviation of inverse-normally transformed phenotypes for each increase in number of the effect allele. For all SNPs, beta effect estimates, standard errors and p-values were retrieved from the original publication.

Glucose

We used data from Scott et al from the Meta-Analyses of Glucose and Insulin-related traits Consortium (MAGIC; www.magicinvestigators.org/) to select genetic variants, which were genome-wide significantly associated ($p<5x10^{-8}$) associated with fasting glucose [6]. This dataset combined genome-wide data from up to 133,010 individuals of European ancestry from 65 population-based studies including SNPs genotyped using the Metabochip array [2]. Single study data were meta-analyzed using inverse variance-weighted fixed effect models. Studies with genome-wide arrays imputed genotypes using the HapMap CEU reference panel. From a total of ≈2.5 million directly genotyped or imputed SNPs, study-specific results for ≈66,000 Metabochip follow-up SNPs were considered for the meta-analyses. When there were multiple SNPs within one locus having a p-value<$5x10^{-8}$, we selected the leading SNP based on the lowest p-value, resulting in 36 independent SNPs (sample size ranging from 106,897 to 132,996). All analyses were adjusted for age, study site and geographical covariates. For all SNPs beta effect estimates, standard errors and p-values were retrieved from the original publication. Beta effect estimates refer to change in mmol/L in fasting glucose levels for each increase in number of the effect allele. The effect allele was defined as the allele oriented towards an increase in fasting glucose.

None of the three apoA-IV-associated SNPs (see next paragraph) was available in the dataset corresponding to the analysis by Scott et al. Therefore, we used data by Manning et al. from the MAGIC consortium to retrieve these apoA-IV-associated SNPs [7]. This dataset combined genome-wide data from up to 58,070 individuals of European ancestry from 29 studies in the first phase of discovery analysis and 38,422 individuals of European ancestry from 23 cohorts in the second phase of analysis. SNPs in the first phase were genotyped using genome-wide SNP arrays. Imputation was performed on the basis of the HapMap Utah residents of Northern and Western European ancestry (CEU) population (Build 36), resulting in ≈2.4 million SNPs that were included in the discovery phase meta-analyses. All single-SNP-analyses were adjusted for age, sex and study-level covariates (see original publication for details). For the follow-up genotyping, studies were invited to participate through either in silico searches with existing genotype data or de novo genotyping. A large percentage of the follow-up cohorts used the Illumina Cardio-Metabochip. For all 3 SNPs found to be associated with apoA-IV, beta effect estimates, standard errors and p-values were retrieved from the original publication. Beta effect estimates refer to log-transformed values of fasting glucose levels for each increase in number of the effect allele.

ApoA-IV

SNPs associated with lipids, kidney function, adiposity and glucose level (see above) were directly matched with those available from the currently largest genome-wide association studies meta-analysis on apoA-IV [8]. This meta-analysis comprised data of 5 studies with 13,813 individuals of

European ancestry in the discovery stage as well as 2 further studies with 2,267 individuals of European ancestry in the replication stage (altogether n=16,080 for replicated SNPs). In all studies, SNPs were imputed using 1000 Genomes (phase 1, version 3) resulting in >7 million SNPs available for meta-analysis. The quantification of plasma apoA-IV was done in one laboratory (Division of Genetic Epidemiology, Medical University of Innsbruck, Austria) for all 5 GWA studies participating in the meta-analysis. It was based on a double-antibody enzyme-linked immunosorbent assay using an affinity-purified polyclonal rabbit anti-human apoA-IV antibody for coating and the same antibody coupled to horseradish peroxidase for detection. Plasma with a known concentration of apoA-IV was used as the calibration standard [9]. Four control sera with different concentrations were run on each plate in double measurements for control purposes throughout the entire project. The intra- and interassay coefficients of variation were 2.7% and 6.0%, respectively [9]. The apoA-IV concentration values were log-transformed due to their skewed distribution. For the genome-wide SNP-analysis, all datasets were adjusted for age and sex. Beta effect estimates refer to log-transformed apoA-IV concentrations on the original scale(mg/dL).

To investigate the inverse association , namely whether apoA-IV concentration is causally associated with lipid traits, kidney function, adiposity and fasting glucose , we retrieved all SNPs passing a genome-wide threshold (p-value $<5x10^{-8}$) within each locus that was associated with the log-transformed apoA-IV concentrations . When there were multiple SNPs within one locus having a p-value$<5x10^{-8}$, we selected the leading SNP based on the lowest p-value. Hence, we found three independent SNPs ($r^2<0.1$), two in the *APOA4* gene region (rs1729407 with an F-statistic of 222 and rs5104 with an F-statistic of 161) and one in the *KLKB1* gene region (rs4241819 with an F-statistic of 71).

References

1. Global Lipids Genetics Consortium *et al.* Discovery and refinement of loci associated with lipid levels. *Nat. Genet.* **45,** 1274–83 (2013).
2. Voight, B. F. *et al.* The metabochip, a custom genotyping array for genetic studies of metabolic, cardiovascular, and anthropometric traits. *PLoS Genet.* **8,** e1002793 (2012).
3. Pattaro, C. *et al.* Genetic associations at 53 loci highlight cell types and biological pathways relevant for kidney function. *Nat. Commun.* **7,** 10023 (2016).
4. Locke, A. E. *et al.* Genetic studies of body mass index yield new insights for obesity biology. *Nature* **518,** 197–206 (2015).

5. Shungin, D. *et al.* New genetic loci link adipose and insulin biology to body fat distribution. *Nature* **518,** 187–96 (2015).

6. Scott, R. A. *et al.* Large-scale association analyses identify new loci influencing glycemic traits and provide insight into the underlying biological pathways. *Nat. Genet.* **44,** 991–1005 (2012).

7. Manning, A. K. *et al.* A genome-wide approach accounting for body mass index identifies genetic variants influencing fasting glycemic traits and insulin resistance. *Nat. Genet.* **44,** 659–69 (2012).

8. Lamina, C. *et al.* A genome-wide association meta-analysis on apolipoprotein A-IV concentrations. *Hum. Mol. Genet.* **25,** 3635–3646 (2016).

9. Kronenberg, F., Lobentanz, E. M., König, P., Utermann, G. & Dieplinger, H. Effect of sample storage on the measurement of lipoprotein[a], apolipoproteins B and A-IV, total and high density lipoprotein cholesterol and triglycerides. *J. Lipid Res.* **35,** 1318–28 (1994).

Supplementary Tables

Supplementary Table 1: Characteristics and results of the 71 SNPs associated with HDL-C [1,8]. The table is sorted by the p-value for HDL-C.

						Results from the GWAS meta-analysis on					
						HDL-C			ApoA-IV		
SNP	Chr	Pos	Nearest gene	A1	A2	Beta*	SE*	P-value	Beta§	SE§	P-value
rs3764261	16	56993324	*CETP*	A	C	0.2412	0.0039	1.00E-769	0.0043	0.0043	0.3175
rs1532085	15	58683366	*LIPC*	A	G	0.1068	0.0035	1.24E-188	0.0050	0.0038	0.1834
rs12678919	8	19844222	*LPL*	G	A	0.1554	0.0057	1.38E-149	0.0095	0.0059	0.1053
rs1883025	9	107664301	*ABCA1*	C	T	0.0698	0.0041	1.50E-65	0.0010	0.0042	0.8095
rs16942887	16	67928042	*PSKH1*	A	G	0.0831	0.0051	8.28E-54	0.0164	0.0054	0.0022
rs964184	11	116648917	*ZPR1*	C	G	0.1065	0.0071	6.09E-48	0.0198	0.0052	0.0001
rs7241918	18	47160953	*LIPG*	T	G	0.0902	0.0064	1.11E-44	0.0127	0.0050	0.0114
rs9987289	8	9183358	*PPP1R3B*	G	A	0.0817	0.0062	1.95E-41	-0.0017	0.0066	0.8036
rs4846914	1	230295691	*GALNT2*	A	G	0.0479	0.0034	3.51E-41	0.0021	0.0037	0.5637
rs6065906	20	44554015	*PCIF1*	T	C	0.0594	0.0044	5.34E-40	-0.0040	0.0048	0.4024
rs1800961	20	43042364	*HNF4A*	C	T	0.1270	0.0099	1.64E-34	-0.0059	0.0136	0.6627
rs838880	12	125261593	*SCARB1*	C	T	0.0484	0.0039	6.38E-32	0.0073	0.0051	0.1584
rs2954029	8	126490972	*TRIB1*	T	A	0.0401	0.0034	2.67E-29	0.0036	0.0036	0.3220
rs3136441	11	46743247	*F2*	C	T	0.0545	0.0047	6.76E-29	0.0029	0.0056	0.6095
rs174546	11	61569830	*FADS1*	C	T	0.0391	0.0035	8.30E-28	0.0028	0.0039	0.4712
rs386000	19	54792761	*LILRA3*	C	G	0.0479	0.0047	2.71E-23	-0.0022	0.0057	0.6950
rs4420638	19	45422946	*APOC1*	A	G	0.0669	0.0068	1.72E-21	0.0083	0.0061	0.1780
rs1689800	1	182168885	*ZNF648*	A	G	0.0344	0.0036	4.77E-20	0.0019	0.0037	0.6055
rs581080	9	15305378	*TTC39B*	C	G	0.0419	0.0045	1.04E-19	-0.0017	0.0048	0.7231
rs2925979	16	81534790	*GAN*	C	T	0.0351	0.0037	1.32E-19	-0.0049	0.0041	0.2277
rs4660293	1	40028180	*PABPC4*	A	G	0.0353	0.004	2.86E-18	-0.0017	0.0043	0.6827
rs113359481	22	21932068	*RIMBP3C*	C	T	0.0379	0.0042	4.30E-18	0.0029	0.0046	0.5324
rs2972146	2	227100698	*IRS1*	G	T	0.0323	0.0035	1.85E-17	-0.0027	0.0038	0.4814
rs11869286	17	37813856	*STARD3*	C	G	0.0319	0.0037	2.70E-17	0.0073	0.0038	0.0560
rs2293889	8	116599199	*TRPS1*	G	T	0.0312	0.0035	4.27E-17	0.0047	0.0037	0.1976
rs4731702	7	130433384	*KLF14*	T	C	0.0294	0.0034	4.84E-17	-0.0071	0.0037	0.0521
rs12748152	1	27138393	*PIGV*	C	T	0.0506	0.0062	9.74E-16	0.0092	0.0076	0.2281
rs13107325	4	103188709	*SLC39A8*	C	T	0.0708	0.0078	1.07E-15	0.0014	0.0072	0.8452
rs12328675	2	165540800	*COBLL1*	C	T	0.0447	0.0052	2.13E-15	0.0020	0.0055	0.7230
rs112302432	11	122522375	*UBASH3B*	C	T	0.0269	0.0035	1.12E-14	-0.0025	0.0037	0.4970
rs7134594	12	110000193	*MMAB*	T	C	0.0354	0.0048	1.77E-13	0.0018	0.0036	0.6132
rs11613352	12	57792580	*R3HDM2*	T	C	0.0281	0.004	2.39E-13	-0.0009	0.0044	0.8307
rs17695224	19	52324216	*FPR3*	G	A	0.0290	0.0039	2.42E-13	-0.0001	0.004	0.9841
rs17145738	7	72982874	*TBL2*	T	C	0.0408	0.0053	4.95E-13	0.0071	0.0057	0.2152
rs4148008	17	66875294	*ABCA8*	C	G	0.0280	0.0038	1.13E-12	0.0074	0.0039	0.0594

to be continued…

Supplementary Table 1 continued

						Results from the GWAS meta-analysis on					
						HDL-C			ApoA-IV		
SNP	Chr	Pos	Nearest gene	A1	A2	Beta*	SE*	P-value	Beta§	SE§	P-value
rs3822072	4	89741269	*FAM13A*	G	A	0.0251	0.0034	4.06E-12	-0.0021	0.0037	0.5749
rs702485	7	6449272	*DAGLB*	G	A	0.0243	0.0034	6.45E-12	0.0036	0.0037	0.3231
rs2013208	3	50129399	*SEMA3F*	T	C	0.0254	0.0036	8.92E-12	0.0032	0.0036	0.3723
rs4142995	7	17919258	*SNX13*	G	T	0.0263	0.0037	9.37E-12	-0.0080	0.0037	0.0299
rs4129767	17	76403984	*PGS1*	A	G	0.0237	0.0034	2.10E-11	-0.0027	0.0036	0.4546
rs998584	6	43757896	*VEGFA*	C	A	0.0260	0.0038	2.27E-11	0.0022	0.0042	0.5968
rs2652834	15	63396867	*LACTB*	G	A	0.0285	0.0043	3.59E-11	-0.0024	0.0044	0.5868
rs13326165	3	52532118	*STAB1*	A	G	0.0289	0.0043	9.04E-11	-0.0067	0.0048	0.1623
rs11246602	11	51512090	*OR4C46*	C	T	0.0340	0.0052	1.68E-10	0.0036	0.0062	0.5641
rs970548	10	46013277	*MARCH8*	C	A	0.0258	0.0039	1.71E-10	0.0005	0.0041	0.9003
rs1936800	6	127436064	*RSPO3*	C	T	0.0200	0.0034	3.06E-10	-0.0031	0.0036	0.3956
rs6450176	5	53298025	*ARL15*	G	A	0.0254	0.0039	6.88E-10	0.0019	0.0041	0.6483
rs4765127	12	124460167	*ZNF664*	T	G	0.0324	0.005	7.79E-10	-0.0076	0.0037	0.0424
rs1047891	2	211540507	*CPS1*	C	A	0.0269	0.0039	8.73E-10	-0.0010	0.0041	0.7985
rs731839	19	33899065	*PEPD*	A	G	0.0220	0.0037	3.44E-09	0.0019	0.0039	0.6304
rs2290547	3	47061183	*SETD2*	G	A	0.0297	0.0046	3.69E-09	0.0145	0.0059	0.0144
rs13076253	3	131751775	*CPNE4*	A	C	0.0283	0.0048	4.96E-09	-0.0047	0.0052	0.3749
rs13076253	3	131751775	*CPNE4*	A	C	0.0283	0.0048	4.96E-09	-0.0047	0.0052	0.3749
rs4650994	1	178515312	*C1orf220*	G	A	0.0210	0.0034	6.70E-09	-0.0102	0.0036	0.0046
rs1121980	16	53809247	*FTO*	G	A	0.0196	0.0034	6.79E-09	0.0041	0.0036	0.2583
rs4983559	14	105277209	*ZBTB42*	G	A	0.0197	0.0036	9.57E-09	0.0060	0.0044	0.8870
rs4917014	7	50305863	*IKZF1*	G	T	0.0222	0.0036	1.03E-08	-0.0069	0.0039	0.0758
rs7134375	12	20473758	*PDE3A*	A	C	0.0207	0.0035	1.05E-08	-0.0004	0.0039	0.9112
rs499974	11	75455021	*MOGAT2*	C	A	0.0263	0.0044	1.12E-08	0.0011	0.0047	0.8164
rs6805251	3	119560606	*GSK3B*	T	C	0.0200	0.0035	1.33E-08	-0.0022	0.0037	0.5399
rs12145743	1	156700651	*C1orf66*	G	T	0.0203	0.0036	1.80E-08	-0.0038	0.004	0.3399
rs7255436	19	8433196	*ANGPTL4*	A	C	0.0316	0.0053	1.87E-08	-0.0071	0.0046	0.1258
rs17173637	7	150529449	*ABP1*	T	C	0.0363	0.0057	1.90E-08	0.0075	0.0062	0.2311
rs605066	6	139829666	*CITED2*	T	C	0.0281	0.0049	2.79E-08	0.0037	0.0037	0.3127
rs4759375	12	123796238	*SBNO1*	T	C	0.0560	0.0102	3.01E-08	0.0027	0.0062	0.6621
rs12801636	11	65391317	*PCNXL3*	A	G	0.0235	0.0042	3.15E-08	0.0038	0.0047	0.4104
rs12967135	18	57849023	*MC4R*	G	A	0.0262	0.0045	3.57E-08	-0.0053	0.0042	0.2071
rs2606736	3	11400249	*ATG7*	C	T	0.0246	0.0043	4.80E-08	0.0007	0.0037	0.8523
rs10019888	4	26062990	*RBPJ*	A	G	0.0270	0.0046	4.90E-08	-0.0031	0.0048	0.5123
rs2602836	4	100014805	*ADH5*	A	G	0.0192	0.0034	4.96E-08	-0.0013	0.0037	0.7217
rs2923084	11	10388782	*ADM*	A	G	0.0256	0.0045	5.02E-08	0.0063	0.0048	0.1907

A1: effect allele; A2: other allele.

*units in SD; §units in log(apoA-IV).

Supplementary Table 2: Characteristics and results of the 58 SNPs associated with LDL-C [1,8]. The table is sorted by the p-value for LDL-C.

						Results from the GWAS meta-analysis on					
						LDL-C			ApoA-IV		
**SNP**	**Chr**	**Pos**	**Nearest gene**	**A1**	**A2**	**Beta***	**SE***	**P-value**	**Beta§**	**SE§**	**P-value**
rs6511720	19	11202306	*LDLR*	G	T	0.2209	0.0061	3.85E-262	-0.0060	0.0070	0.3900
rs629301	1	109818306	*CELSR2*	T	G	0.1669	0.0049	5.40E-241	-0.0102	0.0044	0.0192
rs1367117	2	21263900	*APOB*	A	G	0.1186	0.0040	9.48E-183	0.0013	0.0039	0.7356
rs4420638	19	45422946	*APOC1*	G	A	0.2251	0.0077	1.51E-178	-0.0083	0.0061	0.1780
rs12916	5	74656539	*HMGCR*	C	T	0.0733	0.0038	7.79E-78	-0.0055	0.0038	0.1423
rs4299376	2	44072576	*ABCG8*	G	T	0.0812	0.0045	3.94E-72	-0.0046	0.0042	0.2746
rs10401969	19	19407718	*SF4*	T	C	0.1184	0.0072	2.65E-54	-0.0107	0.0071	0.1351
rs2954029	8	126490972	*TRIB1*	A	T	0.0564	0.0036	2.10E-50	-0.0036	0.0036	0.3220
rs2479409	1	55504650	*PCSK9*	G	A	0.0642	0.0041	2.52E-50	-0.0034	0.0041	0.3978
rs635634	9	136155000	*ABO*	T	C	0.0772	0.0055	1.83E-41	-0.0060	0.0047	0.2010
rs2000999	16	72108093	*HPR*	A	G	0.0650	0.0046	4.22E-41	0.0030	0.0046	0.5177
rs174546	11	61569830	*FADS1*	C	T	0.0512	0.0038	1.63E-39	0.0028	0.0039	0.4712
rs3764261	16	56993324	*CETP*	C	A	0.0528	0.0042	2.22E-34	-0.0043	0.0043	0.3175
rs2131925	1	63025942	*DOCK7*	T	G	0.0489	0.0039	2.98E-32	-0.0026	0.0039	0.4977
rs6882076	5	156390297	*TIMD4*	C	T	0.0456	0.0038	3.31E-31	0.0009	0.0037	0.8102
rs964184	11	116648917	*ZPR1*	G	C	0.0855	0.0078	2.01E-26	-0.0198	0.0052	0.0001
rs9987289	8	9183358	*PPP1R3B*	G	A	0.0714	0.0066	8.53E-24	-0.0017	0.0066	0.8036
rs1564348	6	160578860	*SLC22A1*	C	T	0.0481	0.0050	2.76E-21	-0.0009	0.0051	0.8666
rs1169288	12	121416650	*HNF1A*	C	A	0.0375	0.0040	6.45E-21	0.0034	0.0040	0.3915
rs11220462	11	126243952	*ST3GAL4*	A	G	0.0590	0.0059	6.61E-21	0.0041	0.0054	0.4493
rs6029526	20	39672618	*TOP1*	A	T	0.0436	0.0052	4.81E-18	-0.0067	0.0036	0.0676
rs3757354	6	16127407	*MYLIP*	C	T	0.0382	0.0044	2.09E-17	-0.0053	0.0045	0.2464
rs3177928	6	32412435	*HLA-DRB5*	A	G	0.0452	0.0052	3.10E-17	0.0025	0.0074	0.7396
rs2072183	7	44579180	*NPC1L1*	C	G	0.0386	0.0047	7.12E-16	0.0044	0.0058	0.4475
rs8017377	14	24883887	*NYNRIN*	A	G	0.0303	0.0038	2.52E-15	-0.0041	0.0041	0.3241
rs12027135	1	25775733	*TMEM57*	T	A	0.0300	0.0038	2.27E-14	0.0028	0.0039	0.4757
rs4722551	7	25991826	*MIR148A*	C	T	0.0391	0.0049	3.95E-14	0.0058	0.0051	0.2603
rs12670798	7	21607352	*DNAH11*	C	T	0.0344	0.0043	4.81E-14	-0.0017	0.0045	0.7081
rs1800562	6	26093141	*HFE*	G	A	0.0615	0.0080	8.25E-14	-0.0061	0.0084	0.4723
rs2255141	10	113933886	*GPAM*	A	G	0.0299	0.0040	1.32E-13	0.0008	0.004	0.8374
rs10490626	2	118835841	*INSIG2*	G	A	0.0508	0.0069	1.70E-12	-0.0144	0.0069	0.0378
rs12748152	1	27138393	*PIGV*	T	C	0.0499	0.0066	3.21E-12	-0.0092	0.0076	0.2281
rs4530754	5	122855416	*CSNK1G3*	A	G	0.0275	0.0036	3.58E-12	-0.0047	0.0037	0.2002
rs11136341	8	145043543	*PLEC*	G	A	0.0447	0.0062	7.11E-12	-0.0081	0.0047	0.0893
rs514230	1	234858597	*IRF2BP2*	T	A	0.0364	0.0054	9.24E-12	-0.0010	0.0036	0.7856
rs1801689	17	64210580	*APOH*	C	A	0.1028	0.0139	9.81E-12	0.0023	0.0153	0.8829
rs11065987	12	112072424	*BRAP*	A	G	0.0269	0.0038	1.20E-11	0.0043	0.0039	0.2691
rs4942486	13	32953388	*BRCA2*	T	C	0.0243	0.0037	2.26E-11	0.0092	0.0037	0.0124
rs10102164	8	55421614	*SOX17*	A	G	0.0316	0.0045	3.74E-11	0.0018	0.0047	0.7096
rs2642442	1	220973563	*MOSC1*	T	C	0.0360	0.0054	5.27E-11	-0.0009	0.0040	0.8301

to be continued…

Supplementary Table 2 continued

						Results from the GWAS meta-analysis on					
						LDL-C			ApoA-IV		
**SNP**	**Chr**	**Pos**	**Nearest gene**	**A1**	**A2**	**Beta***	**SE***	**P-value**	**Beta§**	**SE§**	**P-value**
rs314253	17	7091650	*DLG4*	T	C	0.0242	0.0038	3.44E-10	-0.0029	0.0038	0.4430
rs364585	20	12962718	*SPTLC3*	G	A	0.0249	0.0038	4.28E-10	-0.0020	0.0039	0.6034
rs3780181	9	2640759	*VLDLR*	A	G	0.0445	0.0074	1.76E-09	0.0021	0.0073	0.7707
rs17404153	3	132163200	*DNAJC13*	G	T	0.0336	0.0054	1.83E-09	-0.0033	0.0055	0.5492
rs267733	1	150958836	*ANXA9*	A	G	0.0331	0.0053	5.29E-09	0.0046	0.0053	0.3867
rs2328223	20	17845921	*SNX5*	C	A	0.0299	0.0050	5.63E-09	-0.0056	0.0050	0.2627
rs2710642	2	63149557	*EHBP1*	A	G	0.0239	0.0038	6.09E-09	-0.0018	0.0038	0.6415
rs2030746	2	121309488	*GLI2*	T	C	0.0214	0.0038	8.61E-09	-0.0005	0.0040	0.9047
rs7640978	3	32533010	*CMTM6*	C	T	0.0392	0.0069	9.84E-09	-0.0023	0.0067	0.7310
rs5763662	22	30378703	*MTMR3*	T	C	0.0767	0.0121	1.19E-08	0.0067	0.0121	0.5835
rs1250229	2	216304384	*FN1*	C	T	0.0243	0.0042	3.13E-08	-0.0032	0.0042	0.4513
rs4253772	22	46627603	*PPARA*	T	C	0.0313	0.0060	4.33E-08	0.0061	0.0056	0.2760
rs11563251	2	234679384	*HEATR7B1*	T	C	0.0345	0.0062	4.50E-08	0.0058	0.0068	0.3954
rs2081687	8	59388565	*CYP7A1*	T	C	0.0311	0.0054	1.19E-07	-0.0014	0.0038	0.7159
rs9488822	6	116312893	*FRK*	A	T	0.0311	0.0054	1.69E-07	-0.0017	0.0039	0.6638
rs7206971	17	45425115	*C17orf57*	A	G	0.0292	0.0055	3.05E-07	0.0097	0.0036	0.0076
rs6831256	4	3473139	*DOK7*	G	A	0.0188	0.0038	9.07E-07	-0.0063	0.0043	0.1401
rs2277862	20	34152782	*FER1L4*	C	T	0.0271	0.0054	1.30E-06	0.0046	0.0050	0.3519

A1: effect allele; A2: other allele.

*units in SD; §units in log(apoA-IV).

Supplementary Table 3: Characteristics and results of the 40 SNPs associated with TG [1,8]. The table is sorted by the p-value for TG.

						Results from the GWAS meta-analysis on					
						TG			ApoA-IV		
SNP	Chr	Pos	Nearest gene	A1	A2	Beta*	SE*	P-value	Beta§	SE§	P-value
rs1260326	2	27730940	*GCKR*	T	C	0.1148	0.0034	2.29E-239	-0.0058	0.0036	0.1092
rs964184	11	116648917	*ZPR1*	G	C	0.2341	0.0069	6.55E-224	-0.0198	0.0052	0.0001
rs12678919	8	19844222	*LPL*	A	G	0.1702	0.0056	1.82E-199	-0.0095	0.0059	0.1053
rs2954029	8	126490972	*TRIB1*	A	T	0.0764	0.0033	1.02E-107	-0.0036	0.0036	0.3220
rs17145738	7	72982874	*TBL2*	C	T	0.1149	0.0053	9.42E-99	-0.0071	0.0057	0.2152
rs2131925	1	63025942	*DOCK7*	T	G	0.0657	0.0035	3.05E-74	-0.0026	0.0039	0.4977
rs10401969	19	19407718	*SF4*	T	C	0.1210	0.0065	9.70E-70	-0.0107	0.0071	0.1351
rs174546	11	61569830	*FADS1*	T	C	0.0447	0.0034	7.42E-38	-0.0028	0.0039	0.4712
rs6065906	20	44554015	*PCIF1*	C	T	0.0534	0.0043	2.36E-34	0.0040	0.0048	0.4024
rs4846914	1	230295691	*GALNT2*	G	A	0.0401	0.0034	7.20E-31	-0.0021	0.0037	0.5637
rs3764261	16	56993324	*CETP*	C	A	0.0395	0.0038	1.58E-25	-0.0043	0.0043	0.3175
rs442177	4	88030261	*AFF1*	T	G	0.0309	0.0033	1.32E-18	0.0057	0.0037	0.1230
rs9686661	5	55861786	*MAP3K1*	T	C	0.0379	0.0044	2.54E-16	0.0032	0.0046	0.4889
rs6882076	5	156390297	*TIMD4*	C	T	0.0286	0.0035	1.51E-15	0.0009	0.0037	0.8102
rs2972146	2	227100698	*IRS1*	T	G	0.0281	0.0034	2.97E-15	0.0027	0.0038	0.4814
rs998584	6	43757896	*VEGFA*	A	C	0.0293	0.0037	3.42E-15	-0.0022	0.0042	0.5968
rs11613352	12	57792580	*R3HDM2*	C	T	0.0280	0.0039	9.40E-14	0.0009	0.0044	0.8307
rs6831256	4	3473139	*DOK7*	G	A	0.0258	0.0035	1.60E-12	-0.0063	0.0043	0.1401
rs1832007	10	5254847	*AKR1C4*	A	G	0.0327	0.0047	1.72E-12	0.0004	0.0052	0.9346
rs645040	3	135926622	*MSL2*	T	G	0.0293	0.004	1.83E-12	-0.0045	0.0043	0.2965
rs1495741	8	18272881	*NAT2*	G	A	0.0399	0.0056	2.73E-12	0.0002	0.0043	0.9535
rs10761731	10	65027610	*JMJD1C*	A	T	0.0312	0.0047	8.40E-12	-0.0108	0.0037	0.0039
rs2412710	15	42683787	*CAPN3*	A	G	0.0988	0.0132	1.66E-11	-0.0148	0.0121	0.2219
rs2068888	10	94839642	*CYP26A1*	G	A	0.0241	0.0034	1.68E-11	-0.0077	0.0040	0.0555
rs11776767	8	10683929	*SOX7*	C	G	0.0220	0.0035	2.87E-11	0.0063	0.0038	0.0958
rs7248104	19	7224431	*INSR*	G	A	0.0222	0.0034	5.05E-10	-0.0025	0.0037	0.4870
rs12748152	1	27138393	*PIGV*	T	C	0.0372	0.0059	1.10E-09	-0.0092	0.0076	0.2281
rs4722551	7	25991826	*MIR148A*	T	C	0.0267	0.0044	1.58E-09	-0.0058	0.0051	0.2603
rs2929282	15	44245931	*FRMD5*	T	A	0.0725	0.0121	1.95E-09	-0.0035	0.0083	0.6703
rs731839	19	33899065	*PEPD*	G	A	0.0224	0.0036	2.65E-09	-0.0019	0.0039	0.6304
rs8077889	17	41878166	*MPP3*	C	A	0.0252	0.0042	9.88E-09	0.0052	0.0043	0.2333
rs38855	7	116358044	*MET*	A	G	0.0187	0.0033	2.11E-08	-0.0022	0.0036	0.5377
rs3198697	16	15129940	*PDXDC1*	C	T	0.0198	0.0034	2.21E-08	-0.0015	0.0037	0.6871
rs4765127	12	124460167	*ZNF664*	G	T	0.0286	0.0049	2.41E-08	0.0076	0.0037	0.0424
rs5756931	22	38546033	*PLA2G6*	T	C	0.0203	0.0035	2.52E-08	-0.0011	0.0038	0.7652
rs11649653	16	30918487	*MIR762*	C	G	0.0274	0.0050	1.56E-07	-0.007	0.0037	0.0609
rs1936800	6	127436064	*RSPO3*	T	C	0.0175	0.0033	3.79E-07	0.0031	0.0036	0.3956
rs1121980	16	53809247	*FTO*	A	G	0.0171	0.0034	1.52E-06	-0.0041	0.0036	0.2583
rs13238203	7	72129667	*TYW1B*	C	T	0.0592	0.0140	3.07E-06	-0.0102	0.0168	0.5455
rs838880	12	125261593	*SCARB1*	T	C	0.0059	0.0037	0.3064	-0.0073	0.0051	0.1584

A1: effect allele; A2: other allele.

*units in SD; §units in log(apoA-IV).

Supplementary Table 4: Characteristics and results of the 53 SNPs associated with eGFR [3,8]. The table is sorted by the p-value for eGFR.

						Results from the GWAS meta-analysis on					
							eGFR			ApoA-IV	
**SNP**	**Chr**	**Pos**	**Nearest gene**	**A1**	**A2**	**Beta***	**SE***	**P-value**	**Beta§**	**SE§**	**P-value**
rs13329952	16	20366507	*UMOD*	T	C	-0.0160	0.0011	9.50E-43	-0.0129	0.0048	0.0069
rs2467853	15	45698793	*SPATA5L1*	T	G	0.0130	0.0009	1.00E-42	-0.0069	0.0037	0.0636
rs17319721	4	77368847	*SHROOM3*	A	G	-0.0110	0.0009	1.30E-37	-0.0056	0.0037	0.1266
rs7805747	7	151407801	*PRKAG2*	A	G	-0.0130	0.0011	8.00E-29	-0.0027	0.0040	0.4942
rs7422339	2	211540507	*CPS1*	A	C	-0.0110	0.0010	2.20E-23	-0.0010	0.0041	0.7985
rs6420094	5	176817636	*SLC34A1*	A	G	0.0096	0.0010	4.90E-22	-0.0068	0.0050	0.1754
rs11657044	17	59450105	*BCAS3*	T	C	-0.0110	0.0012	7.90E-22	-0.0098	0.0050	0.0486
rs11959928	5	39397132	*FYB*	A	T	-0.0083	0.0009	1.70E-20	0.0008	0.0037	0.8314
rs6546838	2	73679280	*ALMS1*	A	G	-0.0093	0.0010	7.70E-20	-0.0004	0.0043	0.9222
rs316009	6	160675764	*SLC22A2*	T	C	0.0130	0.0014	4.40E-19	0.0015	0.0061	0.8113
rs963837	11	30749090	*DCDC5*	T	C	-0.0078	0.0009	5.70E-18	-0.0033	0.0037	0.3795
rs3758086	8	23714992	*STC1*	A	G	-0.0071	0.0009	1.70E-15	-0.0058	0.0037	0.1161
rs491567	15	53946593	*WDR72*	A	C	-0.0084	0.0010	2.90E-15	0.0007	0.0045	0.8697
rs9472135	6	43809802	*VEGFA*	T	C	-0.0080	0.0010	3.30E-15	-0.0023	0.0042	0.5823
rs4744712	9	71434707	*PIP5K1B*	A	C	-0.0071	0.0009	4.30E-15	0.0048	0.0037	0.1918
rs9916302	17	37499949	*FBXL20*	T	C	-0.0080	0.0010	4.80E-15	-0.0078	0.0042	0.0631
rs1260326	2	27730940	*GCKR*	T	C	0.0068	0.0009	3.40E-14	-0.0058	0.0036	0.1092
rs1394125	15	76158983	*UBE2Q2*	A	G	-0.0073	0.0010	5.50E-14	-0.0005	0.0042	0.9061
rs2861422	3	141724644	*TFDP2*	T	C	0.0074	0.0010	9.10E-14	-0.0063	0.0041	0.1226
rs12460876	19	33356891	*SLC7A9*	T	C	-0.0066	0.0009	1.90E-13	-0.0077	0.0038	0.0414
rs267734	1	150951477	*ANXA9*	T	C	-0.0079	0.0011	4.00E-13	-0.0062	0.0045	0.1740
rs17216707	20	52732362	*CYP24A1*	T	C	-0.0084	0.0011	6.00E-13	-0.0090	0.0047	0.0533
rs848490	7	77555005	*PHTF2*	C	G	0.0073	0.0010	7.80E-13	-0.0041	0.0041	0.3122
rs10774021	12	349298	*SLC6A13*	T	C	-0.0063	0.0009	4.80E-12	-0.0003	0.0039	0.9478
rs807601	2	15793014	*DDX1*	T	G	0.0064	0.0009	6.60E-12	-0.0095	0.0038	0.0129
rs1044261	10	1065710	*C10orf110*	T	C	-0.011	0.0016	1.20E-11	0.0010	0.0075	0.8948
rs4014195	11	65506822	*KRT8P26*	C	G	0.0061	0.0009	2.20E-11	-0.0063	0.0038	0.0970
rs2453580	17	19438321	*SLC47A1*	T	C	0.0064	0.0009	2.90E-11	0.0003	0.0046	0.9433
rs10277115	7	1285195	*UNCX*	A	T	0.0095	0.0014	1.10E-10	-0.0098	0.0065	0.1315
rs10994860	10	52645424	*A1CF*	T	C	0.0075	0.0011	1.20E-10	-0.0002	0.0048	0.9694
rs6459680	7	156258568	*C7orf13*	T	G	-0.0065	0.0010	2.00E-10	-0.0025	0.0042	0.5552
rs10491967	12	3368093	*TSPAN9*	A	G	-0.0092	0.0014	3.00E-10	0.0074	0.006	0.2179
rs228611	4	103561709	*NFKB1*	A	G	-0.0055	0.0009	4.70E-10	-0.0045	0.0037	0.2149
rs6088580	20	33285053	*TP53INP2*	C	G	-0.0055	0.0009	7.20E-10	-0.0026	0.0037	0.4764
rs1800615	1	15832281	*CASP9*	T	C	-0.0058	0.0009	1.90E-09	-0.0033	0.0039	0.4011
rs7956634	12	15321194	*RERG*	T	C	-0.0068	0.0011	2.50E-09	-0.0032	0.0051	0.5277
rs3850625	1	201016296	*CACNA1S*	A	G	0.008	0.0014	6.40E-09	-0.0055	0.0056	0.3264
rs476633	15	41392134	*INO80*	C	G	0.0051	0.0009	8.90E-09	-0.0004	0.0037	0.9220
rs6795744	3	13906850	*WNT7A*	A	G	0.0071	0.0012	9.60E-09	-0.0031	0.0052	0.5444
rs163160	11	2789955	*KCNQ1*	A	G	0.0067	0.0011	9.70E-09	-0.0053	0.0048	0.2710

to be continued…

Supplementary Table 4 continued

						Results from the GWAS meta-analysis on					
						eGFR			ApoA-IV		
**SNP**	**Chr**	**Pos**	**Nearest gene**	**A1**	**A2**	**Beta***	**SE***	**P-value**	**Beta§**	**SE§**	**P-value**
rs2712184	2	217682779	*TNP1*	A	C	-0.0049	0.0009	2.70E-08	-0.0002	0.0037	0.9498
rs1106766	12	57809456	*R3HDM2*	T	C	0.0062	0.0011	4.70E-08	-0.0009	0.0044	0.8303
rs716877	13	72347448	*DACH1*	C	G	0.0049	0.0009	6.20E-08	0.0082	0.0038	0.0317
rs2802729	1	243501763	*SDCCAG8*	A	C	-0.005	0.0009	7.40E-08	-0.0051	0.0038	0.1819
rs11666497	19	38464262	*SIPA1L3*	T	C	-0.0064	0.0012	8.60E-08	-0.0009	0.0047	0.8406
rs164748	16	89708292	*CHMP1A*	C	G	0.0047	0.0009	9.30E-08	-0.0039	0.0037	0.2925
rs10513801	3	185822353	*ETV5*	T	G	0.007	0.0013	9.30E-08	0.0001	0.0056	0.9805
rs12136063	1	110014170	*SYPL2*	A	G	0.0049	0.0009	2.30E-07	0.0035	0.0039	0.3633
rs4667594	2	170008506	*LRP2*	A	T	-0.0045	0.0009	2.40E-07	0.0045	0.0036	0.2112
rs3750082	7	32919927	*RP9P*	A	T	0.0049	0.0009	2.50E-07	0.0045	0.0039	0.2509
rs7759001	6	27341409	*ZNF391*	A	G	-0.0053	0.0010	2.60E-07	0.0001	0.0043	0.9842
rs8091180	18	77164243	*NFATC1*	A	G	-0.0054	0.0010	3.50E-07	-0.0058	0.0056	0.2970
rs9682041	3	170091902	*SKIL*	T	C	-0.0067	0.0013	3.80E-07	-0.0037	0.0054	0.4933

A1: effect allele; A2: other allele.

*units in log(eGFR); §units in log(apoA-IV).

Supplementary Table 5: Characteristics and results of the 77 SNPs associated with BMI [4,8]. The table is sorted by the p-value for BMI.

						Results from the GWAS meta-analysis on					
						BMI			ApoA-IV		
SNP	Chr	Pos	Nearest gene	A1	A2	Beta*	SE*	P-value	Beta§	SE§	P-value
rs1558902	16	53803574	*FTO*	A	T	0.0818	0.0031	7.51E-153	-0.0047	0.0037	0.2020
rs6567160	18	57829135	*MC4R*	C	T	0.0556	0.0036	3.93E-53	0.0053	0.0042	0.2124
rs13021737	2	632348	*TMEM18*	A	G	-0.0601	0.0040	1.11E-50	-0.0023	0.0047	0.6214
rs10938397	4	45182527	*GNPDA2*	A	G	-0.0402	0.0031	3.21E-38	-0.0032	0.0038	0.3917
rs543874	1	177889480	*SEC16B*	G	A	0.0482	0.0039	2.62E-35	0.0066	0.0047	0.1650
rs2207139	6	50845490	*TFAP2B*	G	A	0.0447	0.0040	4.13E-29	0.0011	0.0049	0.8146
rs11030104	11	27684517	*LIN7C*	A	G	0.0414	0.0038	5.56E-28	0.0014	0.0043	0.7515
rs3101336	1	72751185	*NEGR1*	T	C	-0.0334	0.0031	2.66E-26	0.0059	0.0038	0.1250
rs7138803	12	50247468	*BCDIN3D*	G	A	-0.0315	0.0031	8.15E-24	0.0032	0.0038	0.4074
rs10182181	2	25150296	*ADCY3*	A	G	-0.0307	0.0031	8.78E-24	0.0022	0.0037	0.5530
rs3888190	16	28889486	*ATP2A1*	A	C	0.0309	0.0031	3.14E-23	0.0046	0.0046	0.3185
rs1516725	3	185824004	*TRA2B*	T	C	-0.0451	0.0046	1.89E-22	0.0000	0.0055	0.9956
rs12446632	16	19935389	*GPRC5B*	A	G	-0.0403	0.0046	1.48E-18	-0.0043	0.0052	0.4029
rs2287019	19	46202172	*QPCTL*	C	T	0.0360	0.0042	4.59E-18	0.0089	0.0057	0.1169
rs16951275	15	68077168	*MAP2K5*	C	T	-0.0311	0.0037	1.91E-17	0.0034	0.0043	0.4360
rs3817334	11	47650993	*MTCH2*	C	T	-0.0262	0.0031	5.15E-17	-0.0003	0.0037	0.9354
rs2112347	5	75015242	*C5orf37*	G	T	-0.0261	0.0031	6.19E-17	-0.0001	0.0038	0.9795
rs12566985	1	75002193	*LRRC53*	G	A	0.0242	0.0031	3.28E-15	-0.0003	0.0036	0.9285
rs3810291	19	47569003	*ZC3H4*	A	G	0.0283	0.0036	4.81E-15	-0.0035	0.0043	0.4228
rs7141420	14	79899454	*NRXN3*	T	C	0.0235	0.0031	1.23E-14	-0.0006	0.0036	0.8679
rs13078960	3	85807590	*CADM2*	T	G	-0.0297	0.0039	1.74E-14	-0.0069	0.0046	0.1286
rs10968576	9	28414339	*LINGO2*	G	A	0.0249	0.0033	6.61E-14	-0.0013	0.0040	0.7444
rs17024393	1	110154688	*GNAT2*	C	T	0.0658	0.0088	7.03E-14	0.0005	0.0108	0.9598
rs657452	1	49589847	*AGBL4*	A	G	0.0227	0.0031	5.48E-13	0.0026	0.0037	0.4835
rs12429545	13	54102206	*OLFM4*	G	A	-0.0334	0.0047	1.09E-12	-0.0085	0.0060	0.1528
rs12286929	11	115022404	*CADM1*	G	A	0.0217	0.0031	1.31E-12	-0.0040	0.0036	0.2720
rs13107325	4	103188709	*SLC39A8*	C	T	-0.0477	0.0068	1.83E-12	-0.0014	0.0072	0.8452
rs11165643	1	96924097	*PTBP2*	C	T	-0.0218	0.0031	2.07E-12	-0.0013	0.0036	0.7126
rs7903146	10	114758349	*TCF7L2*	T	C	-0.0234	0.0034	1.11E-11	-0.0074	0.0040	0.0626
rs10132280	14	25928179	*STXBP6*	A	C	-0.0230	0.0034	1.14E-11	0.0092	0.0040	0.0209
rs17405819	8	76806584	*HNF4G*	C	T	-0.0224	0.0033	2.07E-11	-0.0044	0.0039	0.2583
rs1016287	2	59305625	*EIF3FP3*	T	C	0.0229	0.0034	2.25E-11	-0.0041	0.0040	0.3092
rs4256980	11	8673939	*TRIM66*	G	C	0.0209	0.0031	2.90E-11	-0.0047	0.0037	0.2026
rs17094222	10	102395440	*HIF1AN*	C	T	0.0249	0.0038	5.94E-11	0.0032	0.0047	0.4966
rs12401738	1	78446761	*FUBP1*	A	G	0.0211	0.0033	1.15E-10	-0.0009	0.0040	0.8132
rs7599312	2	213413231	*MIR548F2*	G	A	0.0220	0.0034	1.17E-10	0.0030	0.0041	0.4586
rs2365389	3	61236462	*FHIT*	C	T	0.0200	0.0031	1.63E-10	-0.0022	0.0037	0.5494
rs205262	6	34563164	*C6orf106*	A	G	-0.0221	0.0035	1.75E-10	0.0031	0.0041	0.4481
rs2820292	1	201784287	*NAV1*	A	C	-0.0195	0.0031	1.83E-10	0.0030	0.0037	0.4210
rs12885454	14	29736838	*PRKD1*	C	A	0.0207	0.0033	1.94E-10	0.0004	0.0038	0.9144

to be continued…

Supplementary Table 5 continued

						Results from the GWAS meta-analysis on					
							BMI			ApoA-IV	
SNP	Chr	Pos	Nearest gene	A1	A2	Beta*	SE*	P-value	Beta§	SE§	P-value
rs12016871	13	28017782	*MTIF3*	C	T	-0.0298	0.0047	2.29E-10	-0.0091	0.0046	0.0480
rs16851483	3	141275436	*RASA2*	G	T	-0.0483	0.0077	3.55E-10	0.0060	0.0077	0.4360
rs1167827	7	75163169	*HIP1*	A	G	-0.0202	0.0033	6.33E-10	0.0033	0.0045	0.4617
rs758747	16	3627358	*NLRC3*	C	T	-0.0225	0.0037	7.47E-10	-0.0011	0.0051	0.8256
rs1928295	9	120378483	*TLR4*	C	T	-0.0188	0.0031	7.91E-10	0.0051	0.0036	0.1570
rs9925964	16	31129895	*MYST1*	G	A	-0.0192	0.0031	8.11E-10	-0.0067	0.0038	0.0726
rs11126666	2	26928811	*KCNK3*	G	A	-0.0207	0.0034	1.33E-09	-0.0009	0.0042	0.8208
rs2650492	16	28333411	*SBK1*	A	G	0.0207	0.0035	1.92E-09	0.0050	0.0052	0.3342
rs6804842	3	25106437	*RARB*	A	G	-0.0185	0.0031	2.48E-09	-0.0013	0.0037	0.7170
rs12940622	17	78615571	*RPTOR*	A	G	-0.0182	0.0031	2.49E-09	-0.0006	0.0036	0.8686
rs11847697	14	30515112	*PRKD1*	T	C	0.0492	0.0084	3.99E-09	0.0013	0.0094	0.8854
rs4740619	9	15634326	*C9orf93*	T	C	0.0179	0.0031	4.56E-09	0.0058	0.0036	0.1126
rs13191362	6	163033350	*PARK2*	A	G	0.0277	0.0048	7.34E-09	-0.0029	0.0058	0.6145
rs3736485	15	51748610	*DMXL2*	A	G	0.0176	0.0031	7.41E-09	0.0077	0.0037	0.0371
rs17001654	4	77129568	*SCARB2*	C	G	-0.0306	0.0053	7.76E-09	0.0041	0.0049	0.4050
rs11191560	10	104869038	*NT5C2*	T	C	-0.0308	0.0053	8.45E-09	-0.0006	0.0060	0.9178
rs1528435	2	181550962	*UBE2E3*	T	C	0.0178	0.0031	1.20E-08	0.0043	0.0038	0.2632
rs2075650	19	45395619	*TOMM40*	A	G	0.0258	0.0045	1.25E-08	0.0061	0.0062	0.3222
rs1000940	17	5283252	*RABEP1*	G	A	0.0192	0.0034	1.28E-08	-0.0007	0.0040	0.8663
rs2033529	6	40348653	*LRFN2*	G	A	0.0190	0.0033	1.39E-08	0.0026	0.0040	0.5126
rs11583200	1	50559820	*ELAVL4*	C	T	0.0177	0.0031	1.48E-08	0.0075	0.0038	0.0455
rs9400239	6	108977663	*FOXO3*	C	T	0.0188	0.0033	1.61E-08	0.0007	0.0039	0.8503
rs10733682	9	129460914	*LMX1B*	A	G	0.0174	0.0031	1.83E-08	-0.0011	0.0040	0.7850
rs11688816	2	63053048	*EHBP1*	A	G	-0.0172	0.0031	1.89E-08	-0.0015	0.0036	0.6785
rs11057405	12	122781897	*CLIP1*	A	G	-0.0307	0.0055	2.02E-08	0.0041	0.0076	0.5888
rs2121279	2	143043285	*LRP1B*	T	C	0.0245	0.0044	2.31E-08	0.0058	0.0055	0.2909
rs29941	19	34309532	*KCTD15*	A	G	-0.0182	0.0033	2.41E-08	0.0019	0.0039	0.6265
rs11727676	4	145659064	*HHIP*	C	T	-0.0358	0.0064	2.55E-08	0.0149	0.0082	0.0680
rs3849570	3	81792112	*GBE1*	A	C	0.0188	0.0034	2.60E-08	-0.0102	0.0039	0.0090
rs6477694	9	111932342	*EPB41L4B*	C	T	0.0174	0.0031	2.67E-08	0.0021	0.0037	0.5772
rs7899106	10	87410904	*GRID1*	A	G	-0.0395	0.0071	2.96E-08	0.0071	0.0086	0.4130
rs2176598	11	43864278	*HSD17B12*	T	C	0.0198	0.0036	2.97E-08	-0.0039	0.0043	0.3622
rs2245368	7	76608143	*PMS2L11*	T	C	-0.0317	0.0057	3.19E-08	0.0016	0.0075	0.8352
rs17724992	19	18454825	*PGPEP1*	A	G	0.0194	0.0035	3.42E-08	-0.0011	0.0042	0.7885
rs7243357	18	56883319	*GRP*	G	T	-0.0217	0.004	3.86E-08	-0.0014	0.0048	0.7725
rs1808579	18	21104888	*C18orf8*	T	C	-0.0167	0.0031	4.17E-08	-0.0018	0.0036	0.6117
rs2033732	8	85079709	*RALYL*	C	T	0.0192	0.0035	4.89E-08	-0.0058	0.0042	0.1696

A1: effect allele; A2: other allele.

*units in SD; §units in log(apoA-IV).

Supplementary Table 6: Characteristics and results of the 40 SNPs associated with WHR [5,8]. The table is sorted by the p-value for WHR.

						Results from the GWAS meta-analysis on					
						WHR			ApoA-IV		
SNP	Chr	Pos	Nearest gene	A1	A2	Beta	SE	P-value	Beta	SE	P-value
rs2645294	1	119574587	*WARS2*	C	T	-0.032	0.0034	1.30E-20	-0.0049	0.0036	0.1733
rs979012	20	6623374	*BMP2*	T	C	0.033	0.0036	5.40E-20	-0.0057	0.0039	0.1381
rs1936805	6	127452116	*RSPO3*	T	C	0.029	0.0034	8.40E-18	0.0022	0.0036	0.5481
rs10245353	7	25858614	*MIR148A*	A	C	0.033	0.0043	5.80E-15	0.0070	0.0048	0.1440
rs1358980	6	43764551	*VEGFA*	T	C	0.027	0.0036	3.20E-14	-0.0031	0.0042	0.4577
rs17451107	3	156797609	*LEKR1*	T	C	0.026	0.0036	1.30E-13	-0.0013	0.0037	0.7138
rs6556301	5	176527577	*FGFR4*	T	G	0.028	0.0039	1.80E-12	0.0050	0.0047	0.2934
rs12679556	8	72514228	*MSC*	G	T	0.026	0.0039	1.30E-11	-0.0015	0.0042	0.7189
rs7801581	7	27223771	*HOXA11*	T	C	0.027	0.0042	8.00E-11	0.0008	0.0049	0.8637
rs7705502	5	173320815	*CPEB4*	A	G	0.023	0.0036	9.10E-11	0.0072	0.0040	0.0715
rs2371767	3	64718258	*ADAMTS9*	G	C	0.022	0.0039	6.40E-09	-0.0016	0.0039	0.6857
rs4765219	12	124440110	*CCDC92*	C	A	0.020	0.0035	9.40E-09	-0.0071	0.0038	0.0574
rs12608504	19	18389135	*JUND*	A	G	0.020	0.0036	1.50E-08	0.0013	0.0040	0.7379
rs2276824	3	52637486	*PBRM1*	C	G	0.020	0.0035	1.50E-08	-0.0007	0.0038	0.8511
rs2294239	22	29449477	*ZNRF3*	A	G	0.019	0.0035	3.50E-08	0.0045	0.0037	0.2189
rs1385167	2	66200648	*MEIS1*	G	A	0.024	0.0049	5.80E-07	-0.0039	0.0055	0.4706
rs4081724	19	33824946	*CEBPG*	G	A	0.025	0.0050	8.70E-07	0.0021	0.0051	0.6760
rs8030605	15	56504598	*RFX7*	A	G	0.024	0.0052	2.70E-06	0.0094	0.0053	0.0791
rs10842707	12	26471364	*ITPR2*	T	C	0.018	0.0040	5.80E-06	-0.0033	0.0044	0.4530
rs1443512	12	54342684	*HOXC13*	A	C	0.017	0.0039	9.20E-06	-0.0075	0.0043	0.0786
rs7759742	6	32381736	*HLA-DRB5*	A	T	0.014	0.0034	3.40E-05	0.0037	0.0049	0.4530
rs10991437	9	107735920	*ABCA1*	A	C	0.021	0.0053	5.80E-05	-0.0121	0.0058	0.0374
rs714515	1	172352990	*C1orf105*	G	A	0.012	0.0033	3.00E-04	0.0074	0.0037	0.0420
rs10919388	1	170372503	*GORAB*	A	C	-0.014	0.0040	0.00034	-0.0008	0.0040	0.8457
rs8042543	15	31708263	*KLF13*	C	T	0.015	0.0042	0.00035	-0.0043	0.0054	0.4221
rs10195252	2	165513091	*COBLL1*	T	C	0.0110	0.0034	0.00094	-0.0001	0.0038	0.9822
rs1294410	6	6738752	*LY86*	C	T	0.0110	0.0035	0.00094	-0.0024	0.0038	0.5321
rs303084	4	124066948	*SPATA5*	A	G	0.0140	0.0042	0.0012	-0.0020	0.0043	0.6436
rs4646404	17	17420199	*PEMT*	G	A	0.0120	0.0039	0.0015	-0.0008	0.0041	0.8485
rs905938	1	154991389	*DCST2*	C	T	-0.0120	0.0040	0.0021	0.0068	0.0046	0.1386
rs6090583	20	45558831	*EYA2*	A	G	0.0100	0.0034	0.0022	0.0001	0.0037	0.9851
rs224333	20	34023962	*GDF5*	G	A	-0.0100	0.0036	0.0053	0.0005	0.0038	0.8961
rs1569135	2	188115398	*CALCRL*	A	G	0.0094	0.0034	0.0056	0.0005	0.0036	0.8909
rs11231693	11	63862612	*MACROD1*	A	G	0.0200	0.0075	0.0085	0.0200	0.0084	0.0177
rs1440372	15	67033151	*SMAD6*	C	T	0.0094	0.0037	0.0120	0.0008	0.0043	0.8515
rs1045241	5	118729286	*TNFAIP8*	C	T	0.0065	0.0037	0.0830	-0.0058	0.0040	0.1520
rs9991328	4	89713121	*FAM13A*	T	C	0.0058	0.0034	0.0890	0.0035	0.0037	0.3434
rs17819328	3	12489342	*PPARG*	G	T	0.0054	0.0035	0.1200	-0.0016	0.0037	0.6706
rs10804591	3	129334233	*PLXND1*	A	C	-0.0026	0.0042	0.5400	0.0013	0.0045	0.7707
rs2820443	1	219753509	*ZC3H11B*	C	T	-0.0001	0.0037	0.9800	0.0005	0.0041	0.8998

A1: effect allele; A2: other allele.

*units in SD; [§]units in log(apoA-IV).

Supplementary Table 7: Characteristics and results of the 36 SNPs associated with fasting glucose [6,8]. The table is sorted by the p-value for fasting glucose.

						Results from the GWAS meta-analysis on					
						Fasting Glucose			ApoA-IV		
SNP	Chr	Pos	Nearest gene	A1	A2	Beta*	SE*	P-value	Beta[§]	SE[§]	P-value
rs10830963	11	92708710	*MTNR1B*	C	G	-0.078	0.0025	1.07E-215	-0.0043	0.0043	0.3218
rs560887	2	169763148	*ABCB11*	C	T	0.071	0.0025	1.40E-178	0.0014	0.0040	0.7196
rs2908289	7	44223942	*GCK*	G	A	-0.057	0.0029	3.32E-88	-0.0013	0.0048	0.7923
rs2191349	7	15064309	*DGKB*	G	T	-0.029	0.0021	1.28E-42	0.0001	0.0036	0.9722
rs780094	2	27741237	*GCKR*	C	T	0.027	0.0021	2.58E-37	0.0074	0.0036	0.0411
rs11558471	8	118185733	*SLC30A8*	A	G	0.029	0.0023	7.80E-37	-0.0019	0.0041	0.6405
rs4502156	15	62383155	*C2CD4A*	T	C	0.022	0.0021	1.38E-25	-0.0016	0.0037	0.6682
rs11607883	11	45839709	*SLC35C1*	G	A	0.021	0.0021	6.32E-24	-0.0024	0.0036	0.5115
rs11039182	11	47346723	*MADD*	T	C	0.023	0.0024	4.82E-22	-0.0013	0.0040	0.7400
rs7903146	10	114758349	*TCF7L2*	C	T	-0.022	0.0024	2.71E-20	0.0074	0.0040	0.0626
rs174576	11	61603510	*FADS2*	C	A	0.020	0.0022	1.18E-18	0.0020	0.0040	0.6230
rs11708067	3	123065778	*ADCY5*	A	G	0.023	0.0026	1.30E-18	0.0146	0.0045	0.0011
rs11195502	10	113039667	*ADRA2A*	C	T	0.032	0.0037	1.97E-18	-0.0062	0.0065	0.3403
rs10811661	9	22134094	*CDKN2B*	T	C	0.024	0.0028	5.65E-18	-0.0025	0.0048	0.6096
rs1280	3	170713290	*SLC2A2*	T	C	0.026	0.0031	8.56E-18	-0.0058	0.0052	0.2686
rs4869272	5	95539448	*MIR583*	C	T	-0.018	0.0022	1.02E-15	-0.0011	0.0039	0.7699
rs11619319	13	28487599	*PDX1*	A	G	-0.020	0.0024	1.33E-15	0.0017	0.0045	0.6953
rs983309	8	9177732	*PPP1R3B*	G	T	-0.026	0.0033	6.29E-15	0.0007	0.0059	0.9056
rs10814916	9	4293150	*GLIS3*	C	A	0.016	0.0022	2.26E-13	0.0088	0.0036	0.0149
rs6943153	7	50791579	*GRB10*	C	T	-0.015	0.0022	1.63E-12	-0.0003	0.0040	0.9421
rs11603334	11	72432985	*ARAP1*	G	A	0.019	0.0028	1.12E-11	-0.0108	0.0052	0.0363
rs6113722	20	22557099	*FOXA2*	G	A	0.035	0.0053	2.49E-11	0.0092	0.0085	0.2802
rs16913693	9	111680359	*IKBKAP*	T	G	0.043	0.0066	3.51E-11	0.0039	0.0107	0.7181
rs3829109	9	139256766	*GPSM1*	G	A	0.017	0.0027	1.13E-10	-0.0042	0.0055	0.4413
rs3783347	14	100839261	*WARS*	G	T	0.017	0.0026	1.32E-10	-0.0055	0.0045	0.2199
rs340874	1	214159256	*PROX1*	C	T	0.013	0.0022	4.08E-10	0.002	0.0039	0.6108
rs2302593	19	46196634	*QPCTL*	C	G	0.014	0.0023	9.26E-10	0.0052	0.0045	0.2390
rs9368222	6	20686996	*CDKAL1*	C	A	-0.014	0.0023	1.00E-09	0.0074	0.0041	0.0673
rs10747083	12	133041618	*FBRSL1*	A	G	0.013	0.0023	7.57E-09	0.0072	0.0054	0.1833
rs6072275	20	39743905	*TOP1*	G	A	-0.016	0.0028	1.66E-08	0.0015	0.0051	0.7704
rs7651090	3	185513392	*IGF2BP2*	A	G	-0.013	0.0023	1.75E-08	0.0019	0.0039	0.6353
rs576674	13	33554302	*KL*	G	A	0.017	0.0030	2.26E-08	-0.0034	0.0051	0.5075
rs11715915	3	49455330	*AMT*	C	T	0.012	0.0022	4.90E-08	-0.0051	0.0039	0.1901
rs17762454	6	7213200	*RREB1*	C	T	-0.012	0.0023	1.88E-07	-0.0010	0.0042	0.8072
rs7708285	5	76425867	*SNORA47*	G	A	0.011	0.0025	4.89E-06	0.0053	0.0043	0.2217
rs2657879	12	56865338	*GLS2*	A	G	-0.012	0.0027	5.69E-06	-0.0012	0.0045	0.7896

A1: effect allele; A2: other allele.

*units in mmol/L; [§]units in log(apoA-IV).

Supplementary Table 8: Location and association p-values for the SNPs associated with HDL-C (Trait 1) that are in LD (r^2>0.8) with SNPs associated with the other traits (denoted as Trait 2). The table is sorted by Trait 2 and within Trait 2 by the SNP name of Trait 1.

Trait 1	SNP	Chr	Pos	Nearest gene	P-value	Trait 2	SNP	Chr	Pos	Nearest gene	P-value	R^2
**HDL-C**	rs1121980	16	53809247	*FTO*	6.79E-09	**BMI**	rs1558902	16	53803574	*FTO*	7.51E-153	0.956547
**HDL-C**	rs12967135	18	57849023	*MC4R*	3.57E-08	**BMI**	rs6567160	18	57829135	*MC4R*	3.93E-53	1
**HDL-C**	rs13107325	4	103188709	*SLC39A8*	1.07E-15	**BMI**	rs13107325	4	103188709	*SLC39A8*	1.83E-12	1
**HDL-C**	rs1047891	2	211540507	*CPS1*	8.73E-10	**eGFR**	rs7422339	2	211540507	*CPS1*	2.20E-23	1
**HDL-C**	rs11613352	12	57792580	*R3HDM2*	2.39E-13	**eGFR**	rs1106766	12	57809456	*R3HDM2*	4.70E-08	1
**HDL-C**	rs174546	11	61569830	*FADS1*	8.30E-28	**Fasting Glucose**	rs174576	11	61603510	*FADS2*	1.18E-18	0.936276
**HDL-C**	rs12748152	1	27138393	*PIGV*	9.74E-16	**LDL-C**	rs12748152	1	27138393	*PIGV*	3.21E-12	1
**HDL-C**	rs174546	11	61569830	*FADS1*	8.30E-28	**LDL-C**	rs174546	11	61569830	*FADS1*	1.63E-39	1
**HDL-C**	rs2954029	8	126490972	*TRIB1*	2.67E-29	**LDL-C**	rs2954029	8	126490972	*TRIB1*	2.10E-50	1
**HDL-C**	rs3764261	16	56993324	*CETP*	1.00E-769	**LDL-C**	rs3764261	16	56993324	*CETP*	2.22E-34	1
**HDL-C**	rs4420638	19	45422946	*APOC1*	1.72E-21	**LDL-C**	rs4420638	19	45422946	*APOC1*	1.51E-178	1
**HDL-C**	rs964184	11	116648917	*ZNF259*	6.09E-48	**LDL-C**	rs964184	11	116648917	*ZNF259*	2.01E-26	1
**HDL-C**	rs9987289	8	9183358	*PPP1R3B*	1.95E-41	**LDL-C**	rs9987289	8	9183358	*PPP1R3B*	8.53E-24	1
**HDL-C**	rs1121980	16	53809247	*FTO*	6.79E-09	**TG**	rs1121980	16	53809247	*FTO*	1.52E-06	1
**HDL-C**	rs11613352	12	57792580	*R3HDM2*	2.39E-13	**TG**	rs11613352	12	57792580	*R3HDM2*	9.40E-14	1
**HDL-C**	rs12678919	8	19844222	*LPL*	1.38E-149	**TG**	rs12678919	8	19844222	*LPL*	1.82E-199	1
**HDL-C**	rs12748152	1	27138393	*PIGV*	9.74E-16	**TG**	rs12748152	1	27138393	*PIGV*	1.10E-09	1
**HDL-C**	rs17145738	7	72982874	*TBL2*	4.95E-13	**TG**	rs17145738	7	72982874	*TBL2*	9.42E-99	1
**HDL-C**	rs174546	11	61569830	*FADS1*	8.30E-28	**TG**	rs174546	11	61569830	*FADS1*	7.42E-38	1
**HDL-C**	rs1936800	6	127436064	*RSPO3*	3.06E-10	**TG**	rs1936800	6	127436064	*RSPO3*	3.79E-07	1
**HDL-C**	rs2954029	8	126490972	*TRIB1*	2.67E-29	**TG**	rs2954029	8	126490972	*TRIB1*	1.02E-107	1
**HDL-C**	rs2972146	2	227100698	*IRS1*	1.85E-17	**TG**	rs2972146	2	227100698	*IRS1*	2.97E-15	1
**HDL-C**	rs3764261	16	56993324	*CETP*	1.00E-769	**TG**	rs3764261	16	56993324	*CETP*	1.58E-25	1
**HDL-C**	rs4765127	12	124460167	*ZNF664*	7.79E-10	**TG**	rs4765127	12	124460167	*ZNF664*	2.41E-08	1
**HDL-C**	rs4846914	1	230295691	*GALNT2*	3.51E-41	**TG**	rs4846914	1	230295691	*GALNT2*	7.20E-31	1
**HDL-C**	rs6065906	20	44554015	*PCIF1*	5.34E-40	**TG**	rs6065906	20	44554015	*PCIF1*	2.36E-34	1
**HDL-C**	rs731839	19	33899065	*PEPD*	3.44E-09	**TG**	rs731839	19	33899065	*PEPD*	2.65E-09	1
**HDL-C**	rs838880	12	125261593	*SCARB1*	6.38E-32	**TG**	rs838880	12	125261593	*SCARB1*	0.3064	1
**HDL-C**	rs964184	11	116648917	*ZNF259*	6.09E-48	**TG**	rs964184	11	116648917	*ZNF259*	6.55E-224	1
**HDL-C**	rs998584	6	43757896	*VEGFA*	2.27E-11	**TG**	rs998584	6	43757896	*VEGFA*	3.42E-15	1

to be continued…

Supplementary Table 8 continued

Trait 1	SNP	Chr	Pos	Nearest gene	P-value	Trait 2	SNP	Chr	Pos	Nearest gene	P-value	R²
**HDL-C**	rs1936800	6	127436064	*RSPO3*	3.06E-10	**WHR**	rs1936805	6	127452116	*RSPO3*	8.40E-18	0.876715
**HDL-C**	rs3822072	4	89741269	*FAM13A*	4.06E-12	**WHR**	rs9991328	4	89713121	*FAM13A*	0.089	0.960722
**HDL-C**	rs4765127	12	124460167	*ZNF664*	7.79E-10	**WHR**	rs4765219	12	124440110	*CCDC92*	9.40E-09	0.987213
**HDL-C**	rs998584	6	43757896	*VEGFA*	2.27E-11	**WHR**	rs1358980	6	43764551	*VEGFA*	3.20E-14	0.829246

Supplementary Table 9: Location and association p-values for the SNPs associated with LDL-C (Trait 1) that are in LD (r^2>0.8) with SNPs associated with the other traits (denoted as Trait 2). The table is sorted by Trait 2 and within Trait 2 by the SNP name of Trait 1.

Trait 1	SNP	Chr	Pos	Nearest gene	P-value	Trait 2	SNP	Chr	Pos	Nearest gene	P-value	R²
**LDL-C**	rs174546	11	61569830	*FADS1*	1.63E-39	**Fasting Glucose**	rs174576	11	61603510	*FADS2*	1.18E-18	0.936276
**LDL-C**	rs12748152	1	27138393	*PIGV*	3.21E-12	**HDL-C**	rs12748152	1	27138393	*PIGV*	9.74E-16	1
**LDL-C**	rs174546	11	61569830	*FADS1*	1.63E-39	**HDL-C**	rs174546	11	61569830	*FADS1*	8.30E-28	1
**LDL-C**	rs2954029	8	126490972	*TRIB1*	2.10E-50	**HDL-C**	rs2954029	8	126490972	*TRIB1*	2.67E-29	1
**LDL-C**	rs3764261	16	56993324	*CETP*	2.22E-34	**HDL-C**	rs3764261	16	56993324	*CETP*	0	1
**LDL-C**	rs4420638	19	45422946	*APOC1*	1.51E-178	**HDL-C**	rs4420638	19	45422946	*APOC1*	1.72E-21	1
**LDL-C**	rs964184	11	116648917	*ZNF259*	2.01E-26	**HDL-C**	rs964184	11	116648917	*ZNF259*	6.09E-48	1
**LDL-C**	rs9987289	8	9183358	*PPP1R3B*	8.53E-24	**HDL-C**	rs9987289	8	9183358	*PPP1R3B*	1.95E-41	1
**LDL-C**	rs10401969	19	19407718	*SF4*	2.65E-54	**TG**	rs10401969	19	19407718	*SF4*	9.70E-70	1
**LDL-C**	rs12748152	1	27138393	*PIGV*	3.21E-12	**TG**	rs12748152	1	27138393	*PIGV*	1.10E-09	1
**LDL-C**	rs174546	11	61569830	*FADS1*	1.63E-39	**TG**	rs174546	11	61569830	*FADS1*	7.42E-38	1
**LDL-C**	rs2131925	1	63025942	*DOCK7*	2.98E-32	**TG**	rs2131925	1	63025942	*DOCK7*	3.05E-74	1
**LDL-C**	rs2954029	8	126490972	*TRIB1*	2.10E-50	**TG**	rs2954029	8	126490972	*TRIB1*	1.02E-107	1
**LDL-C**	rs3764261	16	56993324	*CETP*	2.22E-34	**TG**	rs3764261	16	56993324	*CETP*	1.58E-25	1
**LDL-C**	rs4722551	7	25991826	*MIR148A*	3.95E-14	**TG**	rs4722551	7	25991826	*MIR148A*	1.58E-09	1
**LDL-C**	rs6831256	4	3473139	*DOK7*	9.07E-07	**TG**	rs6831256	4	3473139	*DOK7*	1.60E-12	1
**LDL-C**	rs6882076	5	156390297	*TIMD4*	3.31E-31	**TG**	rs6882076	5	156390297	*TIMD4*	1.51E-15	1
**LDL-C**	rs964184	11	116648917	*ZNF259*	2.01E-26	**TG**	rs964184	11	116648917	*ZNF259*	6.55E-224	1

Supplementary Table 10: Location and association p-values for the SNPs associated with TG (Trait 1) that are in LD (r^2>0.8) with SNPs associated with the other traits (denoted as Trait 2). The table is sorted by Trait 2 and within Trait 2 by the SNP name of Trait 1.

Trait 1	SNP	Chr	Pos	Nearest gene	P-value	Trait 2	SNP	Chr	Pos	Nearest gene	P-value	R²
**TG**	rs1121980	16	53809247	*FTO*	1.52E-06	**BMI**	rs1558902	16	53803574	*FTO*	7.51E-153	0.956547
**TG**	rs11613352	12	57792580	*R3HDM2*	9.40E-14	**eGFR**	rs1106766	12	57809456	*R3HDM2*	4.70E-08	1
**TG**	rs1260326	2	27730940	*GCKR*	2.29E-239	**eGFR**	rs1260326	2	27730940	*GCKR*	3.40E-14	1
**TG**	rs1260326	2	27730940	*GCKR*	2.29E-239	**FG**	rs780094	2	27741237	*GCKR*	2.58E-37	0.911673
**TG**	rs174546	11	61569830	*FADS1*	7.42E-38	**FG**	rs174576	11	61603510	*FADS2*	1.18E-18	0.936276
**TG**	rs1121980	16	53809247	*FTO*	1.52E-06	**HDL-C**	rs1121980	16	53809247	*FTO*	6.79E-09	1
**TG**	rs11613352	12	57792580	*R3HDM2*	9.40E-14	**HDL-C**	rs11613352	12	57792580	*R3HDM2*	2.39E-13	1
**TG**	rs12678919	8	19844222	*LPL*	1.82E-199	**HDL-C**	rs12678919	8	19844222	*LPL*	1.38E-149	1
**TG**	rs12748152	1	27138393	*PIGV*	1.10E-09	**HDL-C**	rs12748152	1	27138393	*PIGV*	9.74E-16	1
**TG**	rs17145738	7	72982874	*TBL2*	9.42E-99	**HDL-C**	rs17145738	7	72982874	*TBL2*	4.95E-13	1
**TG**	rs174546	11	61569830	*FADS1*	7.42E-38	**HDL-C**	rs174546	11	61569830	*FADS1*	8.30E-28	1
**TG**	rs1936800	6	127436064	*RSPO3*	3.79E-07	**HDL-C**	rs1936800	6	127436064	*RSPO3*	3.06E-10	1
**TG**	rs2954029	8	126490972	*TRIB1*	1.02E-107	**HDL-C**	rs2954029	8	126490972	*TRIB1*	2.67E-29	1
**TG**	rs2972146	2	227100698	*IRS1*	2.97E-15	**HDL-C**	rs2972146	2	227100698	*IRS1*	1.85E-17	1
**TG**	rs3764261	16	56993324	*CETP*	1.58E-25	**HDL-C**	rs3764261	16	56993324	*CETP*	0	1
**TG**	rs4765127	12	124460167	*ZNF664*	2.41E-08	**HDL-C**	rs4765127	12	124460167	*ZNF664*	7.79E-10	1
**TG**	rs4846914	1	230295691	*GALNT2*	7.20E-31	**HDL-C**	rs4846914	1	230295691	*GALNT2*	3.51E-41	1
**TG**	rs6065906	20	44554015	*PCIF1*	2.36E-34	**HDL-C**	rs6065906	20	44554015	*PCIF1*	5.34E-40	1
**TG**	rs731839	19	33899065	*PEPD*	2.65E-09	**HDL-C**	rs731839	19	33899065	*PEPD*	3.44E-09	1
**TG**	rs838880	12	125261593	*SCARB1*	0.3064	**HDL-C**	rs838880	12	125261593	*SCARB1*	6.38E-32	1
**TG**	rs964184	11	116648917	*ZNF259*	6.55E-224	**HDL-C**	rs964184	11	116648917	*ZNF259*	6.09E-48	1
**TG**	rs998584	6	43757896	*VEGFA*	3.42E-15	**HDL-C**	rs998584	6	43757896	*VEGFA*	2.27E-11	1
**TG**	rs10401969	19	19407718	*SF4*	9.70E-70	**LDL-C**	rs10401969	19	19407718	*SF4*	2.65E-54	1
**TG**	rs12748152	1	27138393	*PIGV*	1.10E-09	**LDL-C**	rs12748152	1	27138393	*PIGV*	3.21E-12	1
**TG**	rs174546	11	61569830	*FADS1*	7.42E-38	**LDL-C**	rs174546	11	61569830	*FADS1*	1.63E-39	1
**TG**	rs2131925	1	63025942	*DOCK7*	3.05E-74	**LDL-C**	rs2131925	1	63025942	*DOCK7*	2.98E-32	1
**TG**	rs2954029	8	126490972	*TRIB1*	1.02E-107	**LDL-C**	rs2954029	8	126490972	*TRIB1*	2.10E-50	1
**TG**	rs3764261	16	56993324	*CETP*	1.58E-25	**LDL-C**	rs3764261	16	56993324	*CETP*	2.22E-34	1
**TG**	rs4722551	7	25991826	*MIR148A*	1.58E-09	**LDL-C**	rs4722551	7	25991826	*MIR148A*	3.95E-14	1

to be continued…

Supplementary Table 10 continued

Trait 1	SNP	Chr	Pos	Nearest gene	P-value	Trait 2	SNP	Chr	Pos	Nearest gene	P-value	R²
**TG**	rs6831256	4	3473139	*DOK7*	1.60E-12	**LDL-C**	rs6831256	4	3473139	*DOK7*	9.07E-07	1
**TG**	rs6882076	5	156390297	*TIMD4*	1.51E-15	**LDL-C**	rs6882076	5	156390297	*TIMD4*	3.31E-31	1
**TG**	rs964184	11	116648917	*ZNF259*	6.55E-224	**LDL-C**	rs964184	11	116648917	*ZNF259*	2.01E-26	1
**TG**	rs1936800	6	127436064	*RSPO3*	3.79E-07	**WHR**	rs1936805	6	127452116	*RSPO3*	8.40E-18	0.876715
**TG**	rs4765127	12	124460167	*ZNF664*	2.41E-08	**WHR**	rs4765219	12	124440110	*CCDC92*	9.40E-09	0.987213
**TG**	rs998584	6	43757896	*VEGFA*	3.42E-15	**WHR**	rs1358980	6	43764551	*VEGFA*	3.20E-14	0.829246

Supplementary Table 11: Location and association p-values for the SNPs associated with eGFR (Trait 1) that are in LD (r^2>0.8) with SNPs associated with the other traits (denoted as Trait 2). The table is sorted by Trait 2 and within Trait 2 by the SNP name of Trait 1.

Trait 1	SNP	Chr	Pos	Nearest gene	P-value	Trait 2	SNP	Chr	Pos	Nearest gene	P-value	R²
**eGFR**	rs10513801	3	185822353	*ETV5*	9.30E-08	**BMI**	rs1516725	3	185824004	*TRA2B*	1.89E-22	1
**eGFR**	rs1260326	2	27730940	*GCKR*	3.40E-14	**Fasting Glucose**	rs780094	2	27741237	*GCKR*	2.58E-37	0.911673
**eGFR**	rs7422339	2	211540507	*CPS1*	2.20E-23	**HDL-C**	rs1047891	2	211540507	*CPS1*	8.73E-10	1
**eGFR**	rs1106766	12	57809456	*R3HDM2*	4.70E-08	**HDL-C**	rs11613352	12	57792580	*R3HDM2*	2.39E-13	1
**eGFR**	rs1106766	12	57809456	*R3HDM2*	4.70E-08	**TG**	rs11613352	12	57792580	*R3HDM2*	9.40E-14	1
**eGFR**	rs1260326	2	27730940	*GCKR*	3.40E-14	**TG**	rs1260326	2	27730940	*GCKR*	2.29E-239	1

Supplementary Table 12: Location and association p-values for the SNPs associated with BMI (Trait 1) that are in LD (r^2>0.8) with SNPs associated with the other traits (denoted as Trait 2). The table is sorted by Trait 2 and within Trait 2 by the SNP name of Trait 1.

Trait 1	SNP	Chr	Pos	Nearest gene	P-value	Trait 2	SNP	Chr	Pos	Nearest gene	P-value	R^2
**BMI**	rs1516725	3	185824004	TRA2B	1.89E-22	**eGFR**	rs10513801	3	185822353	ETV5	9.30E-08	1
**BMI**	rs7903146	10	114758349	TCF7L2	1.11E-11	**Fasting Glucose**	rs7903146	10	114758349	TCF7L2	2.71E-20	1
**BMI**	rs13107325	4	103188709	SLC39A8	1.83E-12	**HDL-C**	rs13107325	4	103188709	SLC39A8	1.07E-15	1
**BMI**	rs1558902	16	53803574	FTO	7.51E-153	**HDL-C**	rs1121980	16	53809247	FTO	6.79E-09	0.956547
**BMI**	rs6567160	18	57829135	MC4R	3.93E-53	**HDL-C**	rs12967135	18	57849023	MC4R	3.57E-08	1
**BMI**	rs1558902	16	53803574	FTO	7.51E-153	**TG**	rs1121980	16	53809247	FTO	1.52E-06	0.956547

Supplementary Table 13: Location and association p-values for the SNPs associated with WHR (Trait 1) that are in LD (r^2>0.8) with SNPs associated with the other traits (denoted as Trait 2). The table is sorted by Trait 2 and within Trait 2 by the SNP name of Trait 1.

Trait 1	SNP	Chr	Pos	Nearest gene	P-value	Trait 2	SNP	Chr	Pos	Nearest gene	P-value	R^2
**WHR**	rs1358980	6	43764551	*VEGFA*	3.20E-14	**HDL-C**	rs998584	6	43757896	*VEGFA*	2.27E-11	0.829246
**WHR**	rs1936805	6	127452116	*RSPO3*	8.40E-18	**HDL-C**	rs1936800	6	127436064	*RSPO3*	3.06E-10	0.876715
**WHR**	rs4765219	12	124440110	*CCDC92*	9.40E-09	**HDL-C**	rs4765127	12	124460167	*ZNF664*	7.79E-10	0.987213
**WHR**	rs9991328	4	89713121	*FAM13A*	0.089	**HDL-C**	rs3822072	4	89741269	*FAM13A*	4.06E-12	0.960722
**WHR**	rs1358980	6	43764551	*VEGFA*	3.20E-14	**TG**	rs998584	6	43757896	*VEGFA*	3.42E-15	0.829246
**WHR**	rs1936805	6	127452116	*RSPO3*	8.40E-18	**TG**	rs1936800	6	127436064	*RSPO3*	3.79E-07	0.876715
**WHR**	rs4765219	12	124440110	*CCDC92*	9.40E-09	**TG**	rs4765127	12	124460167	*ZNF664*	2.41E-08	0.987213

Supplementary Table 14: Location and association p-values for the SNPs associated with fasting glucose (Trait 1) that are in LD (r^2>0.8) with SNPs associated with the other traits (denoted as Trait 2). The table is sorted by Trait 2 and within Trait 2 by the SNP name of Trait 1.

Trait 1	SNP	Chr	Pos	Nearest gene	P-value	Trait 2	SNP	Chr	Pos	Nearest gene	P-value	R^2
**Fasting Glucose**	rs7903146	10	114758349	*TCF7L2*	2.71E-20	**BMI**	rs7903146	10	114758349	*TCF7L2*	1.11E-11	1
**Fasting Glucose**	rs780094	2	27741237	*GCKR*	2.58E-37	**eGFR**	rs1260326	2	27730940	*GCKR*	3.40E-14	0.911673
**Fasting Glucose**	rs174576	11	61603510	*FADS2*	1.18E-18	**HDL-C**	rs174546	11	61569830	*FADS1*	8.30E-28	0.936276
**Fasting Glucose**	rs174576	11	61603510	*FADS2*	1.18E-18	**LDL-C**	rs174546	11	61569830	*FADS1*	1.63E-39	0.936276
**Fasting Glucose**	rs174576	11	61603510	*FADS2*	1.18E-18	**TG**	rs174546	11	61569830	*FADS1*	7.42E-38	0.936276
**Fasting Glucose**	rs780094	2	27741237	*GCKR*	2.58E-37	**TG**	rs1260326	2	27730940	*GCKR*	2.29E-239	0.911673

Supplementary Table 15: Causal effect estimates of lipid traits, adiposity-related traits and fasting glucose on apoA-IV concentrations, contrasting the results from the conventional Mendelian randomization approach (MR-IVW) with those from the sensitivity analyses using the proxy search approach.

	Causal effect of phenotype on apoA-IV							
	**All SNPs**				**Without potentially pleiotropic SNPs (proxy search**)**			
**Phenotype (exposure)**	**No. of SNPs**	**Beta***	**95% CI***	**P-value**	**No. of SNPs**	**Beta***	**95% CI***	**P-value**
Lipid traits								
HDL-C, unadjusted	71	0.0341	[0.0142, 0.0540]	**0.0008**	48	0.0324	[0.0007, 0.0641]	0.0453
HDL-C, adjusted for LDL-C and TG (multivariable MR)	71	0.0195	[-0.0084, 0.0475]	0.1706	61[§]	0.0224	[-0.0059, 0.0507]	0.1212
LDL-C, unadjusted	58	-0.0376	[-0.0572, -0.0179]	**0.0002**	46	-0.0293	[-0.0528, -0.0058]	0.0144
LDL-C, adjusted for LDL-C and TG (multivariable MR)	58	-0.0225	[-0.0441, -0.0010]	0.0406	57[§]	-0.0234	[-0.0457, -0.0011]	0.0395
TG, unadjusted	40	-0.0600	[-0.0834, -0.0366]	**4.8e-07**	17	-0.0576	[-0.1262, 0.0111]	0.1003
TG, adjusted for HDL-C and LDL-C (multivariable MR)	40	-0.0597	[-0.0962, -0.0232]	**0.0014**	33[§]	-0.0625	[-0.1057, -0.0192]	**0.0047**
Kidney function								
eGFR	53	-0.3890	[-0.5367, -0.2413]	**2.4e-07**	49	-0.3987	[-0.5528, -0.2447]	**3.9e-07**
Adiposity-related parameters								
BMI	77	0.0067	[-0.0266, 0.0400]	0.6946	72	0.0179	[-0.0201, 0.0560]	0.3558
WHR	40	0.0761	[0.0093, 0.1429]	0.0255	36	0.0745	[0.0017, 0.1473]	0.0450
Fasting Glucose								
Fasting Glucose	36	0.0146	[-0.0364, 0.0656]	0.5742	33	-0.0047	[-0.0578, 0.0483]	0.8613

*in log(apoA-IV) per change in SD for the lipid and obesity traits, in log(apoA-IV) per change in log(eGFR) for kidney function, in log(apoA-IV) per change in mmol/L for fasting glucose.
**The search for proxies was performed using SNiPA (http://snipa.helmholtz-muenchen.de/snipa/index.php?task=proxy_search) with an LD threshold of 0.8.
[§]Removing all SNPs included in the SNP set or in LD with any SNP in the SNP set of the non-lipid traits.

Supplementary Table 16: Causal effect estimates of lipid traits, adiposity-related traits and fasting glucose on apoA-IV concentrations, contrasting the results from the conventional Mendelian randomization approach (MR-IVW) with those from sensitivity analyses (MR-Egger regression and weighted median estimation).

Phenotype	No. of SNPs	Beta*	95% CI*	P-value
**HDL-C**				
Main analysis:				
MR-IVW	71	0.0341	[0.0142, 0.0540]	**0.0008**
Sensitivity analyses:				
1) MR-Egger regression	71	0.0611	[0.0254, 0.0969]	**0.0008**
2) Weighted median estimation	71	0.0193	[-0.0129, 0.0516]	0.2405
3) MR-IVW excluding rs964184**	70	0.0271	[0.0067, 0.0474]	0.0091
**LDL-C**				
Main analysis:				
MR-IVW	58	-0.0376	[-0.0572, -0.0179]	**0.0002**
Sensitivity analyses:				
1) MR-Egger regression	58	-0.0555	[-0.0910, -0.0200]	**0.0022**
2) Weighted median estimation	58	-0.0427	[-0.0733, -0.0121]	**0.0062**
3) MR-IVW excluding rs964184	57	-0.0320	[-0.0519, -0.0121]	**0.0016**
**TG**				
Main analysis:				
MR-IVW	40	-0.0600	[-0.0834, -0.0366]	**4.8e-07**
Sensitivity analyses:				
1) MR-Egger regression	40	-0.0709	[-0.1075, -0.0343]	**0.0001**
2) Weighted median estimation	40	-0.0600	[-0.0926, -0.0274]	**0.0003**
3) MR-IVW excluding rs964184	39	-0.0498	[-0.0776, -0.0220]	**0.0004**
**eGFR**				
Main analysis:				
MR-IVW	53	-0.3890	[-0.5367, -0.2413]	**2.4e-07**
Sensitivity analyses:				
1) MR-Egger regression	53	-0.6694	[-1.1460, -0.1928]	**0.0059**
2) Weighted median estimation	53	-0.4894	[-0.7116, -0.2671]	**1.59e-05**
**BMI**				
Main analysis:				
MR-IVW	77	0.0067	[-0.0266, 0.0400]	0.6946
Sensitivity analyses:				
1) MR-Egger regression	77	-0.0079	[-0.0910, 0.0753]	0.8532
2) Weighted median estimation	77	-0.0196	[-0.0730, 0.0337]	0.4705
**WHR**				
Main analysis:				
MR-IVW	40	0.0761	[0.0093, 0.1429]	0.0255
Sensitivity analyses:				
1) MR-Egger regression	40	0.1025	[-0.0519, 0.2570]	0.1933
2) Weighted median estimation	40	0.0735	[-0.0253, 0.1723]	0.1446
**Fasting Glucose**				
Main analysis:				
MR-IVW	36	0.0146	[-0.0364, 0.0656]	0.5742
Sensitivity analyses:				
1) MR-Egger regression	36	-0.0439	[-0.1517, 0.0639]	0.4246
2) Weighted median estimation	36	-0.0214	[-0.0966, 0.0539]	0.5775

* in log(apoA-IV) per change in SD for the lipid and obesity traits, in log(apoA-IV) per change in log(eGFR) for kidney function, in log(apoA-IV) per change in mmol/L for fasting glucose.

** SNP excluded based on the gtx-package used for testing presence of pleiotropy.

Bold: significant after Bonferroni correction (α=0.00625).

Supplementary Table 17: Intercept estimates of MR-Egger regressions for detection of directional pleiotropy of the genetic instruments associated with lipid traits, kidney function, adiposity-related traits and fasting glucose.

Phenotype	No. of SNPs	MR-Egger intercept estimate*	95% CI*	P-value
HDL-C	71	-0.0018	[-0.0036, 1.7e-05]	0.0522
LDL-C	58	0.0013	[-0.0008, 0.0035]	0.2203
TG	40	0.0008	[-0.0012, 0.0029]	0.4276
eGFR	53	0.0023	[-0.0014, 0.0060]	0.2253
BMI	77	0.0005	[-0.0019, 0.0028]	0.7074
WHR	40	-0.0006	[-0.0036, 0.0024]	0.7076
Fasting Glucose	36	0.0020	[-0.0011, 0.0050]	0.2035

*in log(apoA-IV) per change in SD for the lipid and obesity traits, in log(apoA-IV) per change in log(eGFR) for kidney function, in log(apoA-IV) per change in mmol/L for fasting glucose.

Supplementary Table 18: Adapted I²-statistic ($I^2{}_{GX}$) and the corrected MR-Egger estimates based on the method of Simulation Extrapolation (SIMEX). Here, apoA-IV was considered as the outcome and the phenotypes were considered as the exposure.

		Unadjusted MR-Egger estimate			Adjusted MR-Egger estimate (SIMEX)		
Phenotype	$I^2{}_{GX}$	Beta*	95% CI*	P-value	Beta*	95% CI*	P-value
HDL-C	0.9846	0.0611	[0.0254, 0.0969]	**0.0008**	0.0621	[0.0251, 0.0990]	**0.0013**
LDL-C	0.9817	-0.0555	[-0.0910, -0.0200]	**0.0022**	-0.0564	[-0.0933, -0.0194]	**0.0035**
TG	0.9865	-0.0709	[-0.1075, -0.0343]	**0.0001**	-0.0718	[-0.1101, -0.0335]	**0.0005**
eGFR	0.8213	-0.6694	[-1.1460, -0.1928]	**0.0059**	-0.7973	[-1.3370, -0.2576]	**0.0046**
BMI	0.9072	-0.0079	[-0.0910, 0.0753]	0.8532	-0.0089	[-0.1007, 0.0829]	0.8478
WHR	0.8105	0.1025	[-0.0519, 0.2570]	0.1933	0.1230	[-0.0637, 0.3096]	0.1902
Fasting Glucose	0.9723	-0.0439	[-0.1517, 0.0639]	0.4246	-0.0446	[-0.1585, 0.0692]	0.4314

*in log(apoA-IV) per change in SD for the lipid and obesity traits, in log(apoA-IV) per change in log(eGFR) for kidney function, in log(apoA-IV) per change in mmol/L for fasting glucose.

Bold: significant after Bonferroni correction (α=0.00625).

Supplementary Table 19: Characteristics and meta-analysis results of the 3 SNPs associated with apoA-IV [8].

					Results from the GWAS meta-analysis on							
					**apoA-IV**		**HDL-C**		**LDL-C**		**TG**	
**SNP**	**Chr**	**Pos**	**Nearest Gene**	**A1/ A2[#]**	**Beta* (SE*)**	**P-value**	**Beta* (SE*)**	**P-value**	**Beta* (SE*)**	**P-value**	**Beta* (SE*)**	**P-value**
rs1729407	11	116677370	*APOA4*	G/ C	-0.0459 (0.0033)	**6.77e-44**	0.0233 (0.0054)	**7.06e-07**	-0.0061 (0.0059)	0.4332	-0.0015 (0.0053)	0.9608
rs5104[§]	11	116684028	*APOA4*	C/ T	-0.0479 (0.0047)	**1.79e-24**						
rs4253311	4	187174683	*KLKB1*	G/ A	0.0256 (0.0034)	**5.63e-14**	-0.0034 (0.0048)	0.5394	0.0088 (0.0052)	0.0894	-0.0090 (0.0047)	0.0677

					Results from the GWAS meta-analysis on							
					**eGFR**		**BMI**		**WHR**		**Fasting Glucose**	
**SNP**	**Chr**	**Pos**	**Nearest Gene**	**A1/ A2[#]**	**Beta* (SE*)**	**P-value**	**Beta* (SE*)**	**P-value**	**Beta* (SE*)**	**P-value**	**Beta* (SE*)**	**P-value**
rs1729407	11	116677370	*APOA4*	G/ C	-0.0008 (0.0009)	0.4200	0.0046 (0.0042)	0.2734	0.0028 (0.0048)	0.5600	-0.0037 (0.0034)	0.2760
rs5104[§]	11	116684028	*APOA4*	C/ T							0.0087 (0.8317)	8.7e-03
rs4253311	4	187174683	*KLKB1*	G/ A	0.0008 (0.0009)	0.3500	0.0035 (0.0037)	0.3442	0.0049 (0.0042)	0.2400	0.0031 (0.9359)	3.1e-03

[#]A1: effect allele; A2: other allele.

*in log(apoA-IV) for apoA-IV, in SD for the lipid and obesity traits, in log(eGFR) for kidney function, in mmol/L for fasting glucose.

[§]rs5104 was not present in any of the other GWAs consortia for the other traits. For fasting glucose, the results from one proxy SNP (rs7396835, r^2=0.95 with rs5104) could be retrieved. For the other phenotypes no proxies for SNP rs5104 were present in the datasets.

Bold: significant after Bonferroni correction (α=0.00625).

Supplementary Figures

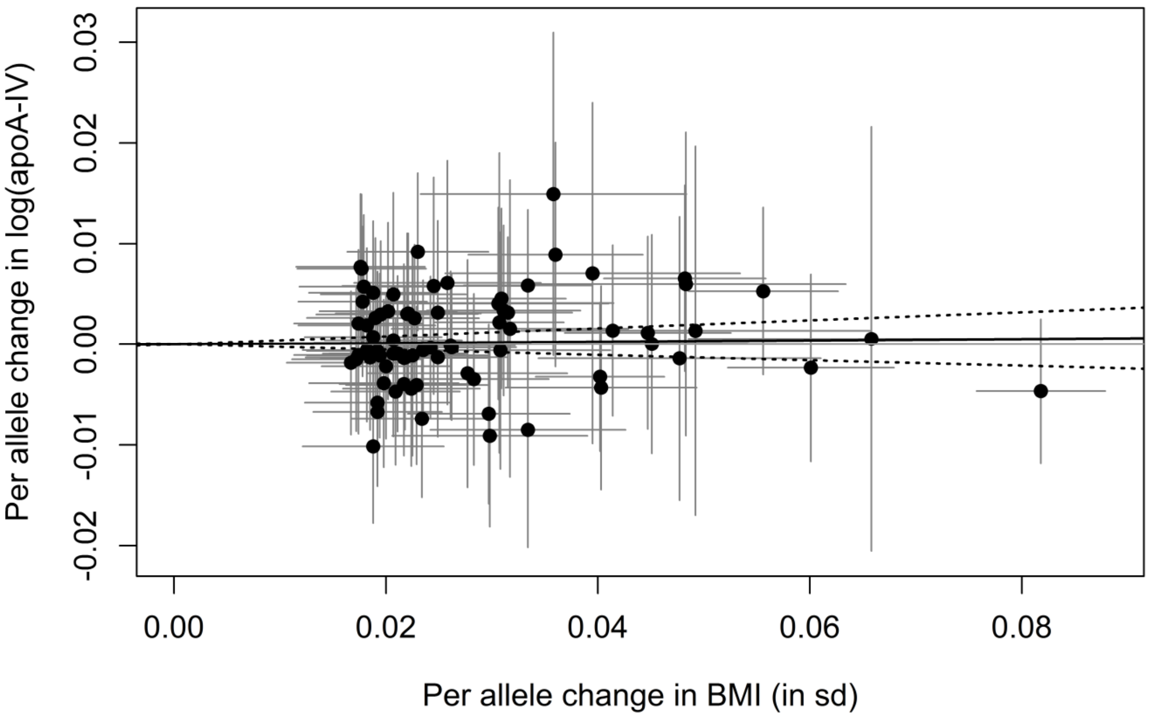

Supplementary Figure 1: Scatterplot showing the effect estimates of SNP-BMI (body-mass index) associations (95% CI) on the x- and SNP-apoA-IV (apolipoprotein A-IV) associations (95% CI) on the y-axis for all 77 SNPs. The continuous black line represents the MR-IVW estimate of BMI on apoA-IV (dashed lines represent the 95% CI).

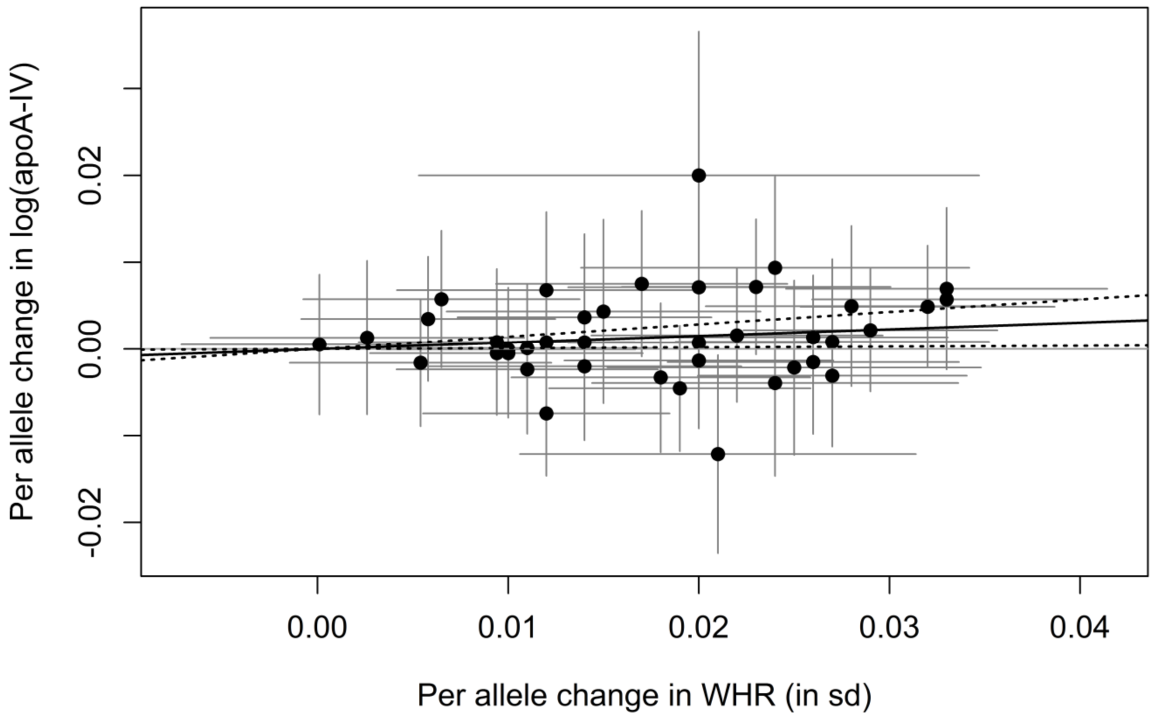

Supplementary Figure 2: Scatterplot showing the effect estimates of SNP-WHR (waist-hip-ratio) associations (95% CI) on the x- and SNP-apoA-IV (apolipoprotein A-IV) associations (95% CI) on the y-axis for all 40 SNPs. The continuous black line represents the MR-IVW estimate of WHR on apoA-IV (dashed lines represent the 95% CI).

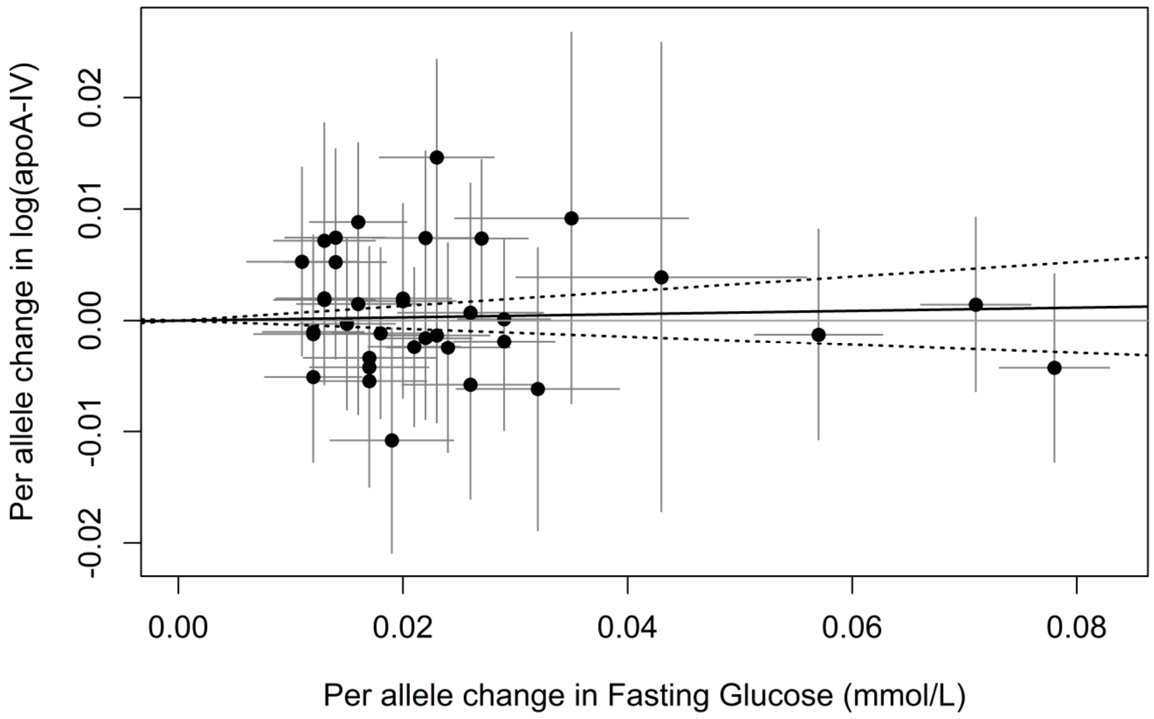

Supplementary Figure 3: Scatterplot showing the effect estimates of SNP-Fasting Glucose associations (95% CI) on the x- and SNP-apoA-IV (apolipoprotein A-IV) associations (95% CI) on the y-axis for all 36 SNPs. The continuous black line represents the MR-IVW estimate of Fasting Glucose on apoA-IV (dashed lines represent the 95% CI).

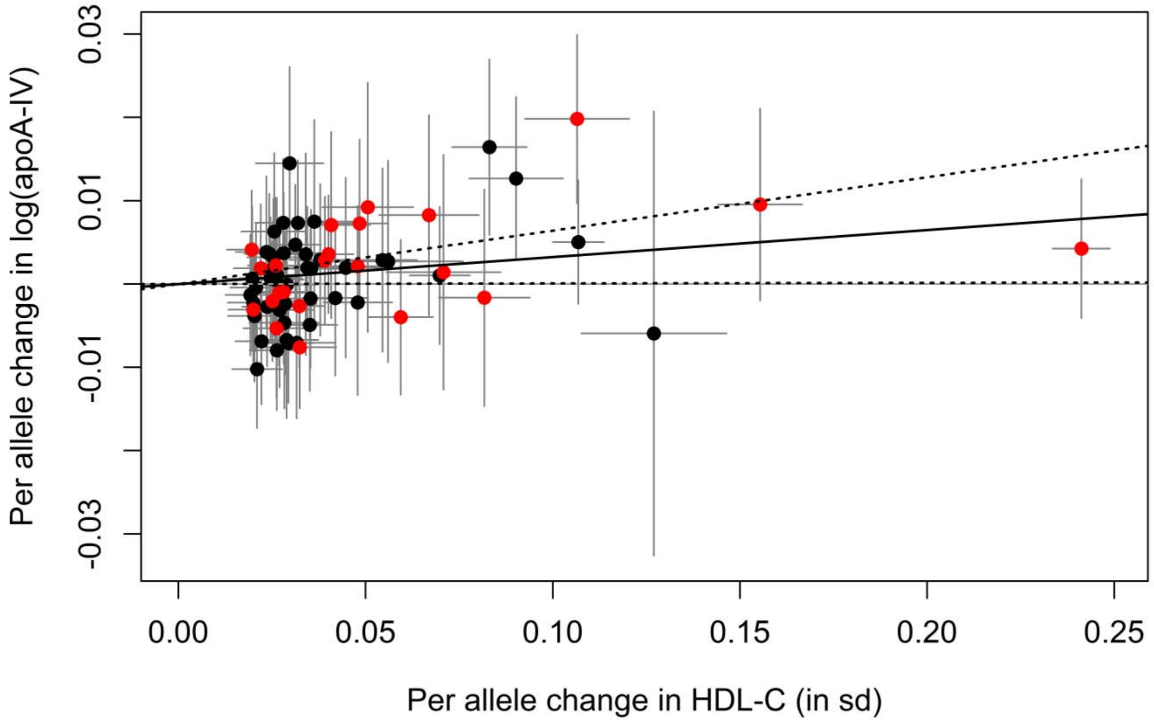

Supplementary Figure 4: Scatterplot showing the effect estimates of SNP-HDL-C associations (95% CI) on the x- and SNP-apoA-IV (apolipoprotein A-IV) associations (95% CI) on the y-axis for all 71 SNPs. Red points indicate the 23 SNPs that were in LD with SNPs associated with any of the other traits. The continuous black line represents the MR-IVW estimate of HDL-C on apoA-IV based on the 48 SNPs not associated with any other trait (dashed lines represent the 95% CI).

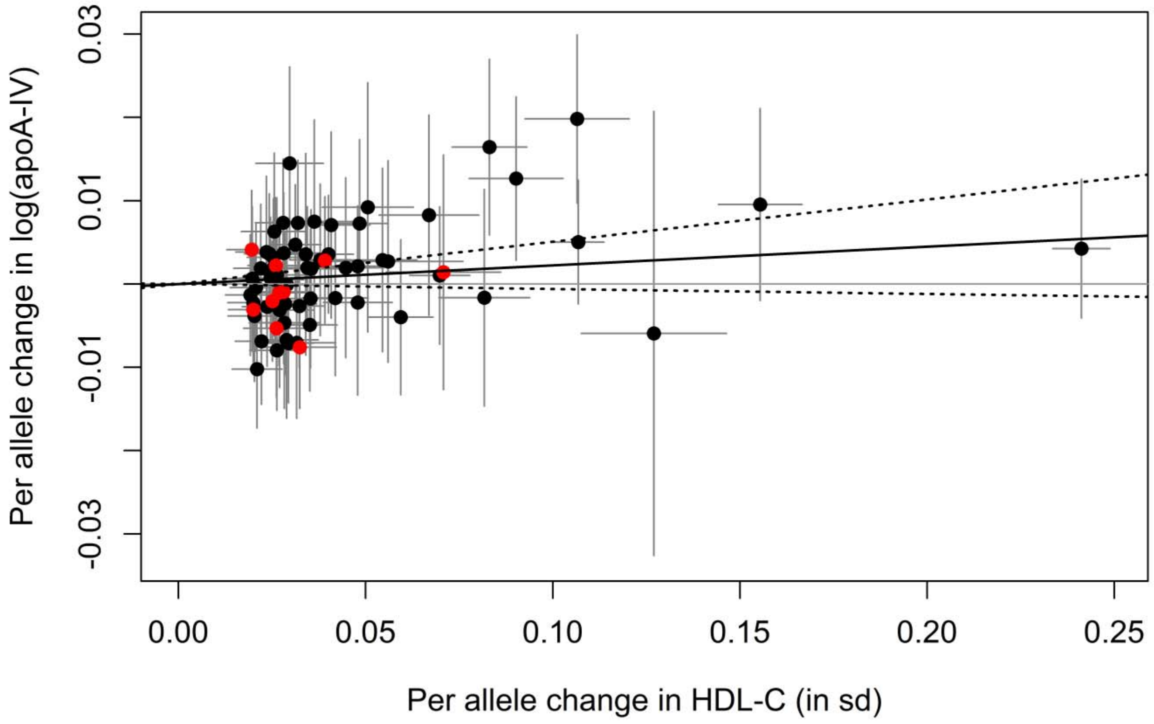

Supplementary Figure 5: Scatterplot showing the effect estimates of SNP-HDL-C associations (95% CI) on the x- and SNP-apoA-IV (apolipoprotein A-IV) associations (95% CI) on the y-axis for all 71 SNPs. Red points indicate the 10 SNPs that were in LD with SNPs associated with the non-lipid traits. The continuous black line represents the causal estimate of HDL-C on apoA-IV after application of the multivariable Mendelian randomization approach based on the 61 SNPs not associated with any non-lipid-trait (dashed lines represent the 95% CI).

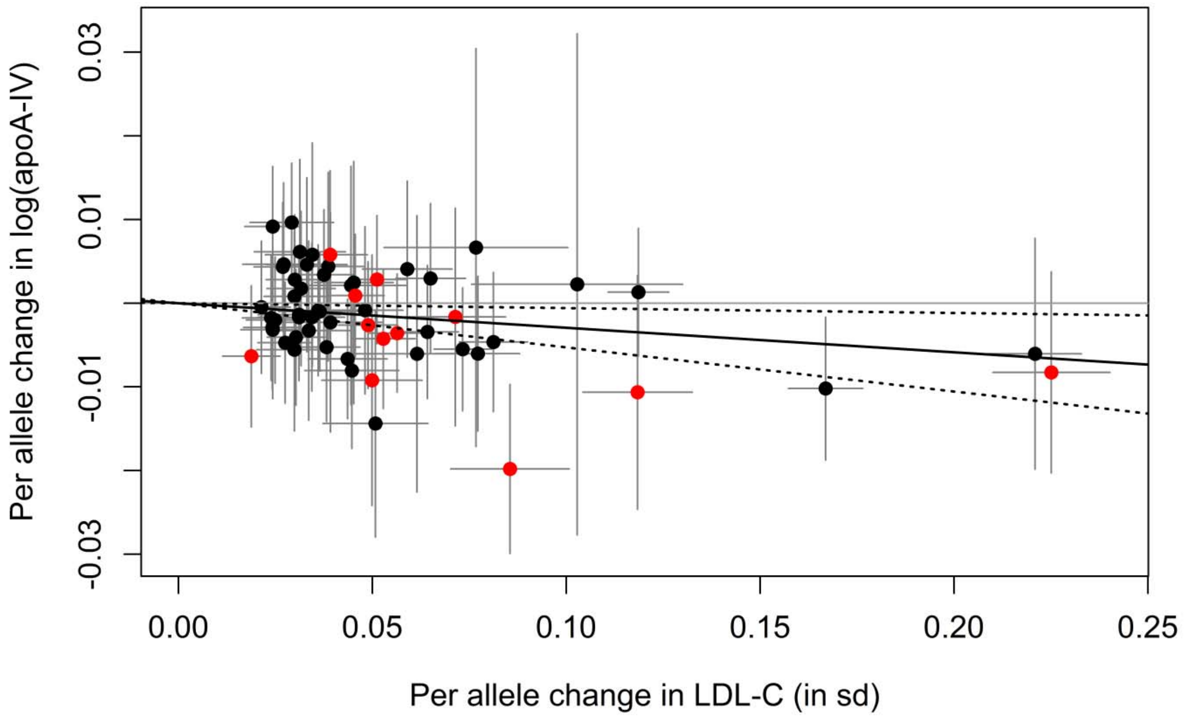

Supplementary Figure 6: Scatterplot showing the effect estimates of SNP-LDL-C associations (95% CI) on the x- and SNP-apoA-IV (apolipoprotein A-IV) associations (95% CI) on the y-axis for all 58 SNPs. Red points indicate the 12 SNPs that were in LD with SNPs associated with any of the other traits. The continuous black line represents the MR-IVW estimate of LDL-C on apoA-IV based on the 46 SNPs not associated with any other trait (dashed lines represent the 95% CI).

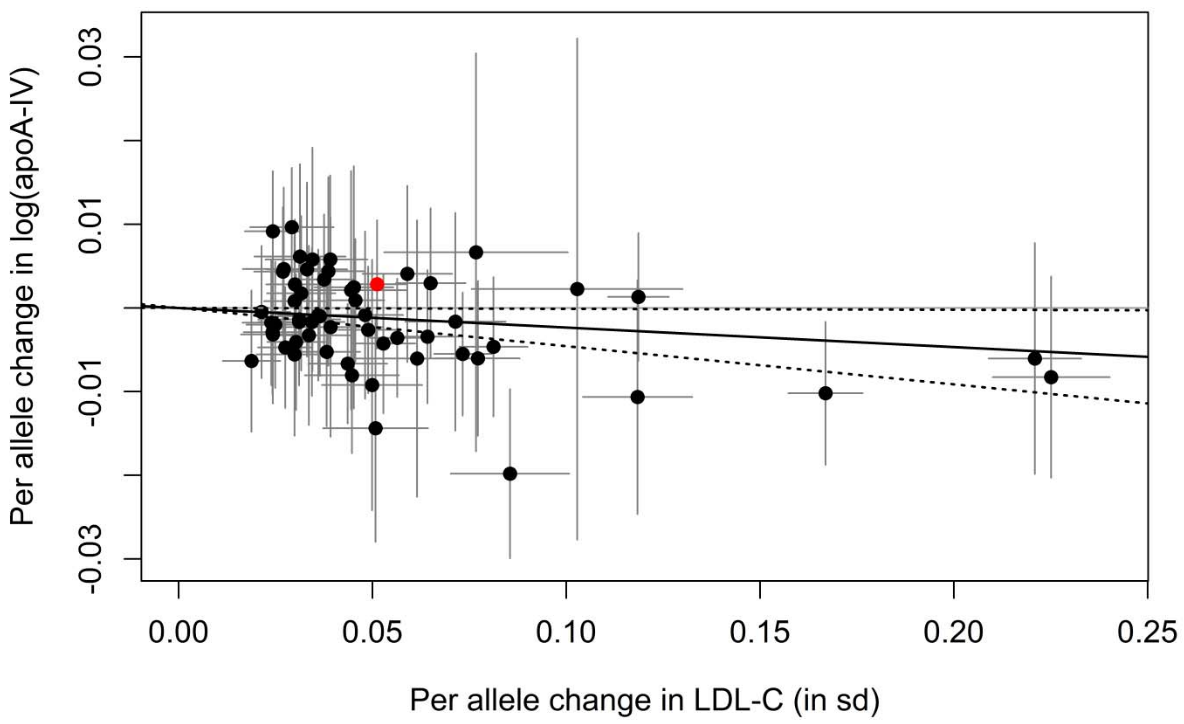

Supplementary Figure 7: Scatterplot showing the effect estimates of SNP-LDL-C associations (95% CI) on the x- and SNP-apoA-IV (apolipoprotein A-IV) associations (95% CI) on the y-axis for all 58 SNPs. The red point indicates the one SNP that was in LD with SNPs associated with the non-lipid traits. The continuous black line represents the causal estimate of LDL-C on apoA-IV after application of the multivariable Mendelian randomization approach based on the 57 SNPs not associated with any non-lipid-trait (dashed lines represent the 95% CI).

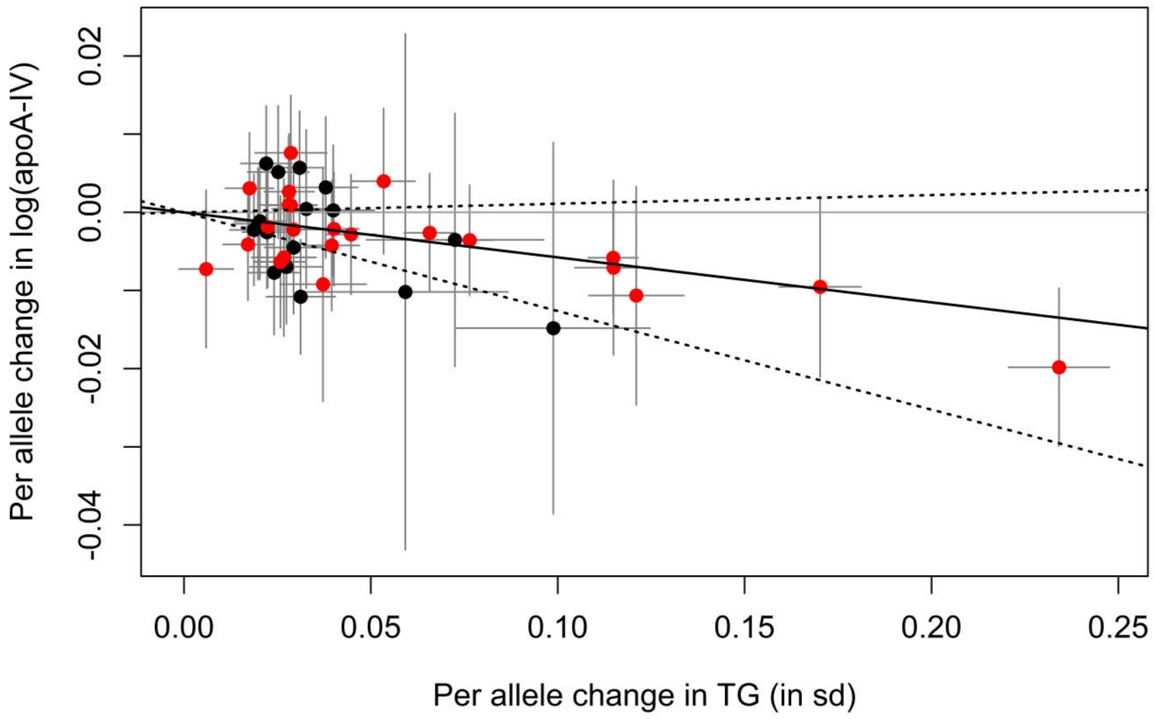

Supplementary Figure 8: Scatterplot showing the effect estimates of SNP-TG associations (95% CI) on the x- and SNP-apoA-IV (apolipoprotein A-IV) associations (95% CI) on the y-axis for all 40 SNPs. Red points indicate the 23 SNPs that were in LD with SNPs associated with any of the other traits. The continuous black line represents the MR-IVW estimate of TG on apoA-IV based on the 17 SNPs not associated with any other trait (dashed lines represent the 95% CI).

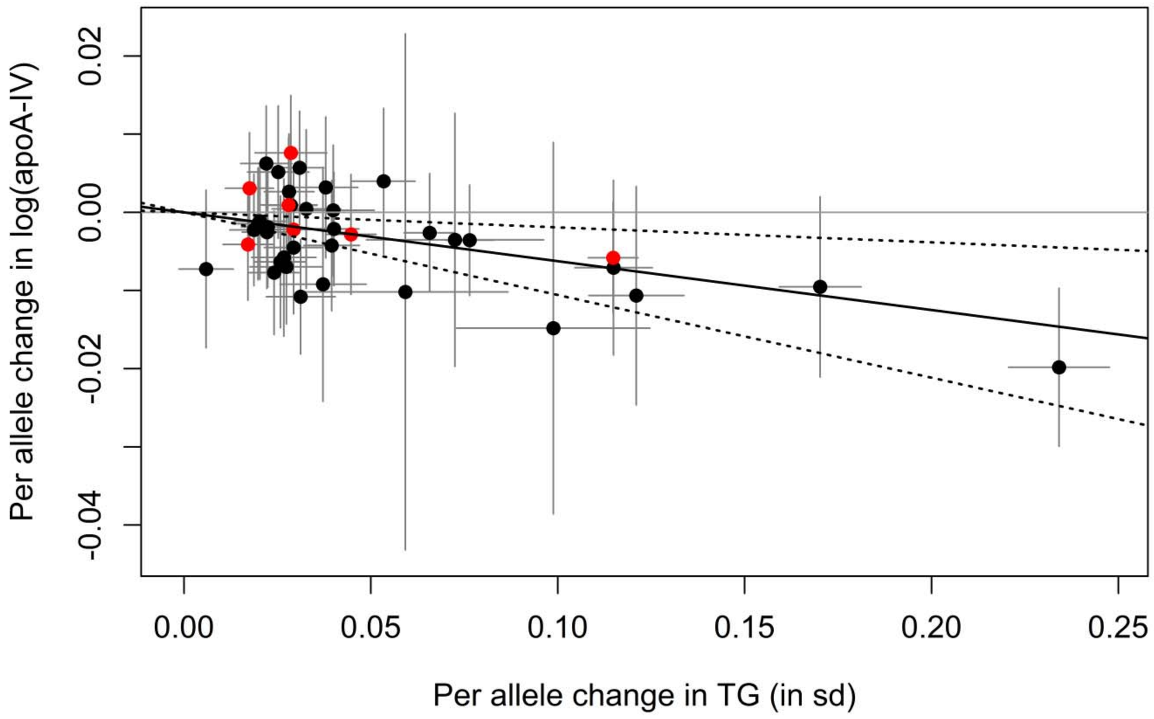

Supplementary Figure 9: Scatterplot showing the effect estimates of SNP-TG associations (95% CI) on the x- and SNP-apoA-IV (apolipoprotein A-IV) associations (95% CI) on the y-axis for all 40 SNPs. Red points indicate the 7 SNPs that were in LD with SNPs associated with the non-lipid traits. The continuous black line represents the causal estimate of TG on apoA-IV after application of the multivariable Mendelian randomization approach based on the 33 SNPs not associated with any non-lipid-trait (dashed lines represent the 95% CI).

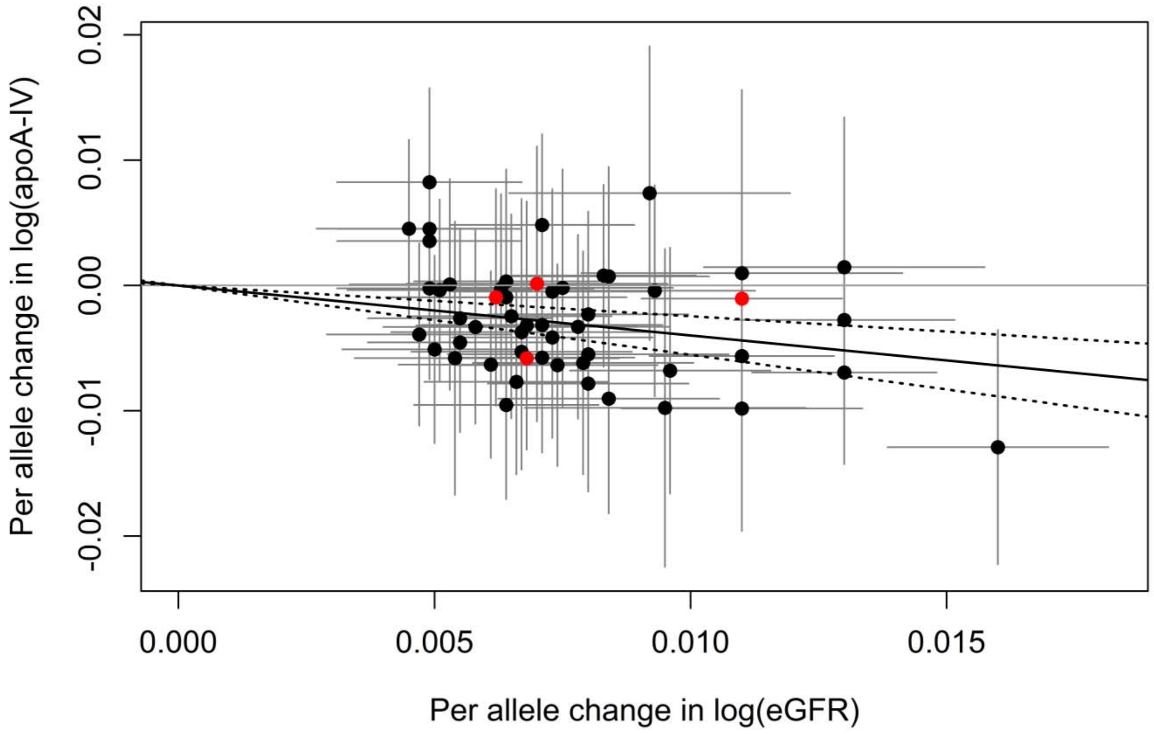

Supplementary Figure 10: Scatterplot showing the effect estimates of SNP-eGFR associations (95% CI) on the x- and SNP-apoA-IV (apolipoprotein A-IV) associations (95% CI) on the y-axis for all 53 SNPs. Red points indicate the 4 SNPs that were in LD with SNPs associated with any of the other traits. The continuous black line represents the MR-IVW estimate of eGFR on apoA-IV based on the 49 SNPs not associated with any other trait (dashed lines represent the 95% CI).

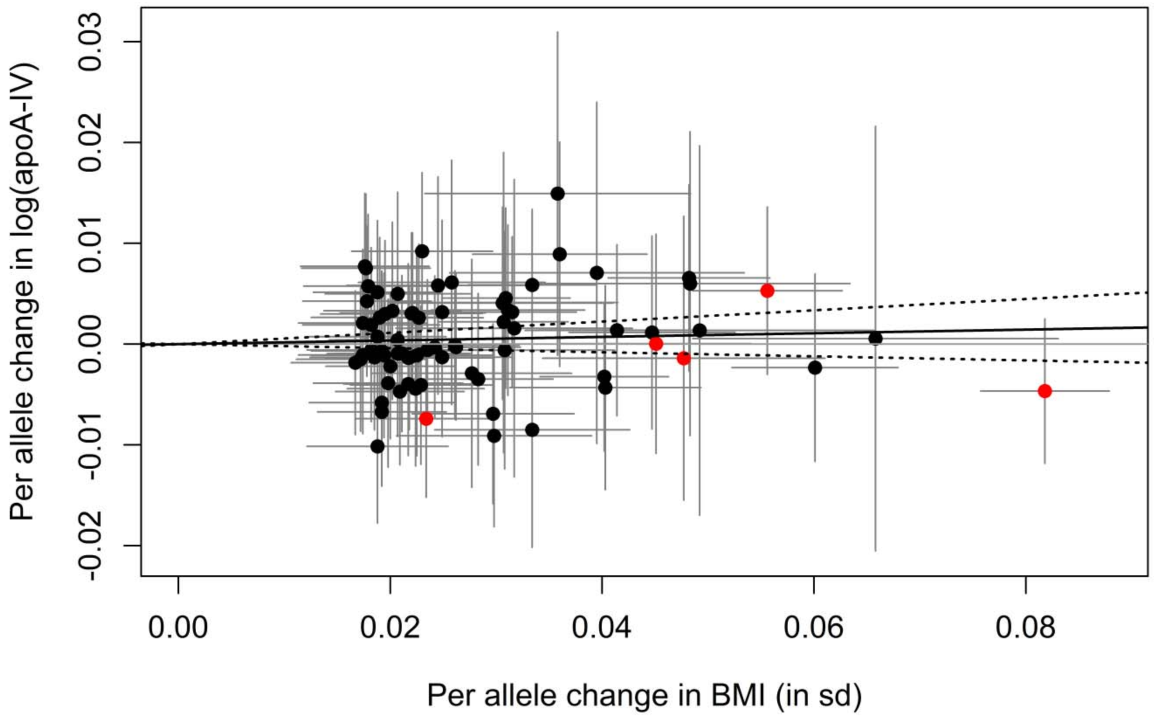

Supplementary Figure 11: Scatterplot showing the effect estimates of SNP-BMI associations (95% CI) on the x- and SNP-apoA-IV (apolipoprotein A-IV) associations (95% CI) on the y-axis for all 77 SNPs. Red points indicate the 5 SNPs that were in LD with SNPs associated with any of the other traits. The continuous black line represents the MR-IVW estimate of BMI on apoA-IV based on the 72 SNPs not associated with any other trait (dashed lines represent the 95% CI).

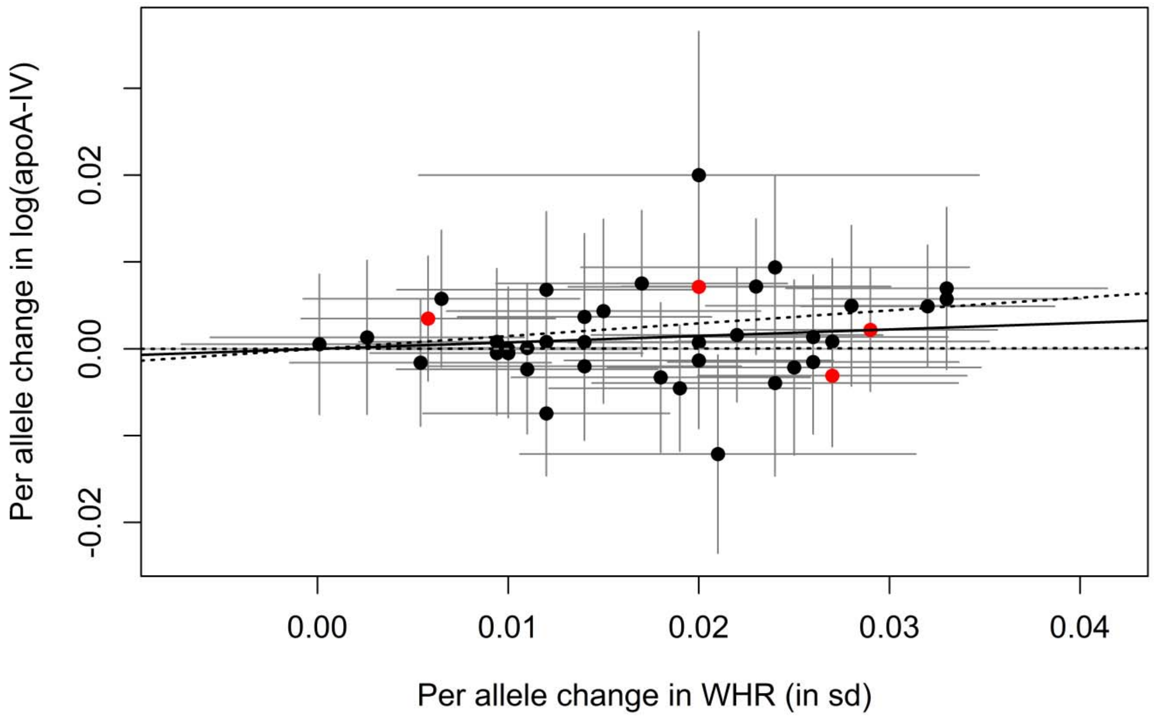

Supplementary Figure 12: Scatterplot showing the effect estimates of SNP-WHR associations (95% CI) on the x- and SNP-apoA-IV (apolipoprotein A-IV) associations (95% CI) on the y-axis for all 40 SNPs. Red points indicate the 4 SNPs that were in LD with SNPs associated with any of the other traits. The continuous black line represents the MR-IVW estimate of WHR on apoA-IV based on the 36 SNPs not associated with any other trait (dashed lines represent the 95% CI).

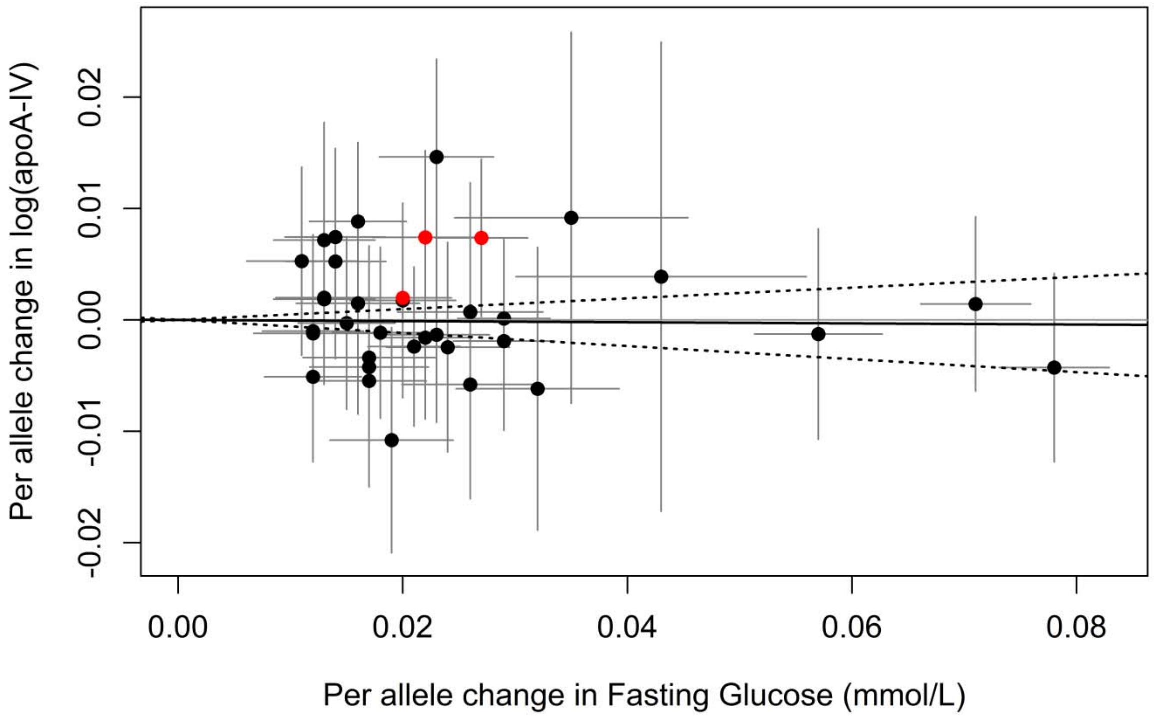

Supplementary Figure 13: Scatterplot showing the effect estimates of SNP-Fasting Glucose associations (95% CI) on the x- and SNP-apoA-IV (apolipoprotein A-IV) associations (95% CI) on the y-axis for all 36 SNPs. Red points indicate the 3 SNPs that were in LD with SNPs associated with any of the other traits. The continuous black line represents the MR-IVW estimate of Fasting Glucose on apoA-IV based on the 33 SNPs not associated with any other trait (dashed lines represent the 95% CI).

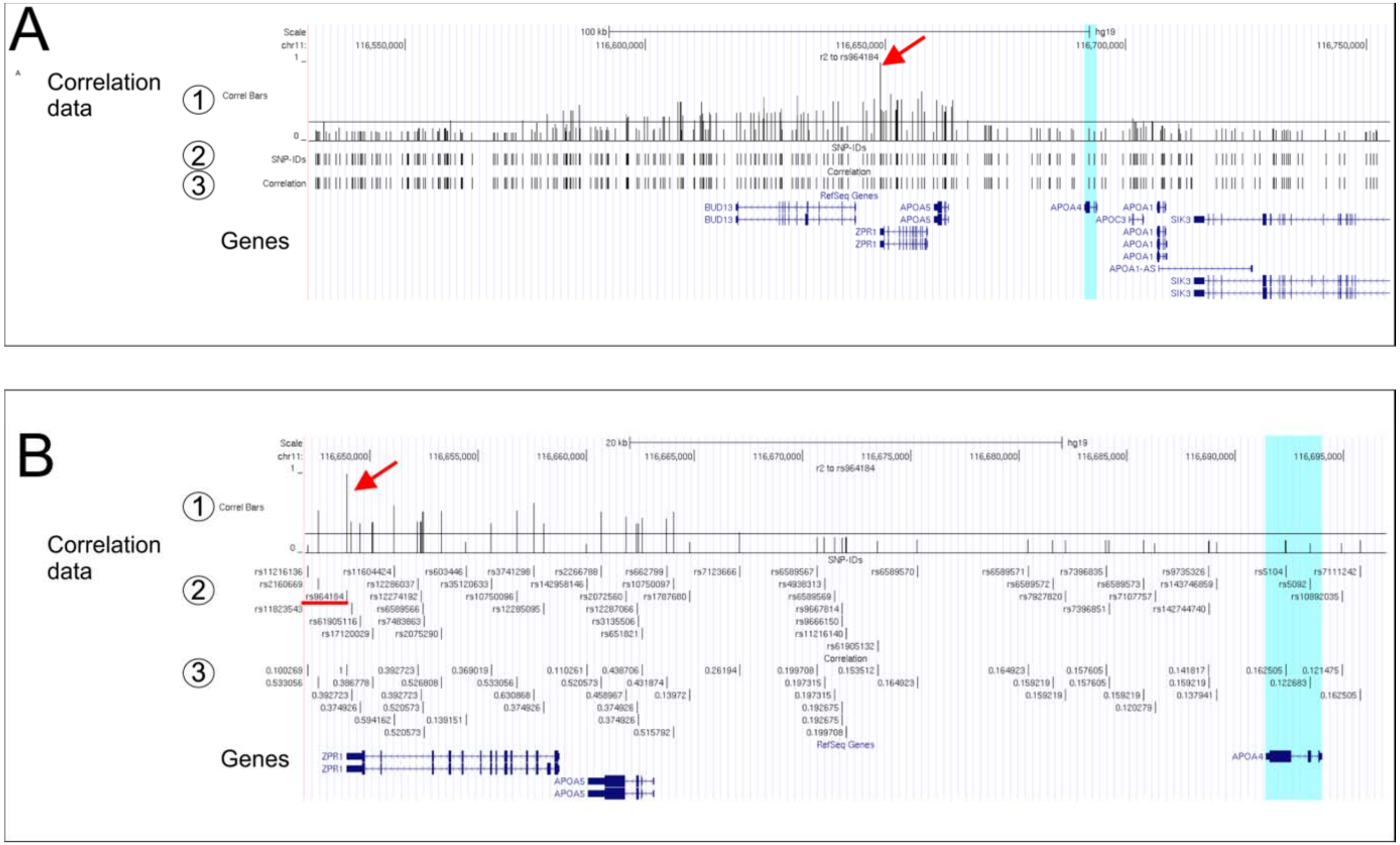

Supplementary Figure 14: Reported correlation of SNPs in the *APOA5-A4-C3-A1* gene cluster with rs964184 (given as r^2 as reported by SNiPA using 1000 Genomes phase 3v5 data). *APOA4* gene is highlighted. **A.** Whole genomic region (225 kb; chr11:116,530,000-116,755,000, hg19). **B.** Close-up view of region spanning *ZPR1, APOA5* and *APOA4.*

Both panels. Row 1: bar plot showing the r^2 value with rs964184 for the SNPs reported in row 1. The solid horizontal lines delines r^2=0.25 level. Row 2: rs-IDs of the SNPs. Row 3: r^2 values of the plot bars in row 1 are reported. The red arrow marks rs964184.

rs964184 is relatively independent with no SNPs with r^2 > 0.52. If any, rs964184 might rather tag variant in *APOA5*, while *APOA4* variants show very low r^2 (max r^2=0.2). It is therefore unlikely, that 964184 will directly affect *APOA4* expression.

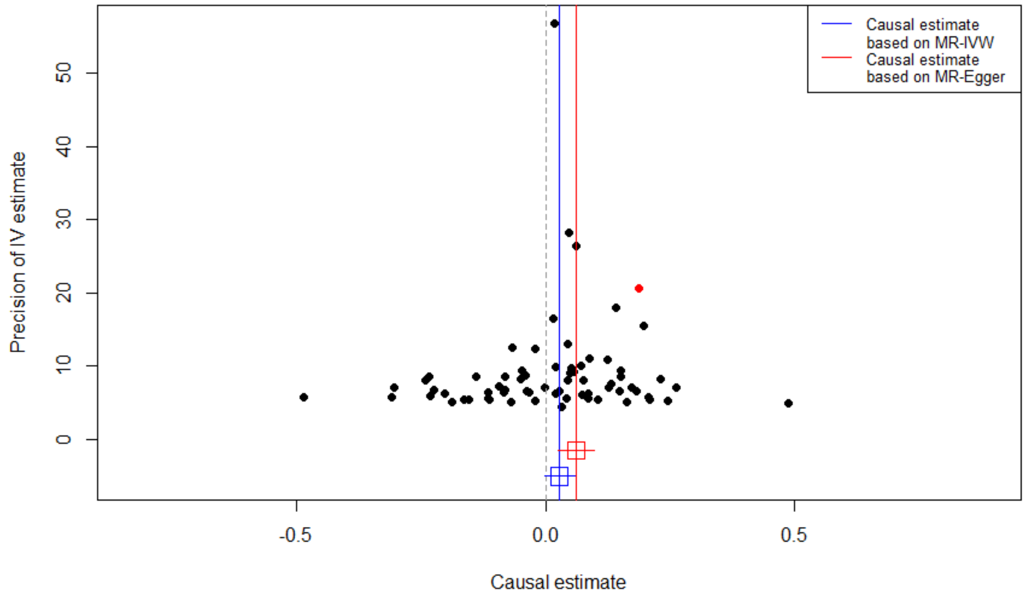

Supplementary Figure 15: Funnel plot of genetic associations with HDL-C. The MR-IVW estimate (blue) as well as the MR-Egger regression estimate (red) are shown. The causal estimates are represented by the boxes and the vertical lines, and the corresponding 95%-CIs are displayed by the horizontal lines. The red SNP denotes rs964184, the potential pleiotropic SNP identified by the gtx package.

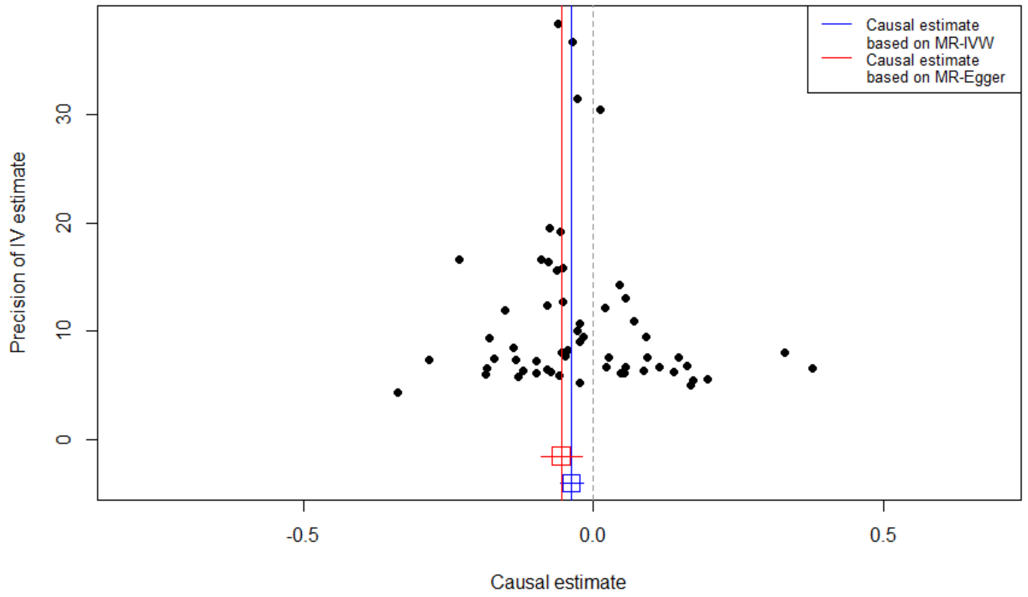

Supplementary Figure 16: Funnel plot of genetic associations with LDL-C. The MR-IVW estimate (blue) as well as the MR-Egger regression estimate (red) are shown. The causal estimates are represented by the boxes and the vertical lines, and the corresponding 95%-CIs are displayed by the horizontal lines.

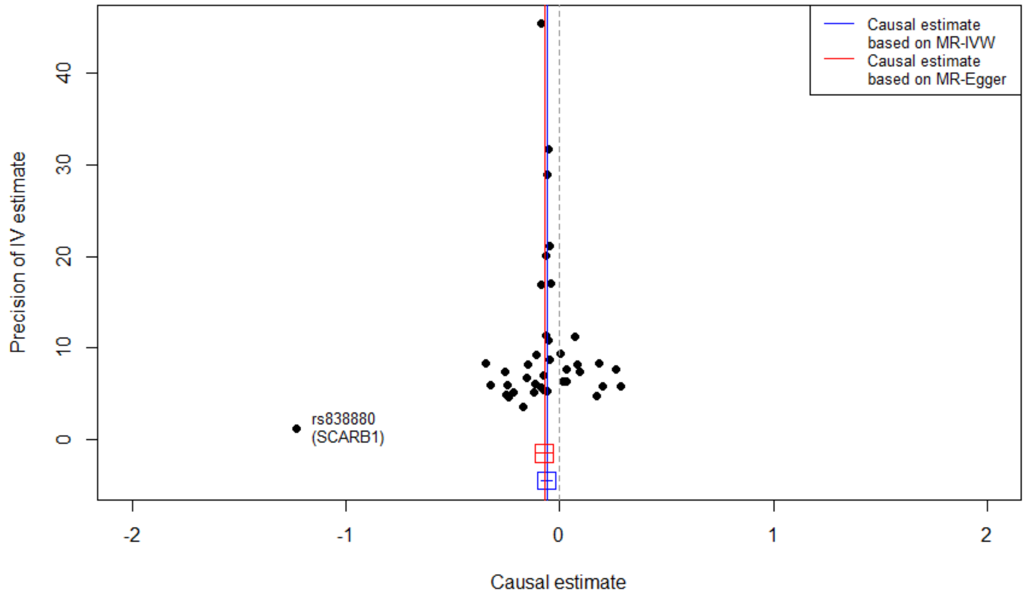

Supplementary Figure 17: Funnel plot of genetic associations with TG. The MR-IVW estimate (blue) as well as the MR-Egger regression estimate (red) are shown. The causal estimates are represented by the boxes and the vertical lines, and the corresponding 95%-CIs are displayed by the horizontal lines.

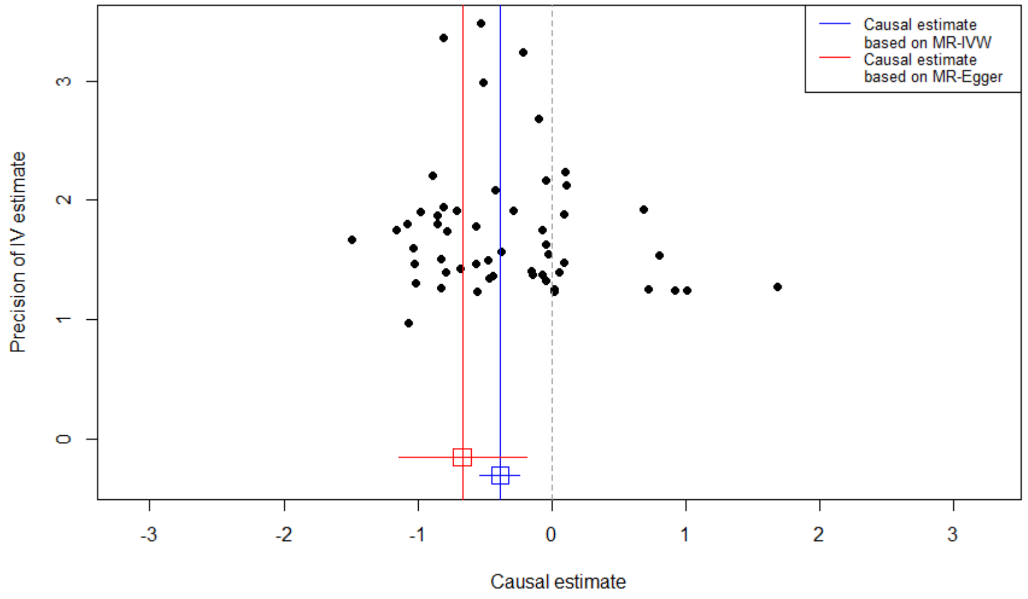

Supplementary Figure 18: Funnel plot of genetic associations with eGFR. The MR-IVW estimate (blue) as well as the MR-Egger regression estimate (red) are shown. The causal estimates are represented by the boxes and the vertical lines, and the corresponding 95%-CIs are displayed by the horizontal lines.

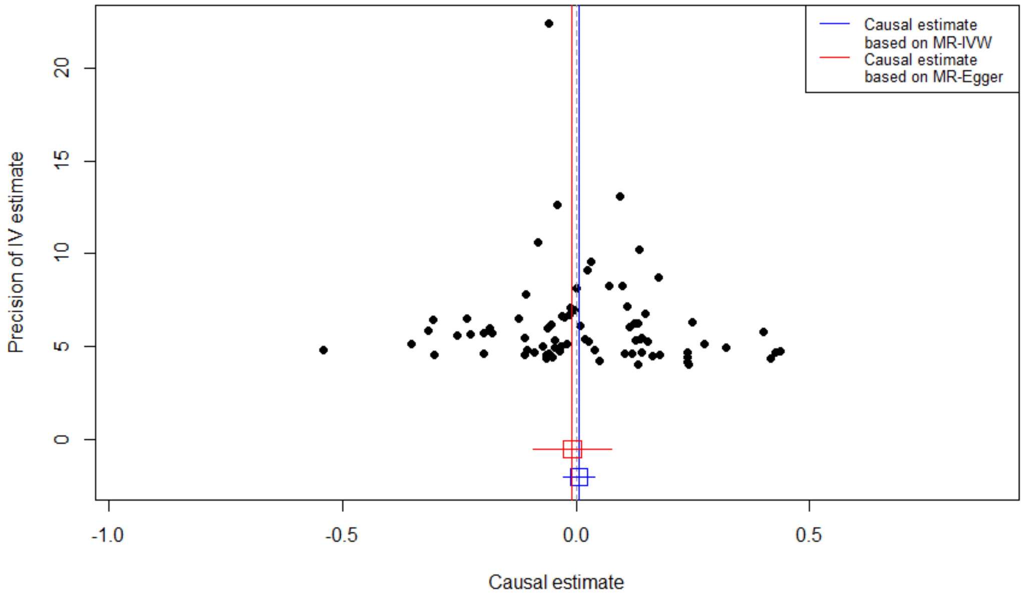

Supplementary Figure 19: Funnel plot of genetic associations with BMI. The MR-IVW estimate (blue) as well as the MR-Egger regression estimate (red) are shown. The causal estimates are represented by the boxes and the vertical lines, and the corresponding 95%-CIs are displayed by the horizontal lines.

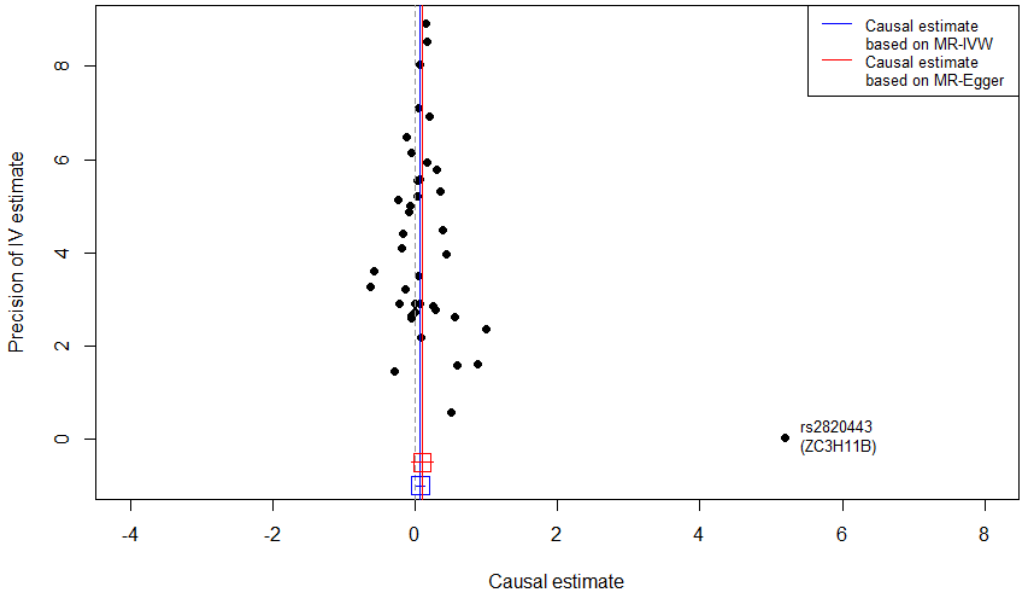

Supplementary Figure 20: Funnel plot of genetic associations with WHR. The MR-IVW estimate (blue) as well as the MR-Egger regression estimate (red) are shown. The causal estimates are represented by the boxes and the vertical lines, and the corresponding 95%-CIs are displayed by the horizontal lines.

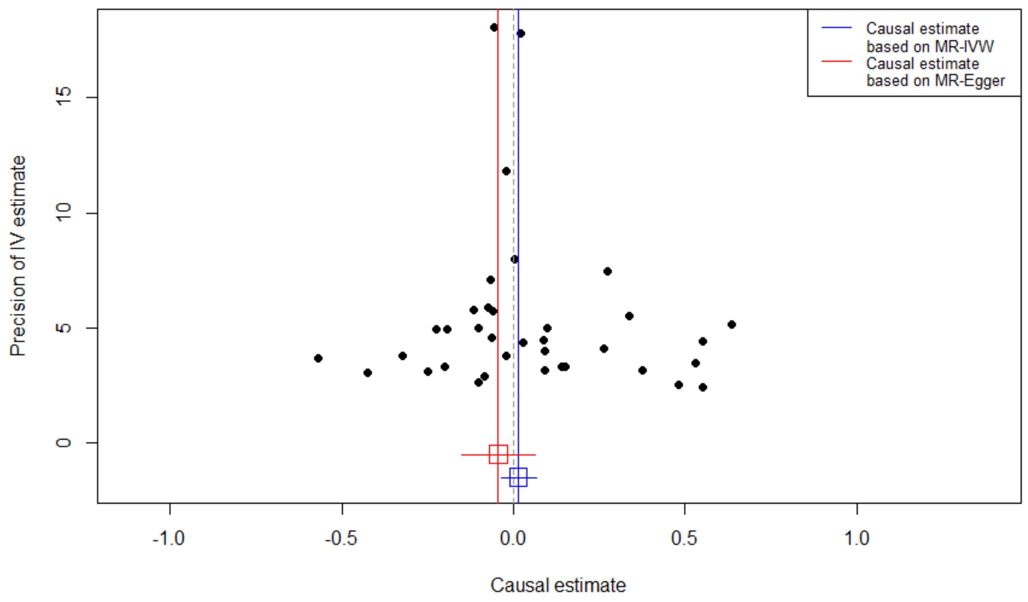

Supplementary Figure 21: Funnel plot of genetic associations with Fasting Glucose. The MR-IVW estimate (blue) as well as the MR-Egger regression estimate (red) are shown. The causal estimates are represented by the boxes and the vertical lines, and the corresponding 95%-CIs are displayed by the horizontal lines.
